# Trends in the burden of chronic diseases attributable to diet-related risk factors from 1990 to 2021 and the global projections through 2030: a population-based study

**DOI:** 10.3389/fnut.2025.1570321

**Published:** 2025-05-09

**Authors:** Huan Ma, Minyan Wang, Chu Qin, Yun Shi, Oscar Onayi Mandizadza, Haojie Ni, Conghua Ji

**Affiliations:** ^1^School of Public Health, Zhejiang Chinese Medical University, Hangzhou, Zhejiang, China; ^2^School of Human Sciences, Waseda University, Tokyo, Japan

**Keywords:** chronic diseases, diet, global burden of disease, prediction, temporal trends

## Abstract

**Background:**

The global burden of diet-related chronic diseases and their future projections remain unclear. To address this gap, we present the latest data on deaths and disability-adjusted life years attributable to dietary factors from 1990 to 2021, focusing on noncommunicable diseases worldwide. Additionally, we provide predictions of mortality rates across different age groups through 2030.

**Methods:**

Data from the Global Burden of Disease Study 2021 were analyzed to evaluate correlations between dietary factors and trends in chronic disease burden over a 30-year period. Moreover, we predicted the burden of chronic dietary diseases up to 2030.

**Results:**

From 1990 to 2021, global age-standardized mortality rates and disability-adjusted life year (DALY) rates associated with dietary factors decreased by approximately one-third for neoplasms and cardiovascular diseases (CVDs). In high sociodemographic index (SDI) regions, neoplasm-related deaths showed a stronger correlation with dietary factors, particularly high red meat intake. In cardiovascular diseases, the leading dietary factors are low-grain diets, whereas in diabetes, it is due to increased intake of processed meat. In low-SDI regions, diets low in vegetables showed the strongest association with neoplasm-related mortality, while diets low in fruits were significantly linked to CVD and diabetes burden. High-sodium diet was a significant risk factor for CVD in the middle-SDI regions. Moreover, the 2030 projections indicated a decline in mortality from neoplasms and CVDs, with a slight increase in mortality rates from diabetes.

**Conclusion:**

The global burden of chronic diseases linked to dietary factors shows varying trends across different countries and regions, particularly influenced by their economic development levels. This variation underscores the necessity of enhancing dietary structures to mitigate chronic disease prevalence and foster overall health.

## 1 Introduction

Chronic diseases are commonly known as noncommunicable diseases (NCDs), which have attracted much attention for a long time (1). The World Health Organization (WHO) classifies NCDs into four categories: cardiovascular diseases, neoplasms, chronic respiratory diseases, and diabetes (2). According to a systematic analysis of the Global Burden of Disease (GBD) Study 2019–2021, NCDs contribute to approximately 1.73 billion deaths and disability-adjusted life years (DALYs), making them the most significant health challenge facing the adult population worldwide (3). The prevalence of NCDs is projected to increase over time due to an increasingly aging population, urbanization, and lifestyle changes (4).

Diet is an important modifiable risk factor in the prevention of chronic diseases (5). Understanding and projecting the burden of chronic diseases linked to dietary habits has important public health implications (6–8). Previous studies reported current trends in the global burden of chronic diseases and identified various attributable risk factors (9–12). Additionally, they predicted future changes and trends in different chronic diseases (13, 14). However, research focusing specifically on the trends and projections of the global disease burden attributable to dietary risk factors remains scarce. This underscores the need to summarize the global trends of chronic diseases associated with specific dietary and behavioral risk factors to inform the development of specific population-based prevention and control strategies. Notably, recent studies have revealed that dietary patterns (e.g., high salt and high protein intake) also significantly influence the risk of chronic kidney disease (CKD) (15), while CKD has established pathophysiological connections with both diabetes and cardiovascular diseases. Therefore, a comprehensive assessment of the impact of diet on chronic disease burden should consider the interactions among multiple diseases.

The GBD 2021 (16) highlights the disease burdens, disability, and causes of death in 204 countries and regions (12). It is a valuable resource for evaluating and forecasting long-term trends in the disease burden associated with various risk factors. Although current evidence indicates that higher dietary quality scores (reflecting greater intake of whole grains, polyunsaturated fatty acids, nuts, and long-chain omega-3 fatty acids, along with lower intake of red/processed meats, refined grains, and sugar-sweetened beverages) are significantly associated with reduced COPD risk in both women and men (17), GBD database lacks systematic disease burden data on dietary factors and chronic respiratory diseases. Given the GBD database’s requirements for methodological consistency and data completeness in risk assessment, this study ultimately selected three chronic diseases with robust evidence for dietary attribution—neoplasms, diabetes, and cardiovascular diseases—for analysis.

Our study aimed to quantify the association between dietary risk factors and the burden of NCDs across different Socio-demographic Index (SDI) levels, and assess global trends in NCD-related deaths and DALYs attributable to diet from 1990 to 2021. We further projected mortality rates by age group through 2030 to evaluate progress toward the UN’s “2030 Sustainable Development Agenda” (18) and the WHO’s “Global Non-Communicable Diseases Covenant 2020–2030” (19). By examining how SDI mediates dietary risks, our findings provide evidence-based guidance for optimizing health resource allocation and policy interventions to reduce the global burden of diet-related NCDs.

## 2 Methods

### 2.1 Study design and period

We systematically extracted data on diet-related chronic disease burden from the Global Burden of Disease Study 2021 (GBD 2021) through the Global Health Data Exchange (GHDx)^1^ (20). Comprehensive details on data collection, processing, and modeling methods have been fully explained in previous studies (3, 21). In this study, we present the long-term trends in chronic diseases attributable to diet-related risk factors from 1990 to 2021 and project the disease burden through 2030.

### 2.2 Location and population

We selected three major chronic diseases (cardiovascular disease, neoplasm, and diabetes). Chronic respiratory diseases were excluded from our analysis as they are not associated with diet-related risk factors found in the GBD database. We considered diet-related risk as a factor, with death and DALY as outcome measures. All 204 countries were categorized into 5 Social Development Index (SDI) regions, which were further divided into 5 groups based on their SDI. Additionally, these 204 countries were organized into 21 regions based on epidemiological similarities and geographic continuity, including high-income Asia-Pacific, Central Asia, and other regions identified in the GBD study.

### 2.3 Dietary factors and disease information

Fifteen diet-related risk factors associated with chronic diseases were evaluated in the GBD study: “Diet high in processed meat,” “Diet high in red meat,” “Diet high in sodium,” “Diet high in sugar-sweetened beverages,” “Diet high in trans fatty acids,” “Diet low in calcium,” “Diet low in fiber,” “Diet low in fruits,” “Diet low in legumes,” “Diet low in nuts and seeds,” “Diet low in omega-6 Polyunsaturated Fatty Acids,” “Diet low in seafood omega-3 fatty acids,” “Diet low in milk,” “Diet low in vegetables,” and “Diet low in whole grains.” The exposure definition and optimal level (or range) of intake have been reported in detail elsewhere (21). Moreover, GBD’s comparative risk assessment framework systematically evaluated mediation pathways linking dietary exposures to health outcomes via intermediate factors. The dietary exposure level was the theoretical minimum risk exposure levels (TMRELs). An overarching limitation in the calculation of dietary TMRELs was that only direct risk-outcome relationships are considered in calculation; mediation is not accounted for.

The burden of disease index encompassed several metrics, including the summary exposure value (SEV), death, DALYs, age-standardized mortality rate (ASMR), age-standardized DALY rate (ASDR), and population attribution score (ASPAF). SEV provides a standardized measure of population-level risk factor exposure, ranging from 0 (indicating no excess risk) to 1 (representing maximum possible risk). Disease burden is quantified through DALYs, which combine years of life lost (YLLs) to premature mortality with years lived with disability (YLDs). For comparative analyses, the ASMR expresses deaths per 100,000 population using the GBD reference age structure, while the ASDR similarly adjusts DALYs per 100,000 population to account for demographic variations. The ASPAF estimates the proportion of disease burden attributable to dietary risks after adjusting for population age structure based on the GBD standard population. Additionally, attributable deaths and DALYs were estimated by multiplying the overall death rate or DALYs by the population attribution fraction (PAF) for each risk-outcome pair, accounting for factors such as age, sex, cause, and location. The SEV, along with the number, rate, and PAF of diet-related chronic diseases were also directly sourced from the GHDx.

### 2.4 Statistical analyses

The burden of chronic diseases due to diet-related risk factors was analyzed, adjusting for age, sex, year, and location. To account for demographic differences, we used the age-standardized rate and DALYs to compare variations across different populations and within the same population over time. We employed an integrated nested Laplacian approximation (R-packaged BAPC and INLA) to implement a Bayesian age group model for predicting ASMR for diet-related chronic diseases from 2021 to 2030 (22). We section the specific prior distributions used (default INLA priors with precision parameters following logGamma (1, 0.0005)), convergence diagnostics (all parameters had Gelman-Rubin statistics <1.05 and effective sample sizes >1000), and validation metrics (10-fold cross-validation showing mean absolute error of 2.1 deaths/100,000). This approach aligns with the Guidelines for Accurate and Transparent Health Estimation Reporting (23). In addition, we applied the Joinpoint regression model (Version4.7, National Cancer Institute) to assess the trends of the age-standardized SEV, ASMR, ASDR, and ASPAF from 1990 to 2021. Based on the Joinpoint program’s recommendation of allowing 1 joinpoint per 5–7 years of data and the conservative principle of (total years/5)-1, we set the maximum number of joinpoints to 5 for our 32-year study period. Model selection was conducted using the Bayesian Information Criterion (BIC) with a penalty factor of k = ln(n), supplemented by permutation tests (9,000 replicates, *α* = 0.05) to validate the identified trend changes. We required a minimum interval of 3 years between adjacent joinpoints to ensure clinical interpretability. To comprehensively characterize the SDI-ASMR relationship while ensuring robust results, we implemented a dual-model analytical approach combining Gaussian process (GP) regression and Loess smoothing. The GP regression, employing a Matérn 3/2 kernel, provided probabilistic uncertainty quantification and captured global nonlinear patterns, while the Loess model (span = 0.7, bandwidth optimized via AICc) enabled detection of localized variations without parametric assumptions. This complementary framework served three key purposes: (1) cross-validation through model concordance analysis (with 84% of SDI values demonstrating overlapping 95% CIs between the two models), (2) mitigation of overfitting risks by requiring consistent directional trends in both models for interpretation while flagging discordant regions (16% of points) as indeterminate, and (3) enhanced robustness verification through 10-fold cross-validation (GP RMSE = 2.3 vs. Loess RMSE = 2.7). This conservative approach balanced the strengths of parametric and non-parametric methods while minimizing potential overinterpretation of random fluctuations as meaningful signals (24). We calculated the annual percentage change (APC), average APC (AAPC), and 95% confidence intervals (CIs). All statistical analyses were performed using R (version 4.3), with the significance threshold set at *p* < 0.05.

## 3 Results

### 3.1 Global burden of chronic diseases attributable to diet

From 1990 to 2021, the all-age SEV and age-standardized SEV exhibited a downward trend, with percentage changes of −4.86 and −6.69%, respectively (Table 1). The chronic disease burden index included estimated population figures, all-age PAF, ASPAF, mortality, and DALYs. For neoplasms, while the estimated number of deaths and DALYs showed increasing trends, the other indicators decreased. In cardiovascular diseases, although the estimated number of deaths and percentage of DALYs significantly increased from 1990 to 2021 by 44.82 and 36.46%, respectively, the ASMR and ASDR significantly declined, along with other disease burden indices. For diabetes, most indices showed an increasing trend except for PAF, ASPAF, and ASMR for death, which decreased. Particularly noteworthy were the estimated increases in deaths and DALYs, which rose by 132.48 and 196.84%, respectively.

**Table 1:** The summary exposure values and burden of neoplasms, cardiovascular diseases, and diabetes mellitus attributable dietary risks.

Metric/measure	1990 values (95%CI)	2021 values (95%CI)	Percentage change (%)
Summary exposure values			
All ages, %	39.66(30.29, 49.33)	37.73(28.12, 47.95)	−4.86(−9.29, −0.44)
Age-standardized, %	40.31(30.73, 50.09)	37.62(28.05, 47.81)	−6.69(−10.19, −2.96)
Neoplasms			
Death			
Number	463979.47(125175.75, 864966.82)	669656.23(207110.92, 1176736.66)	44.33(26.86, 68.19)
PAF (all ages), %	8.01(2.22, 15.27)	6.77(2.20, 12.05)	−15.56(−24.46, −1.91)
ASPAF, %	8.25(2.31, 15.61)	6.78(2.21, 12.05)	−17.84(−26.23, −5.5)
Mortality (all ages), 1/10^5^	8.70(2.35, 16.22)	8.49(2.62, 14.91)	−2.45(−14.26, 13.68)
ASMR, 1/10^5^	12.24(3.32, 22.78)	7.90(2.45, 13.85)	−35.45(−42.94, −25.38)
DALYs			
Number	12377666.46(23260291.81, 3287466.84)	16403608.55(4939614.02, 29035003.97)	32.53(14.96, 52.77)
PAF (all ages), %	7.28(1.96, 13.98)	6.47(2.01, 11.53)	−11.12(−22.73, 0.25)
ASPAF, %	7.61(2.07, 14.57)	6.42(1.99, 11.44)	−15.74(−26.52, −5.25)
All-age rate, 1/10^5^	232.07(61.64, 436.11)	207.87(62.60, 367.93)	−10.43(−22.3, 3.25)
ASDR, 1/10^5^	302.48(80.53, 565.63)	189.62(57.13, 335.37)	−37.31(−45.38, −27.95)
Cardiovascular diseases			
Death			
Number	4028353.83(1114890.64, 5748940.07)	5833851.36(1357128.54, 8661541.28)	44.82(30.13, 57.22)
PAF (all ages), %	32.65(9.16, 46.89)	30.02(7.31, 44.59)	−8.04(−16.29, −3.70)
ASPAF, %	31.70(8.80, 46.02)	29.66(7.21, 44.17)	−6.44(−13.2, −2.00)
Mortality (all ages), 1/10^5^	75.53(20.90, 107.79)	73.93(17.20, 109.76)	−2.12(−12.05, 6.26)
ASMR, 1/10^5^	113.61(31.19, 164.63)	69.81(16.19, 104.09)	−38.55(−44.01, −32.97)
DALYs			
Number	98331777.39(27092344.68, 136036264.49)	134179727.74(32581869.94, 192859591.16)	36.46(19.4, 46.8)
PAF (all ages), %	33.03(9.14, 46.02)	31.31(7.80, 45.21)	−5.22(−16.05, −0.85)
ASPAF, %	32.93(9.15, 46.37)	30.91(7.69, 44.69)	−6.11(−16.47, −2.41)
All-age rate, 1/10^5^	1843.62(507.95, 2550.54)	1700.34(412.88, 2443.94)	−7.77(−19.3, −0.78)
ASDR, 1/10^5^	2487.47(675.52, 3480.84)	1563.86(378.95, 2246.75)	−37.13(−44.85, −32.23)
Diabetes mellitus			
Death			
Number	164060.50(30935.04, 265372.47)	381415.56(74327.89, 620913.76)	132.48(114.5, 147.18)
PAF (all ages), %	24.41(4.63, 39.50)	23.00(4.49, 37.52)	−5.76(−8.17, −0.86)
ASPAF, %	25.05(4.77, 40.46)	23.02(4.50, 37.53)	−8.09(−10.3, −3.63)
Mortality (all ages), 1/10^6^	3.08(0.58, 4.98)	4.83(0.94, 7.87)	57.13(44.98, 67.06)
ASMR, 1/10^5^	4.55(0.86, 7.35)	4.52(0.88, 7.36)	−0.72(−7.32, 5.46)
DALYs			
Number	6450217.18(1256121.72, 10613944.57)	19146810.25(4147238.97, 31937618.10)	196.84(175.83, 218.72)
PAF (all ages), %	23.42(4.44,37.79)	24.19(4.92, 39.10)	3.32(0.57, 9.99)
ASPAF, %	24.09(4.56, 38.87)	24.10(4.90, 38.94)	100.63(86.43, 115.42)
All-age rate, 1/10^5^	120.93(23.55, 199.00)	242.63(52.55, 404.72)	0.04(−2.34, 6.7)
ASDR, 1/10^5^	159.96(31.17, 262.82)	221.34(47.97, 368.92)	38.38(28.07, 49.41)

PAF, population-attributable fraction; ASPAF, age-standardized population-attributable fraction; ASMR, age-standardized mortality rate; DALY, disability-adjusted life year; ASDR, age-standardized disability-adjusted life year rate.

**Figure 1:**
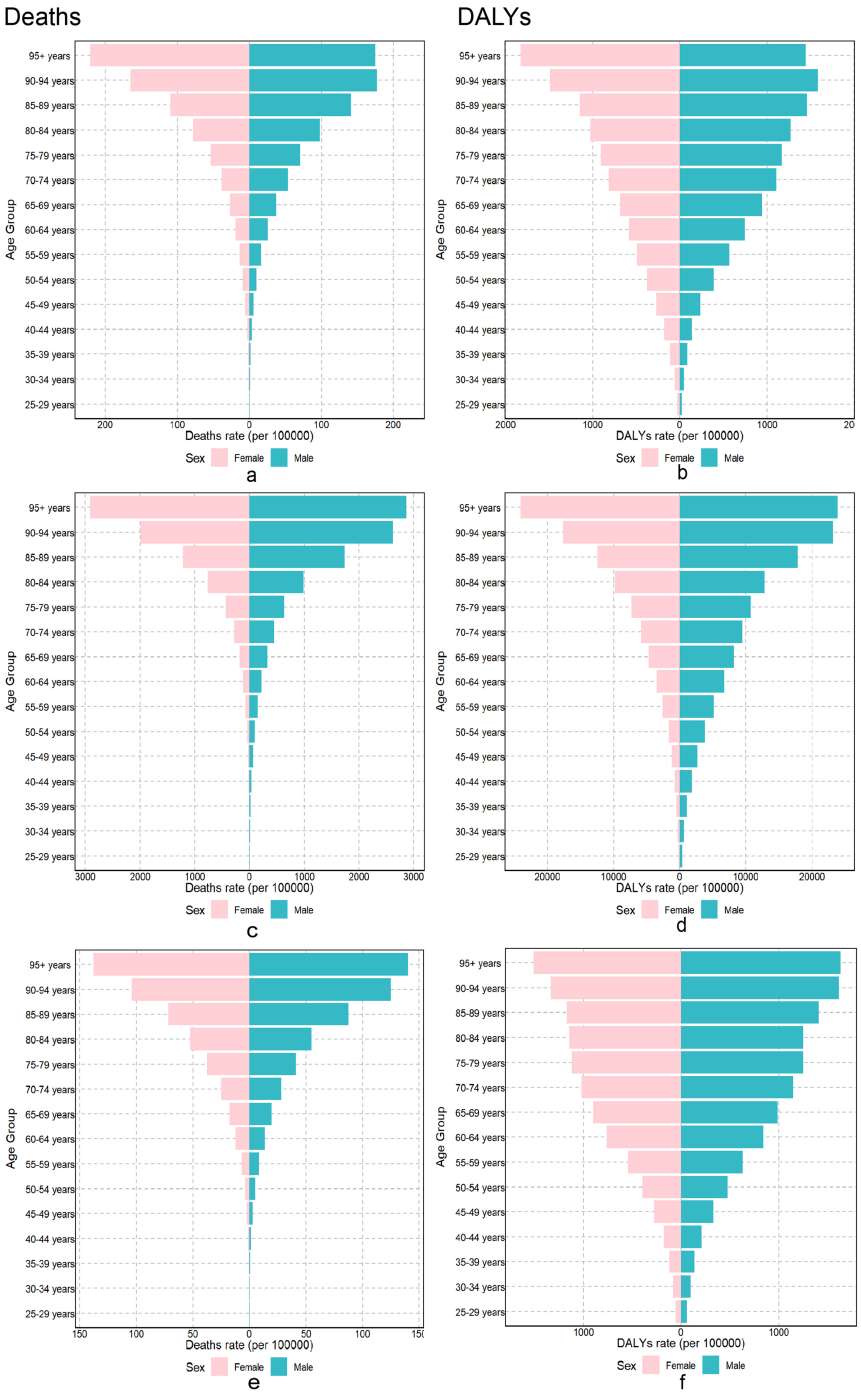
The burden of chronic diseases attributable to diet in different gender and age groups in 2021. Neoplasms Cardiovascular disease and Diabetes mellitus burden attributable to dietary risks among different genders and age groups in 2021. Neoplasms: **(a)** Age-standardized death rates. **(b)** Age-standardized DALY rates. Cardiovascular disease: **(c)** Age-standardized death rates. **(d)** Age-standardized DALY rates. Diabetes mellitus: **(e)** Age-standardized death rates. **(f)** Age-standardized DALY rates. DALY, disability-adjusted life year.

Globally, in 2021, the burden of chronic diseases attributable to dietary factors varied markedly between sexes and across different age groups. Men had more burden of diet-related chronic diseases compared to women (Figure 1). Age-specific diet-related chronic diseases, DALYs, and mortality rates increased with advancing age.

### 3.2 Overall effect of diet on death and DALYs

In 2021, we examined the association between dietary risk factors and chronic disease mortality and disability-adjusted life years (DALYs) across 204 countries and regions (Supplementary Tables S1–S3; Supplementary Figure S1). Lesotho had the highest rates of diet-related neoplasm and DALYs, with 16.69 [5.28–28.00] deaths per 100,000 population and 439.34 [129.18–748.79] deaths per 100,000 population, respectively. Afghanistan had the highest diet-related cardiovascular mortality with 240.75 [53.43–362.41] deaths per 100,000 population, while Nauru recorded the highest diet-related cardiovascular DALY mortality at 6,306.64 [340.04–10244.29] deaths per 100,000 population. For diabetes, Fiji had the highest diet-related mortality rates and DALYs at 69.84 [10.94–121.19] deaths per 100,000 population and 1,978.51 [322.59–3413.58] deaths per 100,000 population, respectively.

Conversely, Cote d’Ivoire had the lowest rates of diet-related neoplasm and DALY mortalities of 0.77 [−3.45–3.18] deaths per 100,000 population, and 43.26 [−38.15–89.50] deaths per 100,000 population, respectively. Israel reported the lowest diet-related cardiovascular and DALY mortality rates at 12.23 [3.70–19.88] deaths per 100,000 population and 239.78 [77.41–372.08] deaths per 100,000 population, respectively. For diabetes, Singapore had the lowest diet-related mortality rate at 0.43 [0.07–0.74] deaths per 100,000 population, while North Korea reported the lowest diet-related DALY mortality rate of 89.72 [16.71–157.68] deaths per 100,000 population.

Over the past three decades, among the 204 countries and regions evaluated, chronic disease mortality attributable to dietary risk factors declined in 72 countries, whereas DALYs decreased in only 24 countries. However, only 16 out of 204 countries and regions had increasing chronic disease mortality rates attributable to dietary risk factors, with just 14 showing an increase in DALYs (Supplementary Tables S4, S5).

**Figure 2:**
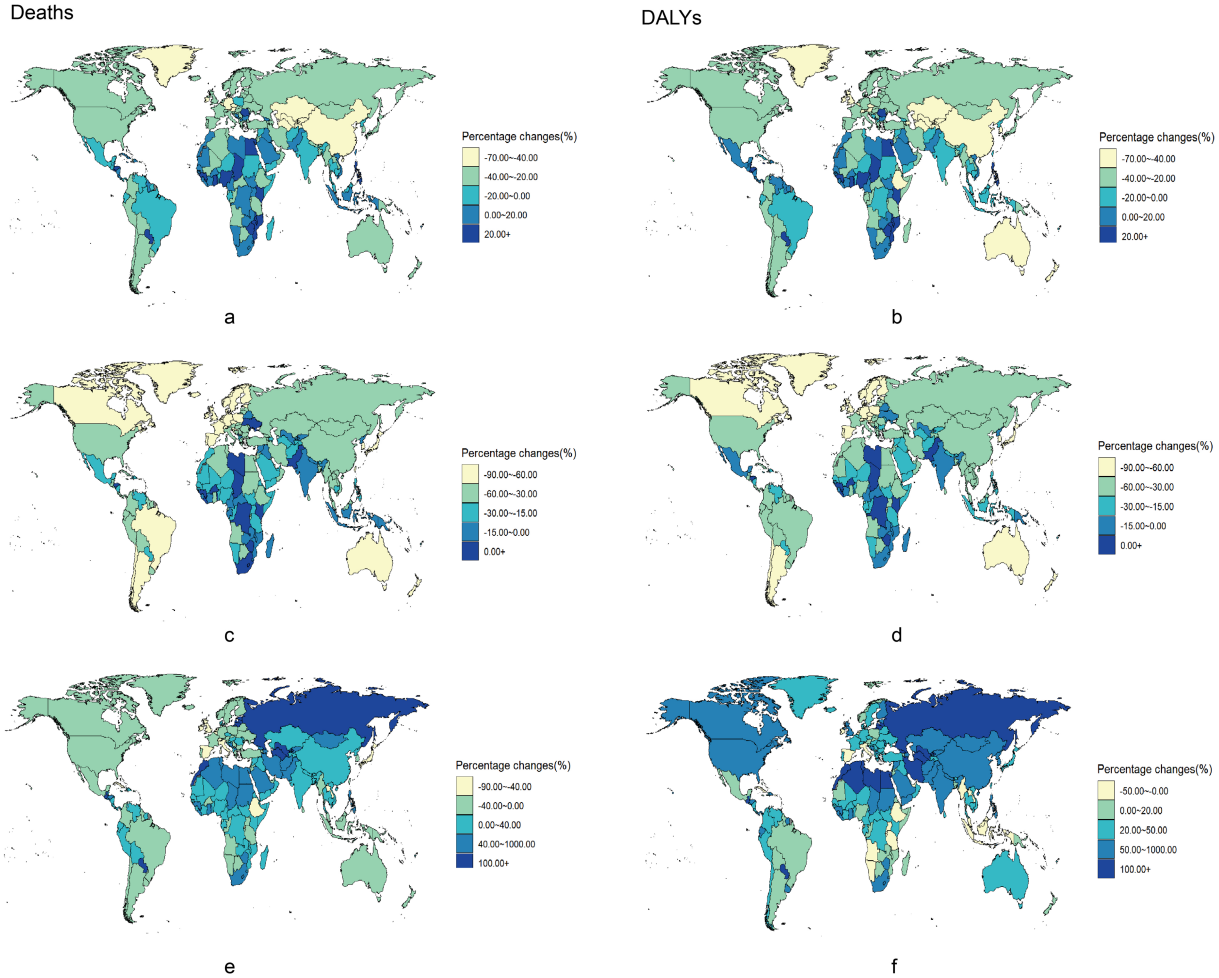
Percentage changes of chronic diseases burden attributable to dietary risks among 204 countries and territories from 1990 to 2021. Percentage changes of Neoplasms, Cardiovascular disease and Diabetes mellitus burden attributable to dietary risks among 204 countries and territories form 1990 to 2021. Neoplasms: **(a)** Age-standardized death rates. **(b)** Age-standardized DALY rates. Cardiovascular disease: **(c)** Age-standardized death rates. **(d)** Age-standardized DALY rates. Diabetes mellitus: **(e)** Age-standardized death rates. **(f)** Age-standardized DALY rates. DALY, disability-adjusted life year.

Figure 2 illustrates the ASMR and ASDR changes for chronic diseases from 1990 to 2021 across 204 countries and regions. Notably, three diseases exhibited increasing ASMR and ASDR only in Sub-Saharan Africa. In contrast, in high-income regions, such as North America and Western Europe, the ASMR and ASDR decreased over time (Supplementary Tables S6, S7). Central Asia noticeably reported the lowest ASMR and ASDR for cardiovascular diseases (Supplementary Tables S6, S7).

### 3.3 Effects of individual dietary components on mortality and DALYs

**Figure 3:**
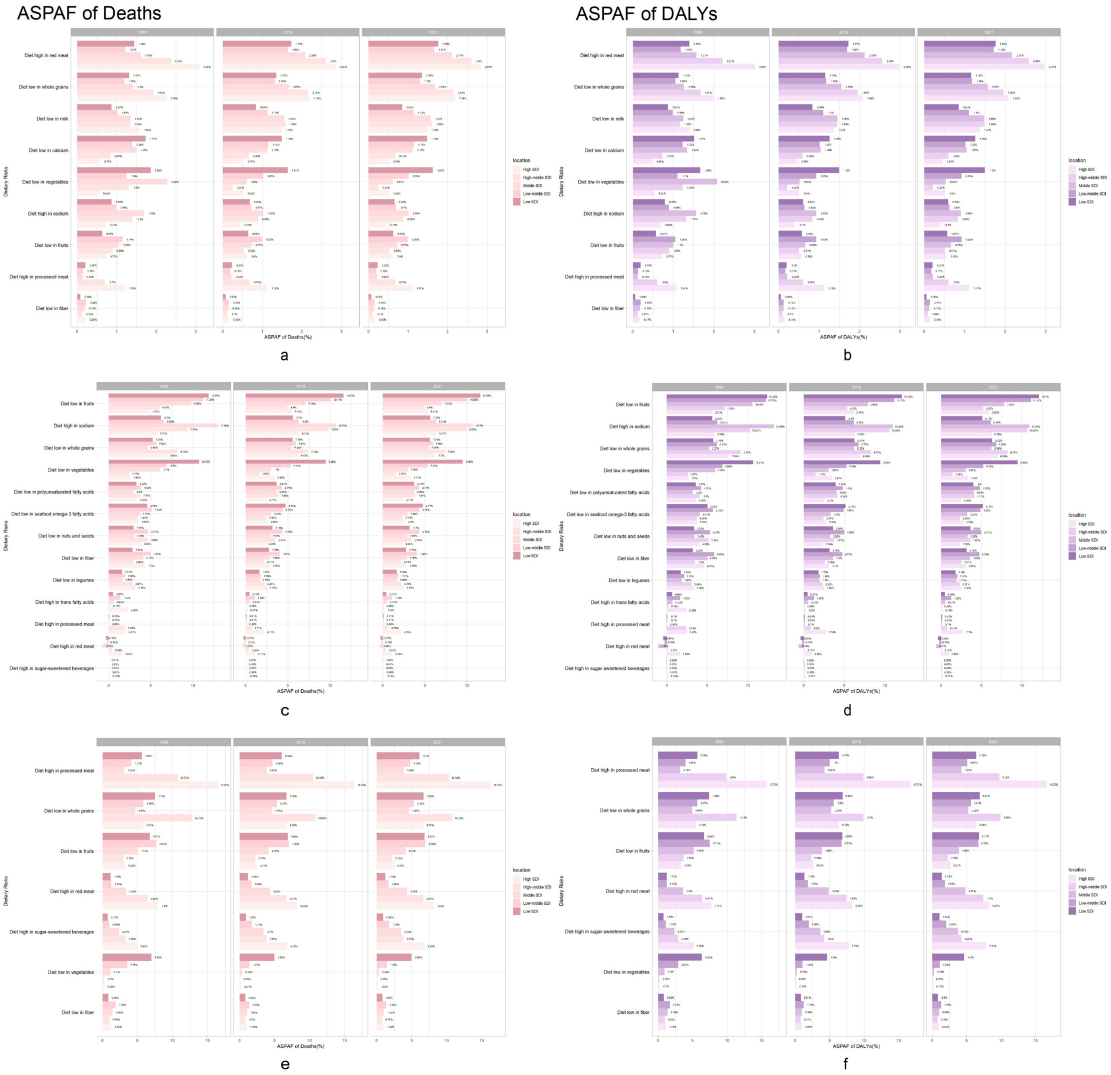
Chronic diseases burden attributable to dietary risks in 1990, 2019 and 2021. Neoplasms, Cardiovascular disease and Diabetes mellitus burden population attributable proportion in 1990, 2019 and 2021 across 5 GBD SDI regions. Neoplasms: **(a)** Age-standardized population attributable proportion of death rates. **(b)** Age-standardized population attributable proportion of DALY rates. Cardiovascular disease: **(c)** Age-standardized population attributable proportion of death rates. **(d)** Age-standardized population attributable proportion of DALY rates. Diabetes mellitus: **(e)** Age-standardized population attributable proportion of death rates. **(f)** Age-standardized population attributable proportion of DALY rates. DALY, disability-adjusted life year.

In 1990, the main diet-related risk factor for neoplasm-related death and DALYs in high- and medium-high SDI countries was excess consumption of red meat (Figure 3). Conversely, in medium-SDI, low-moderate SDI, and low-SDI countries, limited vegetable intake was a significant risk factor for neoplasm-related deaths and DALYs. In 2021, excess red meat consumption emerged as the dietary factor with the strongest association with neoplasm-related mortality and DALYs globally (population attributable fraction), despite its declining prevalence in high-SDI countries. In 1990, the main diet-related risk factor for cardiovascular deaths and DALYs in high-SDI countries was low whole grain intake, accounting for 6.94 and 7.68%, respectively. In medium-high and medium-SDI countries, the major dietary risk factor for cardiovascular death and DALYs was high-sodium intake, while in medium-low and low-SDI countries it was low intake of fruits. By 2021, the major dietary risk factors for cardiovascular deaths and DALYs remained unchanged compared to 2019 across all SDI groups, although their prevalence had decreased.

In 1990, the main dietary risk factor for diabetes-related deaths and DALYs in high-SDI countries was excess consumption of processed meat, accounting for 16.47 and 15.75%, respectively. Conversely, in medium-high and low-SDI countries, low intake of whole grains was the major diet-related risk factor. In medium-low and moderate-low SDI countries, low intake of fruits was the major diet-related risk factor. By 2021, the main dietary risk factors for diabetes-related deaths and DALYs in middle SDI countries shifted to low intake of whole grains, which increased in prevalence, while the main diet-related risk factors in other regions remained unchanged, but showed a decrease in their proportions.

### 3.4 Temporal trends in chronic diseases attributable to dietary factors between 1990 and 2021

#### 3.4.1 Temporal trends in global age-standardized mortality and DALYs for chronic diseases between 1990 and 2021

Table 2 presents global trends in age-standardized mortality and DALYs for chronic diseases from 1990 to 2021. The AAPC for neoplasm-related ASMR was −1.40 (95% CI, −1.48 to 1.32), and the AAPC for ASDR was −1.49 (95% CI, −1.57 to 1.42). Regression analysis of junction points revealed a negative APC for both neoplasm ASMR and ASDR (*p* < 0.05) (Supplementary Figures S2a–d), indicating a consistent decline in neoplasm-related ASMR and ASDR from 1990 to 2021, with the most significant decrease occurring between 2004 and 2007. For cardiovascular diseases, the AAPCs of ASMR and ASDR were −1.57 (95% CI, −1.68 to −1.43) and −1.47(95% CI, −1.61 to −1.34), respectively. The time curve indicates that both ASMR and ASDR for cardiovascular diseases initially decreased slightly, followed by a significant decline from 2003 to 2007, then with more moderate declines in recent years (Supplementary Figures S2b,e). In contrast, the AAPCs for diabetes-related ASMR was −0.01 (95% CI, −0.13-0.10), which is notably lower compared to AAPCs for ASDR; 1.06 (95% CI, 0.99–1.13). From 1990 to 2021, diabetes-related ASMR experienced several fluctuations, with periods of increasing and decreasing; however, there was no significant overall change (Supplementary Figure S2c). Notably, unlike ASMR, the APC for diabetes-related ASDR was positive (*p* < 0.05) (Supplementary Figure S2f), indicating a steady annual increase in diabetes-related ASDR over the past three decades.

**Table 2:** Joinpoint analysis of temporal trends in the global burden of chronic diseases attributable to dietary risks from 1990 to 2021.

Metric	Trend 1		Trend 2		Trend 3		Trend 4		Trend 5		Trend 6		1990–2021
	Period	APC, % (95% CI)	Period	APC, % (95% CI)	Period	APC, % (95% CI)	Period	APC, % (95% CI)	Period	APC, % (95% CI)	Period	APC, % (95% CI)	AAPC, % (95% CI)
Age-standardized mortality rate													
Neoplasms	1990–1994	−0.82 (−1.00, −0.64)	1994–1997	−1.92 (−2.47, −1.37)	1997–2004	−1.38 (−1.47, −1.25)	2004–2007	−2.55 (−3.08, −2.01)	2007–2014	−1.55 (−1.64, −1.46)	2014–2021	−0.88 (−0.95, −0.81)	−1.40 (−1.48, −1.32)
Cardiovascular diseases	1990–1994	−0.23 (−0.60, 0.13)	1994–1998	−2.23 (−2.83, −1.69)	1998–2003	−1.13 (−1.50, −0.76)	2003–2007	−2.67 (−3.25, −2.08)	2007–2021	−1.56 (−1.62, −1.51)			−1.57 (−1.68, −1.43)
Diabetes mellitus	1990–1995	0.82 (0.61, 1.02)	1995–2000	−0.23 (−0.51, 0.05)	2000–2003	0.97 (0.07, 1.88)	2003–2012	−0.97 (−1.06, −0.87)	2012–2019	0.55 (0.40, 0.71)	2019–2021	−0.66 (−1.55,0.24)	−0.01 (−0.13,0.10)
Age-standardized population attributable proportion of death rates													
Neoplasms	1990–1993	−0.55 (−0.66, −0.43)	1993–2000	−0.92 (−0.96, −0.89)	2000–2004	−0.66 (−0.78, −0.55)	2004–2008	−0.95 (−1.06, −0.84)	2008–2014	−0.64 (−0.69, −0.59)	2014- 2021	−0.19 (−0.21, −0.16)	−0.64 (−0.66, −0.61)
Cardiovascular diseases	1990–1994	−0.017 (−0.08, 0.05)	1994–1998	−0.49 (−0.60, −0.38)	1998–2004	−0.34 (−0.38, −0.27)	2004–2009	−0.26 (−0.33, −0.17)	2009–2013	0.038 (−0.07, 0.15)	2013–2021	−0.19 (−0.22, −0.17)	−0.22 (−0.24, −0.19)
Diabetes mellitus	1990–1998	−0.26 (−0.29, −0.23)	1998–2002	−0.13 (−0.25, 0.00)	2002–2009	−0.41 (−0.46, −0.37)	2009–2013	−0.58 (−0.70, −0.45)	2013–2017	0.02 (−0.11, 0.14)	2017–2021	−0.19 (−0.27, −0.11)	−0.27 (−0.31, −0.24)
Age-standardized DALY rate													
Neoplasms	1990–1994	−0.93 (−1.11, −0.75)	1994–1997	−2.18 (−2.72, −1.64)	1997–2004	−1.58 (−1.67, −1.49)	2004–2007	−2.64 (−3.17, −2.11)	2007–2014	−1.62 (−1.71, −1.53)	2014–2021	−0.81 (−0.88, −0.74)	−1.49 (−1.57, −1.42)
Cardiovascular diseases	1990–1994	−0.01 (−0.39, 0.37)	1994–1998	−2.23 (−2.82, −1.65)	1998–2003	−1.06 (−1.44, −0.67)	2003–2007	−2.56 (−3.15, −1.96)	2007–2015	−1.69 (−1.85, −1.53)	2015–2021	−1.27 (−1.48, −1.06)	−1.47 (−1.61, −1.34)
Diabetes mellitus	1990–1994	1.46 (1.29, 1.62)	1994–2000	0.98 (0.87, 1.09)	2000–2003	1.34 (0.82, 1.86)	2003–2012	0.52 (0.46, 0.57)	2012–2019	1.52 (1.39, 1.57)	2019–2021	2.52 (0.57, 1.59)	1.06 (0.99, 1.13)
Age-standardized population-attributable proportion of DALY rates													
Neoplasms	1990–1993	−0.49 (−0.61, −0.36)	1993–2000	−0.91 (−0.95, −0.87)	2000–2004	−0.57 (−0.69, −0.44)	2004–2008	−0.89 (−1.01, −0.77)	2008–2014	−0.54 (−0.59, −0.48)	2014–2021	−0.04 (−0.07, −0.01)	−0.56 (−0.58, −0.53)
Cardiovascular diseases	1990–1994	0.077 (−0.02, 0.17)	1994–1998	−0.52 (−0.67, −0.38)	1998–2001	−0.23 (−0.53, 0.06)	2001–2009	−0.38 (−0.42, −0.36)	2009–2012	0.10 (−0.20, 0.41)	2012–2021	−0.13 (−0.16, −0.11)	−0.21 (−0.25, −0.16)
Diabetes mellitus	1990–1997	−0.1 (−0.11, −0.09)	1997–2004	0.12 (0.10, 0.14)	2004–2013	−0.04 (−0.05, −0.02)	2013–2017	0.13 (−0.07, 0.18)	2017–2021	−0.04 (−0.08, −0.01)			0.00 (−0.01, 0.01)

APC, annual percentage change; AAPC, average annual percentage change; DALY, disability-adjusted life year.

#### 3.4.2 Temporal trends in the proportion of dietary attributable deaths and DALYs in the global age-standardized population for chronic diseases between 1990 and 2021

Table 2 shows temporal trends in the proportion of chronic disease-related deaths and DALYs associated with dietary risk factors in the global age-standardized population from 1990 to 2021. The AAPC and 95% CI of ASPAF for neoplasm deaths and DALYs were −0.64 (−0.66 to −0.61) and 0.56 (−0.58–0.53), respectively. Supplementary Figures S3a,d illustrate that the APC for ASPAF related to neoplasm death and DALYs is negative, mirroring the trends in mortality and DALYs rates, which indicates an annual decline in the proportion of deaths and DALYs associated with dietary factors after adjusting for age. For cardiovascular diseases, the AAPC and 95% CI for the ASPAF and DALYs were −0.22 (−0.24–0.19) and −0.21 (−0.25–0.16), respectively. As shown in Supplementary Figures S3b,e, the APCs for ASPAF related to both cardiovascular disease deaths and DALYs were statistically significant and negative, suggesting that the proportion of deaths and DALYs associated with diet also decreased annually after adjusting for age. The AAPC and 95% CI for ASPAF concerning diabetes were −0.27 (−0.31–0.24) and 0.00 (−0.01–0.01), respectively. The ASPAF for diabetes was statistically significant, and APC was significant after adjusting for age (Supplementary Figure S3c). However, Supplementary Figure S3e shows that the proportion of age-standardized DALYs for diabetes attributable to diet-related factors remained relatively stable, showing no significant change from 1990 to 2021.

### 3.5 Analysis of dynamic association between sociodemographic index and global chronic disease mortality from 1990 to 2021

**Figure 4:**
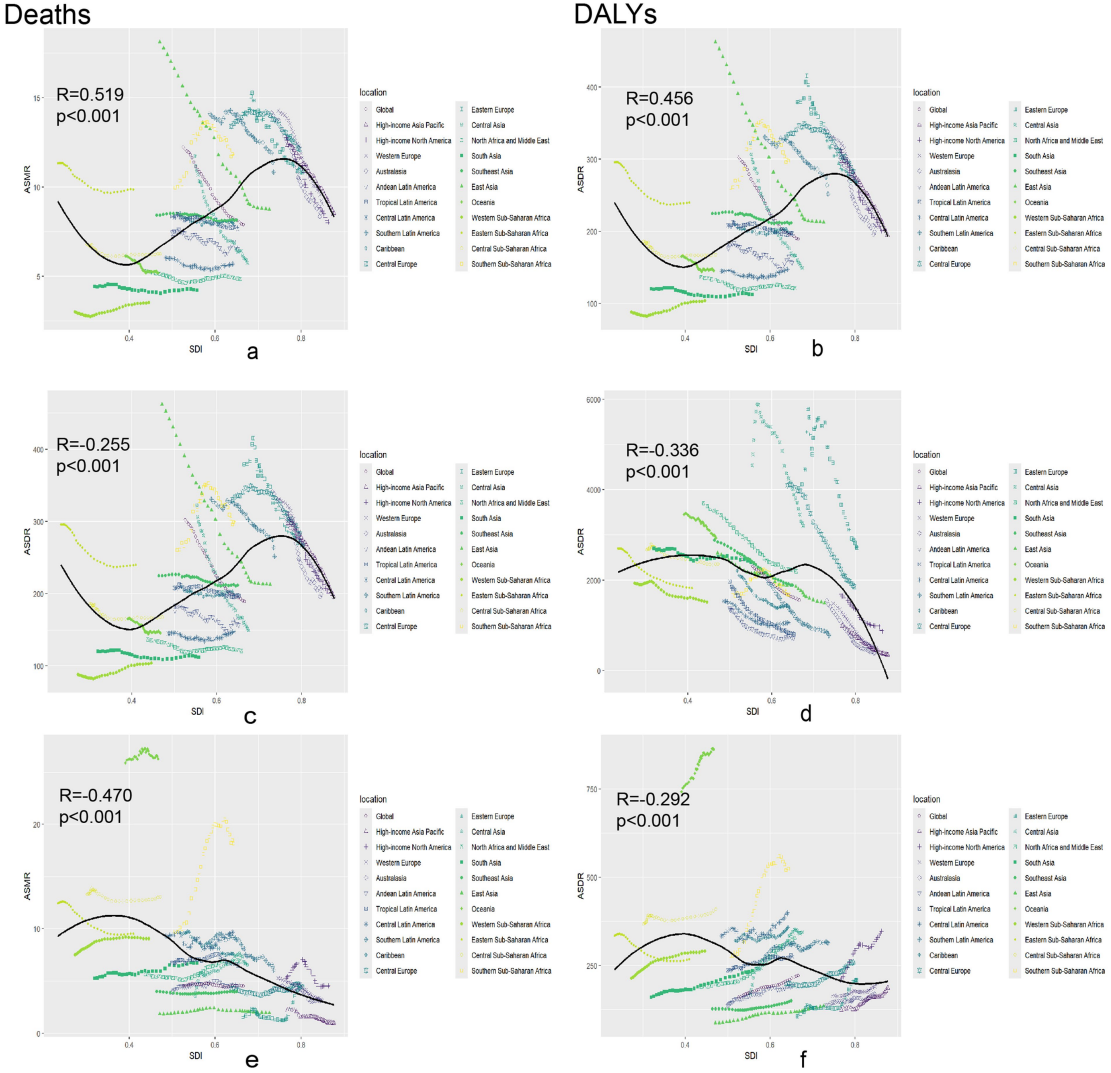
Association between age-standardized death rate or age-standardized DALY rate and SDI among 21 regions. Association between age-standardized death rate or age-standardized DALY rate and SDI among 21 regions. Neoplasms: **(a)** Age-standardized death rates. **(b)** Age-standardized DALY rates. Cardiovascular disease: **(c)** Age-standardized death rates. **(d)** Age-standardized DALY rates. Diabetes mellitus: **(e)** Age-standardized death rates. **(f)** Age-standardized DALY rates. DALY, disability-adjusted life year.

In this study, local weighted regression analysis was employed to examine the relationship between the SDI and global ASMR for chronic diseases. Figure 4 categorizes 204 countries into 21 regions based on epidemiological homogeneity and geographic continuity and illustrates the relationship between SDI and neoplasm ASMR in these regions. Figures 4a,d depict the relationships among ASMR, ASDR, and SDI for neoplasms worldwide. Data reveals that in countries with SDI < 0.6, both ASMR and ASDR remained stable as SDI increased. Conversely, in countries with SDI > 0.6, ASMR and ASDR showed a decreasing trend. Overall, there is a moderately positive correlation between ASMR, ASDR, and SDI for neoplasms, with correlation coefficients of *R* = 0.519 (*p* < 0.001) and 0.456 (*p* < 0.001), respectively. This suggests that as SDI increases, both ASMR and ASDR also increase, potentially reflecting changes in disease prevalence, lifestyle factors, or an aging population. For cardiovascular diseases, the ASMR in countries with SDI < 0.6 remained stable with the increase in SDI, whereas in countries with SDI > 0.6, ASMR decreased with increasing SDI (Figures 4b,e). However, the overall correlation trend indicates a negative correlation between ASMR, ASDR, and SDI for cardiovascular diseases, with correlation coefficients of *R* = −0.255 (*p* < 0.001) and −0.336 (*p* < 0.001), respectively. This indicates that as SDI increased, ASMR and ASDR decreased. Figures 4c,f illustrated that the global ASMR, ASDR, and SDI were negatively correlated, and as SDI increased, ASMR (*R* = −0.470, *p* < 0.001) and ASDR (*R* = −0.292, *p* < 0.001) decreased.

### 3.6 Age-standardized mortality predictions for dietary chronic diseases over the next 10 years

**Figure 5:**
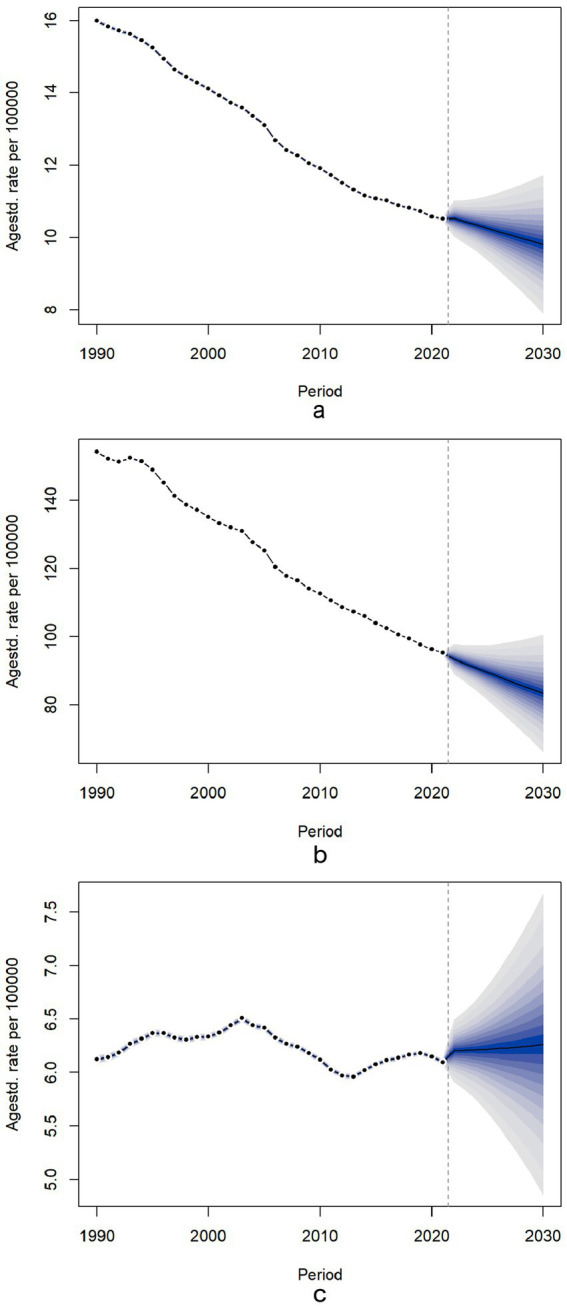
Temporal trends of for ASMR for neoplasms, cardiovascular disease and diabetes mellitus attributable to dietary risks between 1990 and 2030 in Global. Temporal trends of for ASMR for neoplasms, cardiovascular disease and diabetes mellitus attributable to dietary risks between 1990 and 2030 in Global. The dotted lines represent the observational values from the Global Burden of Disease data set. The predictive mean values are shown as black solids, and the fan is the predictive distribution between the 5 and 95% quintiles. **(a)** ASMR for neoplasms; **(b)** ASMR for cardiovascular disease; **(c)** ASMR for diabetes mellitus. ASMR, age-standardized mortality rate.

Figure 5 illustrates the age-standardized mortality trend predictions of chronic diseases attributable to diet, from 2021 to 2030. For dietary-related neoplasms and cardiovascular diseases, the ASMR is projected to decline gradually, with total population percentage changes of −4.71% and −12.33%, respectively. In contrast, the ASMR for diet-related diabetes exhibited a slight upward trend, reflecting a total population percentage change of 1.93% (Supplementary Table S8). Additionally, our findings indicate that the ASMR for women is lower than that for men, with specific values of ASMR trends by age detailed in Supplementary Figure S4. Projections beyond 2021 (dashed vertical line) include 95% credible intervals (shaded bands), reflecting uncertainty from model parameters and future trend variability. Policymakers should note that wider intervals indicate higher uncertainty in long-term estimates.

## 4 Discussion

This study provides a comprehensive overview of the chronic disease burden attributable to dietary risk factors across 204 countries and regions worldwide, from 1990 to 2021, while also projecting trends in diet-related chronic disease deaths by 2030.

From 1990 to 2021, there was a gradual increase in deaths and DALYs despite the gradual decrease in the ASMR and ASDR for diet-related neoplasms and cardiovascular diseases. Although the ASMR for diet-related diseases showed a declining trend, the absolute number of deaths increased during the study period. This discrepancy can be attributed to demographic shifts, including population growth and aging, which lead to larger at-risk populations and prolonged exposure to dietary risk factors over time. Similar patterns have been observed in other global burden studies. In 2021, 1 in 10 people worldwide was aged 65 (25). Although ASMR for dietary diabetes showed a slight decline over the past three decades, ASDR significantly increased by 38.38%, along with a substantial increase in deaths and DALYs of 132.48 and 196.84%, respectively. Although ASMR for neoplasms and cardiovascular diseases is expected to decrease over the next decade, the ASMR for diabetes is expected to slightly increase. Our study projects a greater burden of chronic diseases in men compared to women, over the next decade.

We observed that the burden of chronic dietary disease is greater in men than in women and more pronounced in older individuals compared to younger ones. Men are more likely to consume unhealthy foods, while women tend to have healthier eating patterns (26). Additionally, energy intake varies by sex, age group, and physical activity level, which may significantly influence the differences in disease burden between older and younger adults (27). Our findings indicate that the disparities in burdens may stem from higher neoplasm and cardiovascular mortality rates among men below 70 years of age (28, 29). Therefore, it is particularly important to provide healthy dietary guidance for men and elderly populations. Evidence indicates that the higher cancer and cardiovascular mortality observed in men under 70 stems from biological factors like androgen-mediated lipid metabolism, behavioral patterns including lower fruit/vegetable intake, and healthcare utilization (30, 31). Studies indicate that the Mediterranean diet can reduce the risk of cardiovascular events by 28% in men, with extra virgin olive oil and nuts being especially recommended (32). Additionally, other research suggests that a plant-based dietary pattern with moderate amounts of healthy animal foods may promote overall healthy aging (33).

Temporal trend and dynamic association analysis findings show that global mortality and DALYs for neoplasms, cardiovascular diseases, and diabetes have declined over the past three decades, reflecting the effectiveness of global health policies, and public health interventions and vaccination programs promoted by the WHO (12, 13). However, the increase in deaths and DALYs, particularly in diet-related diabetes, underscores the need to scale up efforts on the improvement of dietary habits and chronic disease management. According to the dynamic association analysis, most deaths and disease burdens from dietary neoplasms and cardiovascular diseases occur in medium-high SDI areas, where diets are characterized by high consumption of red meat and sodium coupled with low intake of whole grains and fruits. Notably, despite increasing health awareness, red meat maintains its dietary dominance in high-SDI regions, likely due to deeply entrenched cultural preferences and comparatively lax policy restrictions relative to other dietary risks like sugar and salt (5). This persistent pattern suggests that targeted policy interventions, such as taxation strategies similar to those implemented for sugary drinks, may be necessary to accelerate reductions in red meat consumption (34, 35). In countries with GDP per capita >$30,000, traditional dietary customs (e.g., barbecue culture in Western countries, red meat as nutritional supplementation in East Asia) maintain average daily red meat intake at 83-112 g significantly exceeding WHO recommendations of 14–28 g. This dietary transition lag appears to follow a 20–30 year cycle relative to nutritional awareness. Currently, 76% of high-SDI nations lack systematic policy frameworks for dietary transition. Comparative policy analysis shows nations implementing comprehensive interventions (e.g., Finland’s combined taxation, education, and alternative protein subsidies) achieved 28% red meat reduction versus just 9% in countries relying solely on public education (36, 37). Targeted measures like taxation (e.g., Denmark’s saturated fat tax) could accelerate reductions. These are the major risk factors. Over time, diet-related risk factors for neoplasms have gradually shifted toward increased red meat consumption. Notably, southern Sub-Saharan Africa has higher diet-related neoplasm and cardiovascular disease mortality rates compared to other regions in Africa. Global reports consistently identify Sub-Saharan Africa as experiencing one of the world’s most rapid urbanization processes, with urban populations growing at 3.5% annually—nearly double the global average (38). This urban transformation has been accompanied by a surge in ultra-processed food sales (39). This nutrition transition mirrors patterns seen in other rapidly urbanizing middle-income countries, where traditional diets are being replaced by Westernized food options. Evidence-based policy interventions could help mitigate this shift, as demonstrated by Mexico’s successful soda tax implementation and Thailand’s innovative mobile market programs that improved access to fresh foods in urban areas (40). Rapid urbanization in middle-income countries drives Westernized diets; lessons from Mexico’s soda tax or Thailand’s mobile markets could mitigate this transition. The high mortality and disease burden from diet-related neoplasms and cardiovascular diseases may also be influenced by social changes and cultural diversity (14, 41). These findings underscore the urgent need for aggressive measures to improve dietary habits, particularly in high and medium SDI areas. Public health policies should focus on educating communities while providing incentives for consumers and producers to support healthy food options.

Addressing the diet-related risk factors is key in the management of diabetes. Although the current diet-related mortality rate for diabetes is on the decline, the incidence of diabetes and diet-related ASDR continues to rise, leading to an increased disease burden. The stable mortality but rising ASDR suggests improved diagnosis prolongs survival, yet inadequate management increases disability. Our findings align with emerging evidence that expanded screening programs in high-SDI countries have increased early detection, while persistent disparities in treatment access - particularly for glycemic control and complication prevention—contribute to growing disability burdens. For instance, regions with universal healthcare coverage showed 23% lower disability progression rates despite similar diagnosis rates (42). Strengthening treatment equity through integrated care models appears critical to breaking this pattern. Our predictions indicate that the mortality rate for diabetes will continue to increase over the next decade. Moreover, the ASMR and ASDR for diabetes are disproportionately higher in low- and moderate-SDI regions, with Oceania exhibiting the most pronounced burden. For instance, Pacific Island nations such as Fiji, Kiribati, and Papua New Guinea face particularly high rates, likely driven by rapid dietary shifts toward imported processed foods (e.g., corned beef, instant noodles), reduced access to fresh produce due to geographic isolation, and urbanization-linked sedentary lifestyles. Additionally, healthcare system constraints—such as limited screening programs and insulin affordability issues—may further exacerbate disparities in these settings. Thus, fiscal measures such as implementing fat taxes on high-fat meat products or offering produce subsidies for fruits and vegetables may serve as effective policy tools to promote healthier dietary choices (43). From agricultural production to retail, the entire food system must form the foundation for healthy diets—a principle that should be embedded in all policies shaping dietary patterns. Demand-side strategies to promote healthier food choices (e.g., nutrition education, social marketing, government procurement programs, fiscal measures like subsidies for nutritious foods and taxes on unhealthy products) can transmit market signals through the value chain, thereby stimulating alternative, sustainable sources of nutritious food (44). Food deserts in urban areas and trade imbalances exacerbate dietary risks, necessitating global trade reforms alongside local supply-chain investments (45). Processed meat is often more affordable and easier to preserve, transport, and prepare, making it a more attractive option for individuals in these regions. In Oceania, promoting indigenous crops and subsidizing local fisheries could counter processed food reliance, while Sub-Saharan Africa may benefit from school-based legume programs to improve affordability (46).

While Western diet—characterized by high consumption of processed meats and sugar-sweetened beverages—are linked to elevated metabolic risks, Mediterranean diets—rich in plant-based foods, olive oil, and fish—have demonstrated protective effects against cardiometabolic diseases (32). This contrast underscores the importance of dietary structure beyond individual nutrients. While our study focused on the independent effects of specific dietary factors, emerging evidence suggests that *nutrient-nutrient interactions* may further modulate chronic disease risks. Cohort studies indicate that high dietary fiber intake (particularly from whole grains) may attenuate the pro-inflammatory and carcinogenic effects of red meat through mechanisms such as short-chain fatty acid production and fecal mutagen binding (47). Similarly, the hypertensive effects of high sodium intake appear to be less pronounced in individuals with diets rich in potassium-containing fruits and vegetables, likely mediated through improved renal sodium handling and endothelial function (48). Moreover, excessive sodium intake could heighten disease susceptibility by exacerbating obesity and fostering systemic inflammation. These findings underscore the complex interplay between dietary components in disease pathogenesis. A large-scale prospective cohort study found that heme iron intake may increase the risk of type 2 diabetes through pathways such as insulin resistance, inflammation, and lipid metabolism. It could also partially explain the association between unprocessed red meat, specific dietary patterns, and the risk of type 2 diabetes (49). Beyond specific nutrients (e.g., heme iron or sodium), growing evidence highlights the role of overall dietary patterns and ultra-processed food consumption in shaping regional disparities in chronic disease risk. Western dietary patterns—characterized by high intake of processed meats, refined grains, and sugar-sweetened beverages—have been linked to elevated risks of cardiovascular disease, type 2 diabetes, and certain neoplasms (50–52). Contrasting sharply with global dietary guidelines such as the EAT-Lancet Commission’s planetary health diet and WHO recommendations, which limit red meat to <98 g/week and emphasize whole grains, legumes, and plant-based fats (35, 53). The high prevalence of ultra-processed foods in modern diets further exacerbates these risks, as their composition often violates multiple guideline thresholds for added sugars, sodium, and saturated fats (54). In contrast, traditional diets such as the Mediterranean, DASH (Dietary Approaches to Stop Hypertension), or Asian plant-based diets, which emphasize whole grains, legumes, fish, and unsaturated fats, demonstrate consistent protective effects against these condition (55). Notably, the EAT-Lancet Commission’s “flexitarian” approach (e.g., 50% plant-based calories) and WHO’s sugar intake targets (<10% total energy) provide actionable frameworks to bridge the gap between current Western diets and optimal dietary patterns (35, 53).

When assessing the impact of dietary patterns on health, it is essential to fully account for the complexity of real-world eating behaviors (56). Individuals typically do not consume isolated single food categories (such as whole grains or vegetables) but rather eat specific combinations of foods (e.g., a typical fast-food meal of “hamburger + French fries + sugar-sweetened beverages”). These food pairings can produce significant synergistic or antagonistic nutritional effects: certain combinations (such as the co-consumption of high-salt and high-fat foods) may exacerbate cardiovascular disease risk, while others (like the pairing of legumes and grains) may enhance protein utilization through complementary amino acid profiles (50, 57). Consequently, analyzing the intake of individual food categories alone may fail to comprehensively reflect true dietary quality and health risks. Future research should more systematically examine the nutritional characteristics and long-term health effects of culturally specific food combinations.

Beyond elucidating dietary risk factors, our findings underscore several actionable public health strategies for chronic disease prevention. First, food reformulation initiatives could simultaneously target multiple risk pathways. Currently, at least 96 countries worldwide have implemented national salt reduction action plans, employing comprehensive strategies including food environment interventions and product reformulation to reduce population-level sodium intake (58). The success of Chile’s front-of-package warning labels (implemented in 2016) suggests that enhanced nutrition labeling - especially when combined with marketing restrictions—can significantly reduce purchases of ultra-processed foods (59). Importantly, our results support integrated interventions that account for nutrient interactions; for example, nutrition education programs might emphasize consuming potassium-rich produce alongside moderate sodium reduction, rather than sodium restriction alone. Such multifaceted approaches align with recent WHO guidelines advocating for ‘policy packages’ that combine educational, economic, and environmental strategies (53). Future implementation should consider regional variations in food availability and cultural dietary patterns to ensure equitable adoption.

As we project in the future, several challenges are foreseen. These include challenges in developing effective strategies for preventing and controlling foodborne chronic diseases, especially in low-resource settings. Furthermore, ensuring equitable access to quality treatment for all individuals is crucial. Achieving this goal will rely on international collaboration and resource sharing. Over the next decade, through global collaboration, we can significantly reduce the burden of chronic diseases and enhance the quality of life of individuals living with these conditions. Governments should implement evidence-based regional dietary guidelines, as exemplified by China’s 2022 Eastern Healthy Diet model which emphasizes plant-based foods and traditional cooking methods. The World Health Organization’s 2021 global review identifies such dual-track strategies combining policy guidance with grassroots delivery as the most effective framework for achieving dietary equity (60).

Although this study primarily focuses on diabetes, cardiovascular diseases, and cancer, emerging evidence from bibliometric analyses indicates that diet-related kidney disease research is becoming a rapidly evolving field (61). Of particular note are the established associations between high-salt diets and IgA nephropathy, as well as high sugar intake and diabetic nephropathy. These findings suggest that future GBD data analyses should incorporate specific kidney disease subtypes to enable a more comprehensive evaluation of the public health implications of dietary interventions.

## 5 Limitations

Our study has several limitations. Firstly, while the current GBD classification does not explicitly link chronic respiratory diseases to dietary risks, emerging evidence—such as the role of anti-inflammatory diets (e.g., high in fruits, vegetables, and omega-3 fatty acids) in reducing airway inflammation—suggests a potential mechanistic pathway (17). This limitation highlights an important avenue for future research. As the GBD framework evolves to incorporate broader determinants of health, we anticipate opportunities to integrate dietary factors into respiratory disease analyses. Secondly, although behavioral factors, such as smoking and alcohol consumption are linked to mortality and DALYs from chronic diseases, the burden of disease attributable to these behavioral factors has not been estimated. Future work should model combined effects such as alcohol-red meat interactions in NCDs. Thirdly, our analysis focused solely on the burden of chronic diseases related to dietary factors; however, the combined effects of diet and other risk factors may influence this burden. Fourthly, the GBD data also contain inherent systematic biases, including heterogeneity in evidence quality and substantial variability in relative risk estimates and exposure data—particularly for socioeconomic factors and conflict-affected regions. For harmful risks with monotonically increasing risk functions, we conventionally set the TMREL at zero. In contrast, determining the TMREL for protective risks (e.g., fruit or whole grain intake) presents greater methodological challenges, as defining optimal exposure thresholds requires more nuanced analysis (21). Fifthly, no standardization was performed for mortality attributable to inadequate medical resources or insufficient healthcare capacity. Sixthly, several limitations should be noted regarding our subgroup analyses. First, as the analysis examined associations across multiple countries, diseases, and dietary factors, the multiple comparisons increased the risk of Type I error, raising the possibility of chance findings. Second, the study may have been underpowered to detect certain interaction effects, particularly in small subgroups where statistical power was further limited. To address these issues, future studies with focused hypotheses could apply stricter multiplicity adjustments to confirm the robustness of findings. Additionally, we attempted to address autocorrelation, residual temporal dependencies may remain. Future studies could incorporate more advanced time-series approaches. Despite these limitations, our study represents the first comprehensive overview of the burden of diet-related chronic diseases from 1990 to 2030, employing the latest data and advanced modeling techniques. Although diet is not recognized as a formal respiratory disease risk factor in GBD studies, emerging research indicates processed foods may worsen systemic inflammation and asthma. Future updates to the GBD methodology could explore including dietary components to more comprehensively evaluate their impact on respiratory health outcomes (17).

## 6 Conclusion

The global burden of chronic diseases attributable to dietary risk factors varies among different disease types. While the standardized burden of diet-related neoplasms and cardiovascular diseases has decreased, the burden of diet-related diabetes rapidly increased, emerging as a significant global health challenge. Given the modifiability of dietary behaviors and their far-reaching impact, we recommend enhancing health education, optimizing the food supply chain, and accelerating the development of precision medicine. These initiatives will provide strong support for achieving the goals of the “2030 Sustainable Development Agenda” and “2020–2030 Global Non-Communicable Diseases Covenant.” Aligning with SDG 3.4, we urge nations to adopt WHO’s 2025 trans-fat elimination targets and monitor progress using dietary risk-specific metrics.

## Data Availability

Publicly available datasets were analyzed in this study. This data can be found at: http://www.healthdata.org/gbd/.

## Footnotes

^1^ http://www.healthdata.org/gbd/
