# Supplementary material for: Trends in the burden of chronic diseases attributable to diet-related risk factors from 1990 to 2021 and the global projections through 2030: a population-based study
Source: Front Nutr. 2025 May 9;12:1570321. doi: 10.3389/fnut.2025.1570321 (PMC12098078; doi:10.3389/fnut.2025.1570321)
Supplement: Supplementary file 1 [file Table_1.docx]

**Title**: Trends in the burden of chronic diseases attributable to dietary risk factors from 1990 to 2021 and global projections through 2030: A population-based study

**Authors**: Huan Ma, Minyan Wang, Chu Qin, Haojie Ni, Yun Shi, Conghua Ji

Catalogue

[Table S1 Mortality and disability-adjusted life years attributable to dietary factors of Neoplasms patients in 204 countries and regions from 1990 to 2021 3](#_Toc169126571)

[Table S2 Mortality and disability-adjusted life years attributable to dietary factors of Cardiovascular disease patients in 204 countries and regions from 1990 to 2021 12](#_Toc169126572)

[Table S3 Mortality and disability-adjusted life years attributable to dietary factors of Diabetes mellitus patients in 204 countries and regions from 1990 to 2021 21](#_Toc169126573)

[Figure S1 Burden of chronic diseases attributed to dietary risks in 204 countries and regions in 2021 30](#_Toc169126574)

[Table S4 Countries and regions with an average annual decline in mortality and disability-adjusted life years attributed to dietary factors for three chronic diseases from 1990 to 2021 31](#_Toc169126575)

[Table S5 Countries and regions with an average annual increasing in mortality and disability-adjusted life years attributed to dietary factors for three chronic diseases from 1990 to 2021 34](#_Toc169126576)

[Table S6 Mortality and disability-adjusted life years attributable to dietary factors among patients with chronic diseases in 5 SDI and 21 GBD regions from 1990 to 2021 35](#_Toc169126577)

[Table S7 SDI and GBD regions with an average annual change in mortality and disability-adjusted life years attributed to dietary factors for three chronic diseases from 1990 to 2021 39](#_Toc169126578)

[Figure S2 Temporal trends in the global burden of chronic diseases attributable to dietary factors from 1990 to 2021 41](#_Toc169126579)

[Figure S3 Temporal trends in the global burden of chronic diseases age-standard population attributable proportion rate to dietary factors from 1990 to 2021 42](#_Toc169126580)

[Table S8 Predict the standardized mortality rates of different chronic diseases from 2021 to 2030 43](#_Toc169126581)

[Figure S4 Temporal trends of for ASMR for neoplams, cardiovascular disease and diabetes mellitus attributable to dietary risks among different gender between 1990 and 2030 in Global 45](#_Toc169126582)

# Table S1 Mortality and disability-adjusted life years attributable to dietary factors of Neoplasms patients in 204 countries and regions from 1990 to 2021

| **Countries or territories** | **ASR of Deaths (per 100,000 population)** | | | **ASR of DALYs (per 100,000 population)** | | |
| --- | --- | --- | --- | --- | --- | --- |
|  | **1990(95%UI)** | **2021(95%UI)** | **Percentage changes (%)** | **1990(95%UI)** | **2021(95%UI)** | **Percentage changes (%)** |
| Global | 12.24(3.32-22.78) | 7.90(2.45-13.85) | -35.46 | 302.48(80.53-565.63) | 189.62(57.13-335.37) | -37.31 |
| Afghanistan | 11.04(2.42-24.83) | 10.55(2.51-22.12) | -4.44 | 301.90(59.80-680.86) | 283.20(61.73-609.37) | -6.19 |
| Albania | 6.50(1.53-14.55) | 4.92(1.02-10.55) | -24.31 | 151.32(34.18-337.58) | 109.90(21.58-236.40) | -27.37 |
| Algeria | 3.22(1.04-5.94) | 2.67(0.70-4.85) | -17.08 | 74.95(22.07-140.44) | 61.97(14.54-115.78) | -17.32 |
| American Samoa | 8.78(3.42-17.3) | 9.62(3.45-18.14) | 9.57 | 223.33(86.47-448.11) | 253.27(87.63-482.03) | 13.41 |
| Andorra | 13.17(2.67-24.32) | 8.42(1.82-14.99) | -36.07 | 307.29(60.10-577.50) | 193.84(40.35-349.33) | -36.92 |
| Angola | 8.96(2.98-15.63) | 7.26(2.46-12.2) | -18.97 | 240.49(69.16-426.22) | 189.51(54.65-336.31) | -21.20 |
| Antigua and Barbuda | 8.43(2.07-16.98) | 8.98(2.49-16.12) | 6.52 | 204.46(45.69-408.63) | 208.71(53.16-376.91) | 2.08 |
| Argentina | 14.38(3.67-25.54) | 11.37(2.86-19.58) | -20.93 | 339.78(82.19-614.69) | 267.84(62.98-466.91) | -21.17 |
| Armenia | 10.29(2.82-20.20) | 7.61(1.95-14.17) | -26.04 | 286.04(75.73-561.94) | 181.94(43.70-341.21) | -36.39 |
| Australia | 13.60(3.20-23.15) | 8.25(1.73-13.91) | -39.34 | 327.42(72.42-558.62) | 193.08(38.43-331.08) | -41.03 |
| Austria | 14.19(3.41-25.47) | 6.94(1.40-12.19) | -51.09 | 325.76(73.51-594.07) | 156.52(29.36-276.37) | -51.95 |
| Azerbaijan | 8.96(1.75-18.85) | 5.44(1.16-12.18) | -39.29 | 249.39(48.95-519.34) | 142.85(30.37-314.92) | -42.72 |
| Bahamas | 10.05(3.13-17.80) | 10.11(3.89-17.62) | 0.60 | 277.58(76.70-501.25) | 270.98(88.04-488.92) | -2.38 |
| Bahrain | 7.86(2.06-14.29) | 5.98(1.54-10.42) | -23.92 | 181.67(45.79-333.43) | 132.17(31.19-234.73) | -27.25 |
| Bangladesh | 5.84(2.53-9.99) | 3.71(1.39-6.44) | -36.47 | 160.91(65.75-275.49) | 96.92(33.01-168.94) | -39.77 |
| Barbados | 12.12(3.47-21.75) | 12.15(3.81-21.23) | 0.25 | 293.82(77.04-536.14) | 285.40(76.35-516.19) | -2.87 |
| Belarus | 12.20(2.89-25.75) | 9.85(2.48-17.45) | -19.26 | 330.08(75.26-701.99) | 248.39(60.26-448.53) | -24.75 |
| Belgium | 16.06(3.96-27.84) | 8.78(1.78-15.02) | -45.33 | 362.02(84.05-631.54) | 198.29(37.95-340.02) | -45.23 |
| Belize | 3.85(1.32-8.56) | 4.40(1.76-8.52) | 14.29 | 97.84(29.14-216.98) | 117.98(41.69-231.69) | 20.58 |
| Benin | 4.18(2.02-8.40) | 3.95(1.82-7.40) | -5.50 | 111.42(48.26-223.45) | 102.58(43.63-191.40) | -7.93 |
| Bermuda | 15.44(4.87-26.74) | 9.51(2.99-16.24) | -38.41 | 353.57(102.12-625.02) | 217.23(61.03-379.51) | -38.56 |
| Bhutan | 4.77(1.30-9.02) | 3.68(0.81-7.03) | -22.85 | 128.22(33.09-248.19) | 92.42(18.93-177.14) | -27.92 |
| Bolivia (Plurinational State of) | 11.96(4.37-30.75) | 10.38(4.07-23.05) | -13.21 | 293.03(96.88-742.13) | 241.58(86.05-538.35) | -17.56 |
| Bosnia and Herzegovina | 9.66(3.17-17.40) | 9.84(2.36-17.57) | 1.86 | 246.87(78.55-440.72) | 229.72(52.41-417.31) | -6.95 |
| Botswana | 11.42(2.59-19.87) | 9.86(2.34-17.14) | -13.66 | 289.92(61.43-506.12) | 239.31(54.23-420.02) | -17.46 |
| Brazil | 8.43(1.74-17.55) | 7.65(1.71-13.79) | -9.25 | 210.35(39.67-438.58) | 197.86(42.37-359.41) | -5.94 |
| Brunei Darussalam | 15.72(4.87-28.77) | 11.27(3.56-19.02) | -28.31 | 381.92(110.51-705.43) | 272.19(79.76-470.10) | -28.73 |
| Bulgaria | 12.70(3.49-24.40) | 14.22(3.75-24.88) | 11.97 | 323.78(86.57-617.24) | 349.38(88.06-616.35) | 7.91 |
| Burkina Faso | 6.37(2.49-12.45) | 7.33(2.39-13.32) | 15.07 | 163.41(55.07-318.60) | 181.18(51.28-333.42) | 10.87 |
| Burundi | 9.51(1.96-16.80) | 6.23(2.32-10.07) | -34.49 | 256.21(42.4-463.46) | 157.61(48.1-261.79) | -38.48 |
| Cabo Verde | 7.39(1.16-18.67) | 9.76(1.65-20.41) | 32.07 | 184.09(23.17-447.56) | 221.60(28.66-466.64) | 20.38 |
| Cambodia | 12.88(6.22-21.32) | 12.14(6.25-19.02) | -5.75 | 345.53(155.63-576.91) | 311.06(148.28-496.46) | -9.98 |
| Cameroon | 5.33(2.26-10.64) | 5.60(2.15-10.47) | 5.07 | 137.22(49.55-274.25) | 143.78(47.94-276.69) | 4.78 |
| Canada | 12.50(3.24-21.28) | 8.26(1.84-13.96) | -33.92 | 299.09(72.6-516.61) | 191.73(39.44-328.04) | -35.90 |
| Central African Republic | 10.51(2.45-18.80) | 9.42(2.56-17.21) | -10.37 | 283.52(58.09-518.88) | 251.84(60.21-463.65) | -11.17 |
| Chad | 4.75(1.68-9.54) | 6.69(2.58-12.71) | 40.84 | 119.59(37.20-240.62) | 167.87(55.16-321.72) | 40.37 |
| Chile | 11.68(2.80-26.26) | 8.54(2.47-16.25) | -26.88 | 267.41(60.80-601.96) | 194.21(53.72-368.34) | -27.37 |
| China | 18.44(3.89-38.01) | 8.66(2.98-17.89) | -53.04 | 469.49(97.07-975.92) | 210.49(70.5-431.87) | -55.17 |
| Colombia | 8.50(2.09-20.96) | 6.56(1.75-13.44) | -22.82 | 204.1(47.85-505.48) | 166.79(41.36-340.74) | -18.28 |
| Comoros | 10.17(2.79-16.92) | 9.47(2.95-15.23) | -6.88 | 269.58(64.99-454.85) | 238.43(64.93-399.81) | -11.56 |
| Congo | 11.40(4.13-19.25) | 8.94(3.57-15.02) | -21.58 | 314.71(94.03-558.99) | 246.59(82.18-424.86) | -21.65 |
| Cook Islands | 6.07(1.39-11.13) | 5.05(0.81-9.28) | -16.8 | 167.62(45.9-304.89) | 138.99(28-270.96) | -17.08 |
| Costa Rica | 8.15(1.83-22.06) | 8.44(2.27-17.20) | 3.56 | 192.80(41.27-509.20) | 211.64(54.21-429.07) | 9.77 |
| Cote d'Ivoire | 0.71(-3.62-3.25) | 0.77(-3.45-3.18) | 8.45 | 40.94(-34.58-87.67) | 43.26(-38.15-89.50) | 5.67 |
| Croatia | 14.44(4.25-27.75) | 11.7(3.31-19.81) | -18.98 | 332.55(93.35-635.72) | 262.39(70.95-440.18) | -21.1 |
| Cuba | 9.15(2.96-15.6) | 9.18(2.68-15.67) | 0.33 | 218.38(65.49-379.44) | 215.13(56.97-366.97) | -1.49 |
| Cyprus | 11.25(2.91-19.81) | 7.62(1.87-12.98) | -32.27 | 227.42(55.79-405.37) | 159.85(34.62-276.39) | -29.71 |
| Czechia | 18.19(4.89-31.69) | 10.63(2.56-18.11) | -41.56 | 431.67(112.76-754.07) | 243.38(55.81-419.50) | -43.62 |
| Democratic People's Republic of Korea | 10.46(3.34-21.96) | 9.92(3.39-20.55) | -5.16 | 276.10(85.03-589.16) | 270.41(89.18-561.71) | -2.06 |
| Democratic Republic of the Congo | 5.43(2.25-9.26) | 5.46(2.62-8.80) | 0.55 | 151.9(50.09-261.83) | 147.11(59.2-245.15) | -3.15 |
| Denmark | 15.74(3.59-27.36) | 10.78(2.02-18.33) | -31.51 | 379.89(82.46-671.31) | 228.54(40.84-392.56) | -39.84 |
| Djibouti | 11.20(3.00-19.27) | 11.50(3.36-20.65) | 2.68 | 286.29(67.49-499.2) | 280.09(73.14-522.78) | -2.17 |
| Dominica | 10.21(1.94-22.01) | 10.26(2.06-20.43) | 0.49 | 234.72(40.72-500.83) | 237.20(43.45-474.72) | 1.06 |
| Dominican Republic | 4.89(1.87-8.65) | 5.22(1.7-9.48) | 6.75 | 119.24(39.58-217.28) | 132.35(38.70-245.78) | 10.99 |
| Ecuador | 6.46(1.46-18.09) | 6.24(1.73-14.61) | -3.41 | 149.18(31.00-416.53) | 148.18(38.32-344.59) | -0.67 |
| Egypt | 3.12(1.35-5.09) | 4.57(1.52-8.29) | 46.47 | 87.03(33.89-147.46) | 120.83(36.46-224.36) | 38.84 |
| El Salvador | 4.63(1.26-12.02) | 5.63(1.42-12.97) | 21.60 | 119.73(31.23-306.97) | 148.33(35.88-337.31) | 23.89 |
| Equatorial Guinea | 9.19(3.03-16.13) | 7.60(2.51-13.65) | -17.30 | 254.56(65.67-460.33) | 200.29(53.16-375.67) | -21.32 |
| Eritrea | 12.68(2.94-22.48) | 11.86(3.42-21.00) | -6.47 | 345.28(71.75-619.54) | 299.96(77.15-541.25) | -13.13 |
| Estonia | 13.38(3.05-25.11) | 10.25(1.96-17.85) | -23.39 | 345.89(77.51-654.79) | 231.51(42.98-407.93) | -33.07 |
| Eswatini | 12.58(2.60-21.62) | 14.31(3.35-25.66) | 13.75 | 318.48(61.07-553.91) | 375.20(80.7-693.36) | 17.81 |
| Ethiopia | 18.90(8.83-29.22) | 11.93(5.03-18.32) | -36.88 | 476.54(214.67-753.26) | 271.15(102.97-425.94) | -43.10 |
| Fiji | 8.42(3.02-14.82) | 8.58(2.45-15.76) | 1.90 | 222.28(72.59-400.75) | 214.94(55.53-406.6) | -3.30 |
| Finland | 10.28(2.41-18.76) | 7.01(1.41-12.05) | -31.81 | 243.51(54.62-449.50) | 160.39(30.20-276.88) | -34.13 |
| France | 15.24(3.07-26.31) | 9.53(1.97-16.16) | -37.47 | 350.35(65.99-607.00) | 221.24(42.60-381.85) | -36.85 |
| Gabon | 10.46(3.69-17.9) | 9.52(3.64-16.13) | -8.99 | 278.10(84.86-482.29) | 246.49(80.47-431.34) | -11.37 |
| Gambia | 2.83(1.34-4.81) | 3.10(1.21-5.03) | 9.54 | 72.19(31.07-125.45) | 79.39(26.94-131.92) | 9.97 |
| Georgia | 8.47(1.97-17.03) | 9.27(2.38-16.97) | 9.45 | 241.56(54.09-482.36) | 238.66(58.92-437.18) | -1.20 |
| Germany | 15.29(3.4-26.55) | 9.16(1.65-15.83) | -40.09 | 352.07(73.6-617.05) | 213.77(36.62-372.73) | -39.28 |
| Ghana | 2.48(-0.98-5.67) | 3.54(0.57-6.69) | 42.74 | 80.81(15.47-160.29) | 97.28(25.13-179.40) | 20.38 |
| Greece | 9.42(2.26-18.07) | 7.90(1.78-13.84) | -16.14 | 211.85(47.35-410.47) | 178.53(37.4-316.20) | -15.73 |
| Greenland | 26.43(6.31-44.99) | 14.57(2.8-24.7) | -44.87 | 648.21(149.77-1108.39) | 351.40(66.56-607.02) | -45.79 |
| Grenada | 7.35(3.09-13.44) | 8.10(3.26-13.06) | 10.20 | 200.24(70.57-365.86) | 201.06(75.7-330.26) | 0.41 |
| Guam | 8.70(3.13-14.89) | 5.63(1.82-9.50) | -35.29 | 199.73(67.7-343.61) | 167.28(49.97-289.81) | -16.25 |
| Guatemala | 5.70(1.75-17.17) | 5.48(1.72-14.63) | -3.86 | 133.46(37.5-394.54) | 137.64(40.22-359.58) | 3.13 |
| Guinea | 2.88(1.24-6.56) | 3.53(1.39-7.46) | 22.57 | 79.68(33.32-174.41) | 97.42(33.92-207.35) | 22.26 |
| Guinea-Bissau | 7.46(2.97-15.5) | 8.43(3.33-15.63) | 13.00 | 201.52(71.7-416.92) | 220.52(72.44-414.74) | 9.43 |
| Guyana | 6.41(2.96-12.13) | 6.58(1.93-12.79) | 2.65 | 166.90(69.01-318.85) | 175.87(46.19-349.89) | 5.37 |
| Haiti | 10.15(4.61-20.32) | 8.24(3.47-15.38) | -18.82 | 271.88(111.31-555.20) | 228.02(97.08-434.51) | -16.13 |
| Honduras | 4.08(1.22-10.58) | 5.64(1.38-14.03) | 38.24 | 107.61(29.82-271.63) | 137.48(30.51-339.44) | 27.76 |
| Hungary | 17.82(4.83-31.73) | 14.15(3.84-23.89) | -20.59 | 431.45(112.33-774.97) | 344.19(89.78-584.15) | -20.22 |
| Iceland | 11.7(2.66-20.57) | 7.49(1.45-12.86) | -35.98 | 279.30(60.15-494.77) | 171.53(31.92-299.04) | -38.59 |
| India | 4.04(1.45-7.62) | 3.97(1.51-6.90) | -1.73 | 110.95(37.89-208.55) | 105.62(37.9-183.06) | -4.80 |
| Indonesia | 7.63(4.29-12.34) | 7.66(4.27-12.19) | 0.39 | 206.49(107.1-338.39) | 194.81(97.43-313.42) | -5.66 |
| Iran (Islamic Republic of) | 5.67(1.48-12.64) | 4.36(1.31-8.57) | -23.10 | 141.54(35.06-318.23) | 107.98(30.17-212.32) | -23.71 |
| Iraq | 3.52(1.34-6.37) | 4.13(1.79-7.06) | 17.33 | 100.81(34.02-187.22) | 108.52(42.67-195.55) | 7.65 |
| Ireland | 16.77(3.58-29.17) | 8.63(1.64-15.10) | -48.54 | 389.70(78.76-686.19) | 195.46(34.54-344.62) | -49.84 |
| Israel | 11.45(2.77-20.36) | 6.95(1.61-12.33) | -39.30 | 258.06(57.62-473.21) | 150.50(32.56-268.75) | -41.68 |
| Italy | 12.31(2.92-23.31) | 8.10(1.80-14.17) | -34.20 | 290.85(64.94-547.58) | 184.32(38.08-322.19) | -36.63 |
| Jamaica | 7.43(1.98-14.82) | 8.70(2.13-16.37) | 17.09 | 176.10(42.85-349.37) | 219.98(49.57-420.92) | 24.92 |
| Japan | 12.41(3.44-27.11) | 9.12(2.70-16.61) | -26.51 | 297.55(77.24-645.66) | 212.32(61.1-381.83) | -28.64 |
| Jordan | 5.97(2.16-10.37) | 4.77(1.46-8.37) | -20.10 | 157.82(52.10-274.67) | 120.12(32.88-215.40) | -23.89 |
| Kazakhstan | 18.41(3.87-35.06) | 6.70(1.65-12.75) | -63.61 | 499.55(109.34-945.38) | 171.37(41.07-329.85) | -65.70 |
| Kenya | 5.45(0.54-10.34) | 7.47(0.75-13.62) | 37.06 | 141.96(13.08-273.79) | 186.25(16.44-342.27) | 31.20 |
| Kiribati | 10.11(3.72-20.42) | 10.31(3.61-20.55) | 1.98 | 268.53(89.94-545.77) | 269.27(85.79-539.53) | 0.28 |
| Kuwait | 3.47(1.05-6.19) | 3.88(1.18-6.68) | 11.82 | 84.80(24.01-153.64) | 92.68(25.65-161.40) | 9.29 |
| Kyrgyzstan | 11.62(2.63-24.75) | 5.55(1.23-12.18) | -52.24 | 326.67(73.94-697.50) | 147.74(31.23-329.84) | -54.77 |
| Lao People's Democratic Republic | 12.17(5.37-21.46) | 8.85(4.31-14.37) | -27.28 | 332.65(133.03-595.17) | 231.34(100.27-385.31) | -30.46 |
| Latvia | 13.13(3.03-25.73) | 10.83(2.43-19.15) | -17.52 | 343.55(77.67-680.42) | 260.81(56.43-464.81) | -24.08 |
| Lebanon | 6.63(1.88-12.17) | 5.63(1.56-9.88) | -15.08 | 162.45(41.47-305.02) | 135.64(33.39-244.98) | -16.50 |
| Lesotho | 9.09(3.11-14.87) | 16.69(5.28-28) | 83.61 | 228.24(69.03-382.2) | 439.34(129.18-748.79) | 92.49 |
| Liberia | 3.56(1.64-7.39) | 4.05(1.62-7.72) | 13.76 | 95.99(44.32-200.12) | 112.9(46.33-218.58) | 17.62 |
| Libya | 6.36(2.15-11.11) | 6.9(2.48-12.13) | 8.49 | 157.39(50.29-277.25) | 173.22(58.21-305.96) | 10.06 |
| Lithuania | 12.37(3-24.09) | 10.68(2.28-18.88) | -13.66 | 320.39(74.79-633.02) | 255.38(51.66-455.87) | -20.29 |
| Luxembourg | 16.37(3.3-28.31) | 8.73(1.58-14.77) | -46.67 | 379.36(69.57-664.91) | 190.18(32.4-323.53) | -49.87 |
| Madagascar | 9.43(1.59-16.73) | 8.52(1.98-14.95) | -9.65 | 248.59(36.91-448.69) | 217.17(44.11-396.2) | -12.64 |
| Malawi | 7.75(0.75-12.96) | 9.72(0.67-16.66) | 25.42 | 206.89(12.76-346.59) | 257.25(10.79-451.11) | 24.34 |
| Malaysia | 10.69(5.19-16.63) | 10.14(4.48-16.02) | -5.14 | 273.27(122.2-437.23) | 253.39(97.57-412.42) | -7.27 |
| Maldives | 4.56(2.48-8.06) | 2.05(1.02-3.38) | -55.04 | 120.57(55.6-220.01) | 49.11(20.36-83.28) | -59.27 |
| Mali | 6.71(1.94-15.63) | 5.98(1.29-13.12) | -10.88 | 171.34(45.02-401.67) | 150.35(28.35-327.57) | -12.25 |
| Malta | 12.69(2.89-22.61) | 8.03(1.63-13.49) | -36.72 | 294.1(63.05-523.32) | 183.64(34.74-315.21) | -37.56 |
| Marshall Islands | 10.02(4.46-19.23) | 9.52(4.27-17.73) | -4.99 | 260.22(104.79-503.12) | 261.46(106.54-494.24) | 0.48 |
| Mauritania | 6.73(1.52-13.19) | 7.28(1.74-13.66) | 8.17 | 165.87(35.69-325.88) | 171.99(37.44-325.97) | 3.69 |
| Mauritius | 7.89(2.84-15.06) | 8.17(2.88-14.27) | 3.55 | 200.05(67.48-382.63) | 216.87(67.03-386.48) | 8.41 |
| Mexico | 5.19(1.27-12.01) | 5.19(1.35-10.20) | 0.00 | 121.56(27.03-277.25) | 136.62(32.49-265.17) | 12.39 |
| Micronesia (Federated States of) | 11.03(4.73-21.12) | 10.49(4.49-19.55) | -4.90 | 291.02(112.54-572.08) | 278.06(111.49-529.51) | -4.45 |
| Monaco | 13.95(2.99-24.87) | 14.00(2.46-24.49) | 0.36 | 328.06(67.22-589.89) | 328.14(52.28-582.03) | 0.02 |
| Mongolia | 22.17(4.49-45.63) | 15.07(3.11-31.6) | -32.03 | 559.96(118.97-1159.9) | 366.93(79.85-772.85) | -34.47 |
| Montenegro | 8.48(1.95-15.21) | 9.88(2.17-17.27) | 16.51 | 215.28(47.22-389.85) | 223.73(45.33-390.83) | 3.93 |
| Morocco | 3.58(1.58-5.67) | 4.15(1.44-7.12) | 15.92 | 93.18(38.32-150.91) | 106.19(33.48-186.6) | 13.96 |
| Mozambique | 5.49(1.60-9.66) | 6.68(1.54-11.87) | 21.68 | 129.01(31.72-225.33) | 159.1(31.13-289.51) | 23.32 |
| Myanmar | 11.85(6.34-20.34) | 8.62(3.76-14.45) | -27.26 | 323.28(157.3-561.33) | 224.69(89.63-383.58) | -30.50 |
| Namibia | 5.38(1.89-8.82) | 6.35(1.87-11.68) | 18.03 | 143.74(46.14-240.22) | 171.46(44.68-327.35) | 19.28 |
| Nauru | 12.27(4.02-24.65) | 12.55(4.45-23.71) | 2.28 | 331.56(94.02-666.95) | 339.51(107.75-644.12) | 2.40 |
| Nepal | 4.92(1.27-9.20) | 4.04(0.86-7.8) | -17.89 | 133.47(31.27-251.81) | 104.81(20.81-202.19) | -21.47 |
| Netherlands | 14.76(3.15-25.76) | 11.54(2.11-19.69) | -21.82 | 347.35(68.51-612.87) | 264.36(46.26-450.73) | -23.89 |
| New Zealand | 16.92(4.13-28.79) | 10.22(2.66-16.75) | -39.60 | 410.51(93.27-699.72) | 233.10(57.48-390.11) | -43.22 |
| Nicaragua | 3.94(1.49-9.23) | 4.09(1.45-8.45) | 3.81 | 99.64(34.65-229.92) | 104.11(34.85-212.29) | 4.49 |
| Niger | 4.93(1.64-10.36) | 4.72(1.34-10.03) | -4.26 | 122.82(36.84-257.22) | 113.36(27.32-244.74) | -7.70 |
| Nigeria | 1.72(-2.00-3.94) | 2.15(-2.9-4.94) | 25.00 | 60.51(-6.28-104.74) | 80.50(-10.55-150.48) | 33.04 |
| Niue | 8.96(3.78-16.37) | 8.92(3.14-16.49) | -0.45 | 223.50(83.52-418.68) | 224.37(69.67-426.21) | 0.39 |
| North Macedonia | 10.36(2.89-20.73) | 10.95(2.94-20.30) | 5.69 | 259.89(68.07-517.67) | 241.83(62.01-448.40) | -6.95 |
| Northern Mariana Islands | 9.48(3.26-18.58) | 9.58(3.3-16.91) | 1.05 | 230.21(72.74-456.08) | 232.31(71.44-417.90) | 0.91 |
| Norway | 13.69(2.84-23.28) | 9.80(1.86-16.13) | -28.41 | 316.94(61.59-546.58) | 208.61(37.3-345.85) | -34.18 |
| Oman | 2.87(0.72-6.66) | 2.16(0.49-4.34) | -24.74 | 71.72(17.33-169.67) | 49.96(10.49-101.27) | -30.34 |
| Pakistan | 6.09(1.18-10.90) | 7.15(1.31-13.17) | 17.41 | 161.44(27.57-293.88) | 190.09(30.26-359.27) | 17.75 |
| Palau | 12.64(4.77-25.16) | 10.90(3.71-20.19) | -13.77 | 308.42(106.83-625.51) | 254.76(73.85-482.64) | -17.40 |
| Palestine | 10.85(5.45-17.27) | 8.80(4.38-13.27) | -18.89 | 264.86(120.36-438.28) | 209.55(97.24-329.07) | -20.88 |
| Panama | 5.74(2.52-11.24) | 5.55(2.07-10.66) | -3.31 | 142.40(57.1-276.88) | 145.07(48.94-276.34) | 1.87 |
| Papua New Guinea | 4.72(1.72-11.72) | 3.97(1.24-9.42) | -15.89 | 135.81(42.45-326.30) | 119.71(38.81-268.43) | -11.85 |
| Paraguay | 5.01(1.17-10.07) | 7.07(1.61-13.19) | 41.12 | 124.05(26.66-250) | 173.33(35.8-327.53) | 39.73 |
| Peru | 6.79(2.43-17.11) | 5.72(1.77-14.14) | -15.76 | 168.17(54.63-429.73) | 138.11(37.82-334.22) | -17.87 |
| Philippines | 6.76(3.89-9.77) | 8.36(4.61-12.26) | 23.67 | 188.87(105.5-277.16) | 232.47(114.81-351.92) | 23.08 |
| Poland | 14.24(4.37-25.77) | 13.13(3.76-22.16) | -7.79 | 352.65(107.23-640.86) | 293.46(80.56-499.69) | -16.78 |
| Portugal | 13.88(3.65-27.02) | 9.24(2.27-16.31) | -33.43 | 319.51(77.03-631.93) | 216.40(51.22-386.28) | -32.27 |
| Puerto Rico | 9.26(2.64-16.32) | 6.87(1.79-11.83) | -25.81 | 226.18(58.86-400.71) | 177.32(43.62-309.66) | -21.60 |
| Qatar | 7.68(2.11-14.38) | 5.44(1.35-9.59) | -29.17 | 168.06(44.35-317.66) | 116.83(27.9-210.69) | -30.48 |
| Republic of Korea | 11.5(3.20-30.99) | 7.07(2.22-13.26) | -38.52 | 285.13(73.16-802.68) | 154.61(45.51-293.97) | -45.78 |
| Republic of Moldova | 11.62(2.97-22.03) | 9.93(2.63-17.15) | -14.54 | 309.29(75.16-593.70) | 257.97(65.85-449.37) | -16.59 |
| Romania | 8.19(2.06-16.56) | 11.12(2.57-20.23) | 35.78 | 222.77(54.72-444.07) | 276.32(62-502.5) | 24.04 |
| Russian Federation | 14.52(3.69-29.09) | 11.54(3.06-20.43) | -20.52 | 387.84(96.35-784.69) | 278.91(70.64-498.98) | -28.09 |
| Rwanda | 11.75(1.93-20.03) | 7.98(1.51-14.16) | -32.09 | 318.32(42.87-555.41) | 196.39(30.89-353.63) | -38.30 |
| Saint Kitts and Nevis | 12.14(4.19-21.46) | 9.23(3.69-15.63) | -23.97 | 307.68(91.9-550.73) | 217.68(73.52-379.86) | -29.25 |
| Saint Lucia | 7.62(1.98-16.02) | 5.53(1.62-10.20) | -27.43 | 198.40(59.80-411.40) | 150.01(44.01-278.90) | -24.39 |
| Saint Vincent and the Grenadines | 7.68(2.87-14.81) | 6.39(1.96-11.31) | -16.80 | 194.38(73.79-375.65) | 173.72(57.48-317.19) | -10.63 |
| Samoa | 6.48(2.69-11.85) | 6.20(2.38-11.47) | -4.32 | 159.30(58.41-291.49) | 157.21(52.85-294.78) | -1.31 |
| San Marino | 13.84(3.18-27.95) | 6.35(1.52-12.68) | -54.12 | 298.80(67.34-595.64) | 144.39(31.90-298.68) | -51.68 |
| Sao Tome and Principe | 5.42(3.14-11.06) | 6.37(3.51-12.07) | 17.53 | 127.26(66.00-260.80) | 149.87(69.13-286.60) | 17.77 |
| Saudi Arabia | 3.30(0.92-6.37) | 3.83(1.07-6.54) | 16.06 | 84.07(21.75-165.96) | 100.30(25.87-175.67) | 19.31 |
| Senegal | 5.32(2.5-10.18) | 5.79(2.51-10.76) | 8.83 | 136.91(54.75-267.29) | 145.60(54.39-271.96) | 6.35 |
| Serbia | 14.11(3.91-24.7) | 11.58(2.76-20.19) | -17.93 | 332.34(86.34-586.69) | 275.91(61.80-485.23) | -16.98 |
| Seychelles | 7.95(4.39-13.07) | 7.43(3.52-10.73) | -6.54 | 218.68(101.41-355.57) | 199.33(94.42-297.38) | -8.85 |
| Sierra Leone | 3.45(1.67-7.33) | 3.98(1.90-7.51) | 15.36 | 89.95(43.87-191.03) | 107.08(47.64-196.89) | 19.04 |
| Singapore | 12.10(3.79-21.64) | 6.11(1.87-10.41) | -49.5 | 292.24(88.40-528.35) | 139.65(38.54-242.36) | -52.21 |
| Slovakia | 16.23(4.83-28.41) | 13.54(3.51-22.87) | -16.57 | 412.20(119.66-722.79) | 319.64(79.95-538.61) | -22.46 |
| Slovenia | 13.35(2.99-25.2) | 9.05(2.07-16.58) | -32.21 | 323.16(70-613.01) | 196.33(42.55-355.23) | -39.25 |
| Solomon Islands | 7.22(3.27-16.2) | 6.74(2.71-13.77) | -6.65 | 199.10(81.47-450.61) | 195.03(74.64-401.72) | -2.04 |
| Somalia | 12.76(2.56-22.72) | 11.60(3.61-19.87) | -9.09 | 342.84(59.87-618.64) | 298.83(84.87-529.74) | -12.84 |
| South Africa | 9.55(2.61-15.56) | 11.15(3.21-18.36) | 16.75 | 255.31(62.22-427.13) | 279.88(74.94-460.73) | 9.62 |
| South Sudan | 10.00(1.60-18.47) | 10.20(2.33-18.16) | 2.00 | 257.38(34.16-480.71) | 258.00(50.79-470.33) | 0.24 |
| Spain | 11.69(2.70-20.39) | 8.68(2.02-14.73) | -25.75 | 278.23(59.86-492.43) | 199.80(43.79-342.80) | -28.19 |
| Sri Lanka | 4.84(1.72-8.48) | 3.41(1.17-6.01) | -29.55 | 120.30(39.27-210.84) | 86.31(26.74-157.62) | -28.25 |
| Sudan | 5.46(1.55-12.08) | 5.12(1.5-10.74) | -6.23 | 144.68(37.41-326.12) | 132.50(35.02-284.01) | -8.42 |
| Suriname | 6.88(2.65-12.25) | 6.74(2.93-11.25) | -2.03 | 174.14(59.96-309.99) | 174.59(67.54-299.65) | 0.26 |
| Sweden | 11.05(2.40-19.34) | 7.97(1.62-13.55) | -27.87 | 256.91(51.66-449.12) | 169.86(32.85-292.28) | -33.88 |
| Switzerland | 10.10(2.08-18.12) | 6.45(1.27-11.07) | -36.14 | 241.85(47.31-434.23) | 144.07(27.16-248.17) | -40.43 |
| Syrian Arab Republic | 3.72(1.10-7.11) | 3.70(1.15-7.01) | -0.54 | 100.11(27.53-195.57) | 94.96(26.79-184.68) | -5.14 |
| Taiwan (Province of China) | 10.69(2.88-20.23) | 11.11(3.34-18.51) | 3.93 | 273.35(69.10-517.49) | 270.22(73.74-451.43) | -1.15 |
| Tajikistan | 9.34(2.05-20.54) | 5.04(1.24-10.76) | -46.04 | 253.35(57.31-562.88) | 130.94(32.6-277.78) | -48.32 |
| Thailand | 9.97(5.09-15.59) | 9.09(3.70-15.24) | -8.83 | 254.08(121.24-411.16) | 243.94(88.25-424.85) | -3.99 |
| Timor-Leste | 7.95(4.14-13.4) | 8.29(4.24-12.88) | 4.28 | 205.74(99.98-350.86) | 211.19(100.13-336.34) | 2.65 |
| Togo | 4.87(2.56-9.23) | 6.28(3.09-11.13) | 28.95 | 130(63.97-246.20) | 163.80(72.74-296.26) | 26.00 |
| Tokelau | 9.01(3.99-16.91) | 7.61(2.92-13.47) | -15.54 | 234.55(92.77-440.83) | 203.64(70.37-375.39) | -13.18 |
| Tonga | 7.59(2.59-16.05) | 7.44(2.23-14.58) | -1.98 | 203.66(67.01-422.16) | 196.02(56.98-390.62) | -3.75 |
| Trinidad and Tobago | 7.10(2.51-11.96) | 5.93(2.04-9.43) | -16.48 | 183.82(70.41-323.39) | 171.19(58.98-291.83) | -6.87 |
| Tunisia | 4.39(1.47-7.58) | 3.92(1.02-7.31) | -10.71 | 106.41(33.4-188.79) | 98.23(23.47-187.62) | -7.69 |
| Turkey | 7.74(2.45-15.74) | 5.95(1.66-11.19) | -23.13 | 197.19(58.16-407.54) | 146.04(37.72-278.55) | -25.94 |
| Turkmenistan | 11.21(0.52-21.95) | 4.17(0.83-8.92) | -62.80 | 295.96(16.2-583.23) | 116.92(22.58-251.20) | -60.49 |
| Tuvalu | 10.37(4.5-20.28) | 8.79(3.97-16.04) | -15.24 | 272.21(106.25-538.04) | 230.90(91.66-426.14) | -15.18 |
| Uganda | 9.16(2.01-15.64) | 10.16(1.66-18.37) | 10.92 | 234.20(42.99-410.12) | 259.40(35.53-480.08) | 10.76 |
| Ukraine | 13.76(4.17-27.28) | 9.11(2.41-17.34) | -33.79 | 377.50(110.16-757.13) | 242.13(62.41-465.90) | -35.86 |
| United Arab Emirates | 8.32(2.06-16.37) | 6.79(1.77-12.08) | -18.39 | 205.79(47.58-406.77) | 142.82(34.74-254.60) | -30.60 |
| United Kingdom | 17.32(4.09-29.56) | 10.57(2.10-17.97) | -38.97 | 401.18(89.48-692.10) | 233.49(43.28-399.77) | -41.80 |
| United Republic of Tanzania | 10.11(2.35-17.03) | 8.87(2.11-15.37) | -12.27 | 260.10(55.33-439.31) | 218.73(44.93-389.35) | -15.91 |
| United States of America | 12.81(2.93-21.53) | 8.02(1.58-13.52) | -37.39 | 314.62(67.52-538.20) | 201.95(37.03-345.63) | -35.81 |
| United States Virgin Islands | 11.38(3.66-19.55) | 7.26(1.97-13.15) | -36.2 | 273.92(78.83-476.41) | 183.04(43.58-341.82) | -33.18 |
| Uruguay | 18.10(4.57-31.53) | 16.07(3.66-27.75) | -11.22 | 430.16(102.87-752.72) | 365.76(79.10-636.98) | -14.97 |
| Uzbekistan | 7.59(1.35-15.9) | 3.50(0.76-7.36) | -53.89 | 210.56(37.85-441.67) | 99.53(20.11-208.31) | -52.73 |
| Vanuatu | 8.07(3.76-16.3) | 7.94(3.48-15.00) | -1.61 | 208.06(90.88-418.65) | 209.80(78.92-411.98) | 0.84 |
| Venezuela (Bolivarian Republic of) | 6.39(2.11-14.29) | 6.11(2.08-11.8) | -4.38 | 162.09(47.69-355.13) | 163.69(50.97-316.27) | 0.99 |
| Viet Nam | 7.84(3.61-14.78) | 8.25(3.94-13.25) | 5.23 | 208.04(91.46-395.19) | 210.01(93.62-344.17) | 0.95 |
| Yemen | 6.34(2.04-14.77) | 5.92(2.54-13.17) | -6.62 | 166.6(50.04-391.86) | 152.08(58.77-340.52) | -8.72 |
| Zambia | 12.78(4.35-20.72) | 14.08(5.94-24.40) | 10.17 | 341.08(103.41-554.39) | 372.15(138.98-677.74) | 9.11 |
| Zimbabwe | 12.44(4.66-19.66) | 15.96(5.93-25.52) | 28.30 | 303.26(104.27-496.91) | 418.66(143.09-700.07) | 38.05 |

Note: ASR: age-standardized rates; DALY, disability-adjusted life years; UI, uncertainty interval

# Table S2 Mortality and disability-adjusted life years attributable to dietary factors of Cardiovascular disease patients in 204 countries and regions from 1990 to 2021

| **Countries or territories** | **ASR of Deaths (per 100,000 population)** | | | **ASR of DALYs (per 100,000 population)** | | |
| --- | --- | --- | --- | --- | --- | --- |
|  | **1990(95%UI)** | **2021(95%UI)** | **Percentage changes (%)** | **1990(95%UI)** | **2021(95%UI)** | **Percentage changes (%)** |
| Global | 113.61(31.19-164.63) | 69.81(16.19-104.09) | -38.55 | 2487.47(675.52-3480.84) | 1563.86(378.95-2246.75) | -37.13 |
| Afghanistan | 322.07(70.70-481.14) | 240.75(53.43-362.41) | -25.25 | 7733.46(1473.18-11419.65) | 5572.81(995.75-8294.18) | -27.94 |
| Albania | 156.02(72.57-214.66) | 112.02(46.82-165.48) | -28.20 | 2946.20(1392.98-4011.77) | 1871.37(776.88-2711.3) | -36.48 |
| Algeria | 199.25(39.15-303.55) | 121.34(25.00-191.92) | -39.10 | 3840.27(717.42-5710.24) | 2150.87(422.20-3338.27) | -43.99 |
| American Samoa | 84.28(14.29-134.30) | 84.84(2.84-140.17) | 0.66 | 2046.60(167.05-3181.77) | 2076.47(-74.76-3351.54) | 1.46 |
| Andorra | 41.88(7.06-70.96) | 20.06(3.87-33.51) | -52.10 | 826.78(125.65-1378.76) | 377.31(67.27-600.52) | -54.36 |
| Angola | 145.64(63.47-210.8) | 99.11(37.94-149.42) | -31.95 | 3294.13(1365.56-4779.15) | 2026.16(695.03-3062.68) | -38.49 |
| Antigua and Barbuda | 85.87(42.81-121.36) | 61.84(37.05-85.35) | -27.98 | 1792.18(925.82-2472.24) | 1168.17(690.92-1595.42) | -34.82 |
| Argentina | 109.06(33.04-151.16) | 43.39(15.88-60.56) | -60.21 | 2197.65(714.15-2917.89) | 853.15(285.31-1161.27) | -61.18 |
| Armenia | 199.53(32.96-291.11) | 119.95(7.40-185.06) | -39.88 | 3904.18(503.36-5580.27) | 2305.50(62.70-3477.21) | -40.95 |
| Australia | 77.85(-13.49-132.23) | 19.35(-1.57-31.97) | -75.14 | 1523.71(-282.71-2462.01) | 379.60(-32.07-591.97) | -75.09 |
| Austria | 83.44(-2.77-133.21) | 34.55(3.40-55.04) | -58.59 | 1603.73(-93.92-2510.18) | 595.65(36.87-932.26) | -62.86 |
| Azerbaijan | 238.94(34.67-345.35) | 172.15(5.12-269.83) | -27.95 | 5009.41(578.35-7093.81) | 3264.94(50.99-5039.65) | -34.82 |
| Bahamas | 97.96(54.63-133.51) | 74.26(42.98-102.99) | -24.19 | 2279.45(1258.48-3008.93) | 1630.44(966.24-2258.99) | -28.47 |
| Bahrain | 175.81(1.85-292.65) | 64.73(5.67-108.42) | -63.18 | 3559.18(-38.05-5739.52) | 1213.19(90.46-1974.29) | -65.91 |
| Bangladesh | 128.79(30.85-191.98) | 89.69(18.79-143.14) | -30.36 | 3274.42(663.37-4846.49) | 2008.01(299.23-3225.03) | -38.68 |
| Barbados | 66.76(20.17-100.15) | 37.69(12.35-60.43) | -43.54 | 1344.19(384.56-1946.43) | 750.56(252.99-1177.33) | -44.16 |
| Belarus | 191.89(-2.90-292.58) | 172.17(-14.13-282.72) | -10.28 | 3913.33(-7.42-5629.66) | 3448.72(-292.48-5498.04) | -11.87 |
| Belgium | 67.90(12.94-105.35) | 18.36(4.56-28.47) | -72.96 | 1336.38(266.61-1994.27) | 366.44(91.95-549.29) | -72.58 |
| Belize | 60.93(11.97-96.73) | 40.93(18.16-61.71) | -32.82 | 1346.80(220.48-2101.55) | 879.99(378.41-1300.8) | -34.66 |
| Benin | 73.78(17.05-117.20) | 62.62(14.68-100.01) | -15.13 | 1669.77(378.99-2604.61) | 1355.93(293.56-2148.15) | -18.80 |
| Bermuda | 98.04(9.48-158.63) | 33.65(6.83-54.00) | -65.68 | 1977.18(154.30-3111.51) | 682.66(126.58-1059.65) | -65.47 |
| Bhutan | 90.70(17.16-143.54) | 71.01(11.11-114.29) | -21.71 | 2170.42(343.56-3349.72) | 1525.61(176.29-2470.04) | -29.71 |
| Bolivia (Plurinational State of) | 94.07(33.80-141.44) | 52.85(20.39-83.80) | -43.82 | 1982.29(718.54-2969.63) | 1059.49(414.95-1689.55) | -46.55 |
| Bosnia and Herzegovina | 160.68(61.35-226.85) | 95.33(28.61-148.17) | -40.67 | 3354.93(1165.08-4675.61) | 1773.77(527.68-2733.71) | -47.13 |
| Botswana | 122.15(52.52-183.20) | 79.2(34.92-114.45) | -35.16 | 2677.64(1154.18-3945.19) | 1664.09(683.37-2422.19) | -37.85 |
| Brazil | 87.20(43.84-123.94) | 34.73(18.19-49.31) | -60.17 | 1983.88(1067.11-2719.82) | 806.91(432.44-1107.34) | -59.33 |
| Brunei Darussalam | 100.34(28.79-156.62) | 56.79(15.57-88.86) | -43.40 | 2178.93(547.47-3335.05) | 1224.12(247.64-1892.10) | -43.82 |
| Bulgaria | 302.99(138.03-417.79) | 205.53(112.44-282.34) | -32.17 | 5469.85(2552.68-7343.74) | 3885.53(1997.36-5301.71) | -28.96 |
| Burkina Faso | 91.51(32.29-134.96) | 96.28(32.05-142.67) | 5.21 | 2096.80(737.99-3035.9) | 2098.64(679.41-3056.60) | 0.09 |
| Burundi | 145.33(55.13-224.62) | 91.97(31.12-139.01) | -36.72 | 3381.92(1161.46-5208.57) | 2036.88(646.62-3076.83) | -39.77 |
| Cabo Verde | 58.30(16.07-88.3) | 60.69(9.79-101.4) | 4.10 | 1375.56(392.52-2055.26) | 1244.11(212.46-2036.67) | -9.56 |
| Cambodia | 155.21(60.05-226.77) | 108.86(35.99-168.62) | -29.86 | 3691.03(1342.12-5315.14) | 2364.53(671.56-3681.46) | -35.94 |
| Cameroon | 66.74(19.22-103.63) | 61.79(7.27-109.07) | -7.42 | 1472.20(389.85-2242.79) | 1355.60(123.35-2369.48) | -7.92 |
| Canada | 67.46(-6.81-113.05) | 20.71(-0.75-34.86) | -69.30 | 1360.93(-140.36-2186.77) | 437.91(-6.69-695.54) | -67.82 |
| Central African Republic | 162.66(45.66-240.43) | 146.34(46.06-223.12) | -10.03 | 3715.45(909.36-5531.51) | 3275.31(974.8-4975.32) | -11.85 |
| Chad | 101.77(31.21-152.50) | 112.36(34.48-171.89) | 10.41 | 2358.66(724.53-3438.16) | 2595.63(805.66-3933.34) | 10.05 |
| Chile | 79.97(23.65-116.12) | 27.81(10.62-40.28) | -65.22 | 1542.42(469.66-2173.13) | 587.26(214.28-821.36) | -61.93 |
| China | 128.62(69.7-181.22) | 77.76(30.45-121.22) | -39.54 | 2630.84(1471.93-3614.8) | 1499.58(632.89-2247.22) | -43.00 |
| Colombia | 99.47(34.09-141.55) | 43.27(12.70-65.85) | -56.50 | 2095.12(646.81-2922.67) | 880.84(264.17-1300.89) | -57.96 |
| Comoros | 116.72(53.5-176.88) | 81.57(29.51-124.98) | -30.11 | 2611.90(1088.88-3954.95) | 1753.79(578.20-2693.73) | -32.85 |
| Congo | 162.51(42.74-247.13) | 123.20(38.01-193.69) | -24.19 | 3663.31(790.46-5603.78) | 2628.55(653.92-4138.41) | -28.25 |
| Cook Islands | 179.77(101.05-240.24) | 90.42(44.30-130.60) | -49.70 | 3967.01(2063.50-5299.70) | 2037.31(908.20-2917.07) | -48.64 |
| Costa Rica | 72.73(17.3-108.94) | 31.64(10.95-47.08) | -56.50 | 1491.07(364.62-2174.96) | 697.62(233.00-1020.36) | -53.21 |
| Cote d'Ivoire | 85.02(4.59-140.89) | 81.31(8.81-139.14) | -4.36 | 1911.89(73.61-3121.62) | 1805.22(172.89-3071.54) | -5.58 |
| Croatia | 209.86(89.32-295.59) | 81.46(30.97-119.54) | -61.18 | 3783.89(1564.86-5207.77) | 1395.4(527.61-2003.08) | -63.12 |
| Cuba | 95.46(4.73-153.95) | 47.85(9.00-75.87) | -49.87 | 1939.30(95.93-3030.38) | 973.22(154.19-1505.39) | -49.82 |
| Cyprus | 127.91(38.08-197.09) | 44.12(12.44-67.40) | -65.51 | 2011.24(666.49-2989.77) | 727.03(191.93-1093.91) | -63.85 |
| Czechia | 200.84(51.61-288.59) | 75.12(18.58-113.79) | -62.60 | 4108.52(854.90-5779.84) | 1342.19(308.08-1997.85) | -67.33 |
| Democratic People's Republic of Korea | 119.85(59.86-182.17) | 117.61(52.69-172.71) | -1.87 | 2634.49(1337.58-3970.76) | 2632.27(1147.08-3883.63) | -0.08 |
| Democratic Republic of the Congo | 107.43(15.75-174.89) | 113.92(29.86-177.80) | 6.04 | 2391.37(278.81-3888.59) | 2419.18(543.55-3792.01) | 1.16 |
| Denmark | 98.15(-2.29-159.14) | 19.72(1.19-31.69) | -79.91 | 1925.89(-19.35-2985.26) | 383.71(23.20-593.09) | -80.08 |
| Djibouti | 127.74(66.81-179.27) | 101.07(42.70-156.05) | -20.88 | 2785.15(1386.96-3885.43) | 2164.27(878.76-3370.20) | -22.29 |
| Dominica | 101.90(31.04-154.42) | 67.16(26.06-101.73) | -34.09 | 1904.15(537.45-2838.90) | 1291.07(447.07-1915.00) | -32.20 |
| Dominican Republic | 79.54(30.74-119.38) | 57.27(27.14-88.05) | -28.00 | 1696.71(668.74-2471.33) | 1309.73(643.90-1982.73) | -22.81 |
| Ecuador | 77.88(31.84-108.63) | 49.75(18.12-74.49) | -36.12 | 1573.00(621.23-2154.89) | 948.99(344.21-1405.25) | -39.67 |
| Egypt | 229.02(8.33-352.07) | 145.70(-23.30-261.80) | -36.38 | 4782.33(-56.69-7277.73) | 3112.4(-611.88-5559.45) | -34.92 |
| El Salvador | 67.94(6.68-107.04) | 45.13(3.29-78.59) | -33.57 | 1566.62(84.92-2427.43) | 996.53(36.27-1692.25) | -36.39 |
| Equatorial Guinea | 159.91(47.17-244.13) | 78.65(15.56-140.30) | -50.82 | 3648.26(985.51-5616.65) | 1608.69(208.23-2890.56) | -55.91 |
| Eritrea | 169.25(91.97-237.24) | 121.5(54.5-181.21) | -28.21 | 3892.52(1916.08-5481.40) | 2612.03(1012.17-3994.94) | -32.90 |
| Estonia | 212.35(0.74-327.88) | 80.37(29.46-112.24) | -62.15 | 4221.15(97.02-6195.33) | 1362.95(455.03-1902.47) | -67.71 |
| Eswatini | 111.54(50.59-155.91) | 105(41.28-164.39) | -5.86 | 2424.40(1024.38-3376.17) | 2405.44(804.19-3779.61) | -0.78 |
| Ethiopia | 148.78(75.02-205.3) | 74.49(34.51-105.63) | -49.93 | 3525.13(1600.76-4825.70) | 1615.75(669.16-2300.28) | -54.16 |
| Fiji | 193.97(90.87-271.45) | 151.45(53.45-232.69) | -21.92 | 4896.82(2258.4-6749.76) | 3610.08(1209.87-5441.53) | -26.28 |
| Finland | 111.20(-8.83-176.94) | 41.29(2.05-65.73) | -62.87 | 2352.56(-188.43-3587.96) | 747.90(25.74-1143.59) | -68.21 |
| France | 45.07(6.01-69.92) | 16.93(4.45-25.25) | -62.44 | 857.92(92.23-1268.31) | 344.33(92.34-490.06) | -59.86 |
| Gabon | 96.17(33.36-145.91) | 82.49(25.64-134.36) | -14.22 | 1995.65(608.21-2949.24) | 1641.67(402.33-2655.49) | -17.74 |
| Gambia | 103.08(23.27-165.35) | 118.04(18.93-191.60) | 14.51 | 2417.49(522.67-3838.48) | 2675.12(475.19-4235.91) | 10.66 |
| Georgia | 221.29(29.65-328.60) | 111.07(31.43-164.59) | -49.81 | 4531.83(484.80-6570.63) | 2308.94(550.4-3324.68) | -49.05 |
| Germany | 95.63(0.07-153.91) | 34.51(6.40-52.90) | -63.91 | 1861.68(-5.47-2848.70) | 639.65(99.16-950.34) | -65.64 |
| Ghana | 108.35(16.14-176.52) | 69.33(12.85-119.45) | -36.01 | 2541.85(326.72-4054.57) | 1564.61(255.60-2664.50) | -38.45 |
| Greece | 67.54(2.37-111.11) | 35.68(4.57-55.75) | -47.17 | 1317.14(11.08-2056.59) | 743.98(66.73-1119.77) | -43.52 |
| Greenland | 101.22(17.74-154.19) | 38.30(11.38-58.64) | -62.16 | 2279.81(486.01-3311.84) | 868.71(303.45-1264.28) | -61.90 |
| Grenada | 105.00(31.28-158.87) | 67.24(29.90-99.65) | -35.96 | 2505.91(678.84-3710.98) | 1420.54(615.77-2101.15) | -43.31 |
| Guam | 120.64(44.29-175.51) | 59.35(7.43-92.97) | -50.80 | 2512.03(838.24-3611.65) | 1723.86(173.29-2635.69) | -31.38 |
| Guatemala | 83.02(10.88-129.92) | 47.61(7.66-77.47) | -42.65 | 1727.49(175.86-2639.49) | 933.76(163.11-1491.83) | -45.95 |
| Guinea | 70.98(13.53-117.91) | 78.74(14.54-130.55) | 10.93 | 1602.74(286.06-2624.18) | 1758.83(302.62-2918.23) | 9.74 |
| Guinea-Bissau | 146.38(30.21-227.83) | 138.16(27.04-210.59) | -5.62 | 3614.75(779.05-5491.42) | 3241.12(662.99-4844.92) | -10.34 |
| Guyana | 197.72(54.97-302.98) | 108.83(32.5-171.49) | -44.96 | 4567.51(997.86-6995.24) | 2453.74(589.17-3951.52) | -46.28 |
| Haiti | 184.47(32.92-286.51) | 144.83(38.82-238.09) | -21.49 | 4231.35(715.91-6530.69) | 3278.19(867.54-5337.36) | -22.53 |
| Honduras | 86.77(21.57-130.85) | 109.77(23.56-170.87) | 26.51 | 1907.46(456.75-2833.40) | 2160.93(450.05-3326.66) | 13.29 |
| Hungary | 203.15(87.00-278.56) | 106.23(46.29-151.23) | -47.71 | 4351.24(1845.6-5775.93) | 2068.05(923.04-2862.54) | -52.47 |
| Iceland | 85.36(9.93-131.71) | 27.07(3.34-43.53) | -68.29 | 1712.19(212.7-2543.74) | 509.11(50.08-783.37) | -70.27 |
| India | 103.32(13.76-157.06) | 96.52(12.63-148.13) | -6.58 | 2637.63(296.03-3893.26) | 2347.03(257.81-3494.14) | -11.02 |
| Indonesia | 120.61(42.79-181.98) | 109.07(19.8-176.15) | -9.57 | 3053.14(1012.98-4467.17) | 2488.63(400.02-4045.89) | -18.49 |
| Iran (Islamic Republic of) | 151.28(30.77-230.82) | 72.08(13.52-114.44) | -52.35 | 3227.33(640.49-4787.87) | 1450.94(228.91-2241.79) | -55.04 |
| Iraq | 152.05(8.68-237.2) | 142.61(14.05-230.93) | -6.21 | 3632.73(150.38-5611.30) | 2999.36(268.25-4843.11) | -17.44 |
| Ireland | 109.12(-9.60-178.26) | 21.14(-2.32-36.65) | -80.63 | 2218.09(-222.57-3464.97) | 401.00(-52.82-665.76) | -81.92 |
| Israel | 63.94(12.08-104) | 12.23(3.70-19.88) | -80.87 | 1247.53(259.14-1973.29) | 239.78(77.41-372.08) | -80.78 |
| Italy | 60.89(14.43-93.84) | 29.07(12.4-42.20) | -52.26 | 1179.11(279.20-1735.22) | 498.00(199.39-703.80) | -57.76 |
| Jamaica | 69.09(31.42-99.08) | 47.80(20.14-74.16) | -30.81 | 1358.56(576.64-1941.53) | 1005.71(423.43-1544.17) | -25.97 |
| Japan | 46.9(18.4-71.04) | 15.52(5.26-24.36) | -66.91 | 908.57(375.37-1329.07) | 360.78(122.31-544.88) | -60.29 |
| Jordan | 131.67(26.4-203.17) | 65.28(23.3-97.76) | -50.42 | 2931.41(470.15-4449.82) | 1361.82(431.41-2028.09) | -53.54 |
| Kazakhstan | 210.85(43.82-300.30) | 140.62(24.73-212.59) | -33.31 | 4503.79(914.11-6179.45) | 2540.72(379.14-3727.54) | -43.59 |
| Kenya | 55.32(29.24-78.62) | 60.81(27.04-91.31) | 9.92 | 1168.57(550.63-1654.02) | 1228.43(471.39-1827.19) | 5.12 |
| Kiribati | 137.76(25.23-217.29) | 130.24(17.47-212.86) | -5.46 | 3642.33(410.34-5674.17) | 3415.54(267.89-5464.97) | -6.23 |
| Kuwait | 104.77(26.53-157.07) | 49.82(11.28-79.91) | -52.45 | 2407.05(573.69-3522.65) | 1139.58(228.89-1796.86) | -52.66 |
| Kyrgyzstan | 190.39(42.23-270.98) | 170.24(24.36-253.35) | -10.58 | 4018.00(817.73-5537.68) | 3232.32(476.84-4762.82) | -19.55 |
| Lao People's Democratic Republic | 250.15(82.40-376.32) | 117.86(20.07-190.70) | -52.88 | 6222.82(2010.25-9297.34) | 2585.55(387.81-4205.78) | -58.45 |
| Latvia | 215.11(26.78-318.24) | 98.76(18.84-152.81) | -54.09 | 4441.95(634.98-6227.49) | 1972.57(435.47-2913.54) | -55.59 |
| Lebanon | 94.49(29.98-147.60) | 36.17(8.71-57.56) | -61.72 | 2153.70(737.33-3255.21) | 765.36(181.17-1186.02) | -64.46 |
| Lesotho | 89.78(44.64-128.88) | 130.43(56.91-189.43) | 45.28 | 1887.49(884.07-2707.40) | 2942.14(1257.75-4237.61) | 55.88 |
| Liberia | 91.50(16.74-144.80) | 100.34(25.52-163.99) | 9.66 | 2065.05(357.79-3219.62) | 2249.17(569.43-3643.79) | 8.92 |
| Libya | 79.00(19.74-122.41) | 95.80(28.43-151.71) | 21.27 | 1812.65(440.04-2749.29) | 2173.30(625.58-3406.64) | 19.90 |
| Lithuania | 203.05(18.69-309.00) | 100.24(7.98-158.74) | -50.63 | 4041.39(460.98-5856.67) | 1894.34(183.02-2867.27) | -53.13 |
| Luxembourg | 83.74(4.57-131.55) | 23.78(2.39-37.31) | -71.60 | 1638.69(90.56-2461.14) | 441.47(38.78-671.00) | -73.06 |
| Madagascar | 159.24(89.65-220.26) | 145.88(65.88-219.15) | -8.39 | 3646.80(1931.77-5009.60) | 3286.57(1262.66-4935.38) | -9.88 |
| Malawi | 94.02(40.29-140.42) | 88.30(28.85-134.53) | -6.08 | 2095.40(749.5-3112.57) | 1954.04(527.45-3002.76) | -6.75 |
| Malaysia | 117.36(26.1-180.36) | 72.54(8.74-118.88) | -38.19 | 2815.35(656.98-4224.58) | 1694.47(184.73-2719.51) | -39.81 |
| Maldives | 146.78(49.52-217.84) | 50.70(8.73-81.99) | -65.46 | 3613.98(1148.66-5266.02) | 1053.86(155.10-1672.42) | -70.84 |
| Mali | 80.3(23.77-120.56) | 61.96(16.25-94.85) | -22.84 | 1881.72(594.85-2781.45) | 1412.95(374.25-2098.45) | -24.91 |
| Malta | 103.81(27.55-155.56) | 33.35(8.19-51.72) | -67.87 | 2050.17(606.81-2944.07) | 660.07(167.86-985.04) | -67.80 |
| Marshall Islands | 210.23(61.16-302.37) | 189.25(43.78-295.85) | -9.98 | 5125.53(1218.55-7334.23) | 4770.32(824.67-7537.29) | -6.93 |
| Mauritania | 143.89(47.85-212.24) | 103.18(41.51-158.95) | -28.29 | 3301.71(1148.2-4785.63) | 2215.10(907.58-3389.36) | -32.91 |
| Mauritius | 187.38(108.74-250.58) | 69.69(39.87-95.05) | -62.81 | 4441.04(2582.17-5814.77) | 1616.28(909.15-2156.89) | -63.61 |
| Mexico | 59.01(11.60-90.00) | 48.16(3.27-78.15) | -18.39 | 1130.26(201.09-1679.44) | 981.17(49.72-1541.59) | -13.19 |
| Micronesia (Federated States of) | 220.17(61.65-328.52) | 186.78(42.47-297.64) | -15.17 | 5504.20(1266.63-8181.54) | 4698.56(830.95-7596.34) | -14.64 |
| Monaco | 45.15(12.26-74.72) | 20.43(6.67-33.55) | -54.75 | 906.84(263.95-1445.16) | 408.02(143.2-625.27) | -55.01 |
| Mongolia | 262.57(80.78-356.23) | 152.84(36.75-215.47) | -41.79 | 5428.99(1754.56-7233.69) | 2987.32(802.59-4085.65) | -44.97 |
| Montenegro | 126.08(57.16-177.46) | 142.34(56.44-208.56) | 12.90 | 2590.69(1226.95-3561.58) | 2411.99(934.02-3458.31) | -6.90 |
| Morocco | 179.43(31.86-273.33) | 130.49(19.63-215.74) | -27.28 | 4129.05(708.13-6129.70) | 2759.17(383.65-4465.59) | -33.18 |
| Mozambique | 124.73(67.09-175.77) | 121.54(58.98-184.25) | -2.56 | 2697.59(1288.95-3806.52) | 2649.73(1162.41-3988.69) | -1.77 |
| Myanmar | 206.35(75.70-312.31) | 100.23(27.10-161.69) | -51.43 | 5109.99(1662.91-7702.60) | 2227.56(571.82-3523.95) | -56.41 |
| Namibia | 130.17(59.02-181.84) | 106.59(43.83-156.25) | -18.11 | 2862.48(1232.64-3982.24) | 2219.79(862.81-3251.03) | -22.45 |
| Nauru | 242.68(54.72-375.28) | 239.81(29.75-389.13) | -1.18 | 6263.13(776.75-9647.17) | 6306.64(340.04-10244.29) | 0.69 |
| Nepal | 107.83(21.32-162.08) | 92.61(23.37-141.42) | -14.11 | 2599.68(391.74-3881.09) | 2010.55(441.93-3045.49) | -22.66 |
| Netherlands | 62.59(6.68-99.62) | 17.98(3.5-28.47) | -71.27 | 1314.31(154.7-2013.32) | 331.33(62.67-505.85) | -74.79 |
| New Zealand | 81.34(-7.64-134.73) | 26.00(-0.93-43.35) | -68.04 | 1701.07(-169.01-2684.71) | 499.77(-15.84-786.22) | -70.62 |
| Nicaragua | 67.05(22.06-96.90) | 51.85(14.99-79.45) | -22.67 | 1436.68(464.93-2043.12) | 1071.26(306.77-1617.74) | -25.44 |
| Niger | 73.95(20.57-112.02) | 62.26(20.88-99.59) | -15.81 | 1682.05(506.24-2490.00) | 1360.60(466.54-2149.92) | -19.11 |
| Nigeria | 84.91(20.72-137.54) | 60.07(6.43-101.32) | -29.25 | 1816.62(397.05-2918.36) | 1252.29(118.88-2068.82) | -31.06 |
| Niue | 155.75(37.14-243.89) | 130.71(22.9-210.49) | -16.08 | 3661.64(727.78-5750.80) | 3053.70(400.25-4867.02) | -16.60 |
| North Macedonia | 194.55(86.17-276.81) | 165.61(64.69-247.04) | -14.88 | 3946.12(1717.45-5525.56) | 2704.55(1027.8-4046.77) | -31.46 |
| Northern Mariana Islands | 74.44(21.31-121.85) | 73.78(13.05-117.01) | -0.89 | 1734.32(457.16-2777.57) | 1735.30(195.03-2693.84) | 0.06 |
| Norway | 91.79(25.19-137.16) | 20.13(6.50-29.94) | -78.07 | 1930.37(576.68-2760.23) | 388.16(134.14-557.17) | -79.89 |
| Oman | 172.62(6.17-272.21) | 87.69(-0.83-146.54) | -49.20 | 3870.04(-13.98-6109.47) | 1750.34(-106.32-2912.63) | -54.77 |
| Pakistan | 112.15(27.15-170.31) | 132.16(26.85-202.18) | 17.84 | 2657.63(630.03-3915.46) | 3075.59(597.17-4721.77) | 15.73 |
| Palau | 141.65(23.09-227.16) | 116.58(7.84-188.22) | -17.70 | 3461.69(518.6-5449.19) | 2874.13(120.44-4562.57) | -16.97 |
| Palestine | 210.39(33.63-327.03) | 116.34(15.35-178.26) | -44.70 | 4236.76(548.16-6530.39) | 2211.04(172.42-3389.49) | -47.81 |
| Panama | 57.29(19.74-85.54) | 30.42(12.43-47.22) | -46.90 | 1179.16(452.81-1701.3) | 653.02(272.77-987.34) | -44.62 |
| Papua New Guinea | 121.58(36.58-192.62) | 112.01(27.4-177.45) | -7.87 | 2955.08(830.42-4625.57) | 2716.22(581.66-4288.94) | -8.08 |
| Paraguay | 69.93(23.43-103.82) | 50.78(24.15-75.92) | -27.38 | 1472.50(531.37-2117.46) | 1074.78(537.16-1563.78) | -27.01 |
| Peru | 52.61(11.99-80.53) | 24.27(4.07-40.65) | -53.87 | 1148.80(238.67-1736.08) | 537.47(77.32-870.92) | -53.21 |
| Philippines | 125.74(35.46-187.45) | 98.73(26.19-155.61) | -21.48 | 2764.13(573.01-4048.87) | 2313.01(465.17-3604.17) | -16.32 |
| Poland | 190.06(41.77-271.60) | 73.05(17.72-107.43) | -61.56 | 4004.32(762.61-5512.40) | 1357.31(289.51-1974.15) | -66.10 |
| Portugal | 59.03(5.96-97.15) | 19.50(4.25-30.30) | -66.97 | 1127.17(134.28-1768.99) | 395.51(70.03-594.76) | -64.91 |
| Puerto Rico | 77.39(20.45-119.51) | 26.74(10.88-40.04) | -65.45 | 1584.73(436.63-2393.59) | 606.07(231.96-893.17) | -61.76 |
| Qatar | 137.84(2.16-236.18) | 36.31(2.87-64.08) | -73.66 | 2712.27(25.74-4580.72) | 699.07(56.67-1219.04) | -74.23 |
| Republic of Korea | 76.60(31.16-120.75) | 21.74(10.86-32.96) | -71.62 | 1455.97(612.23-2285.95) | 377.13(190.64-558.02) | -74.10 |
| Republic of Moldova | 197.39(-19.93-324.45) | 127.55(2.46-201.53) | -35.38 | 3639.03(-329.88-5696.60) | 2615.41(13.03-4029.6) | -28.13 |
| Romania | 224.92(99.77-313.08) | 118.43(47.61-171.13) | -47.35 | 4210.96(1883.06-5721.00) | 2250.23(867.89-3196.67) | -46.56 |
| Russian Federation | 199.62(22.68-298.52) | 115.70(5.54-181.41) | -42.04 | 4252.47(671.31-6035.54) | 2468.96(176.47-3727.01) | -41.94 |
| Rwanda | 160.04(78.63-241.90) | 69.66(28.21-111.33) | -56.47 | 3653.36(1601.51-5496.46) | 1393.42(535.45-2207.5) | -61.86 |
| Saint Kitts and Nevis | 148.08(44.09-221.73) | 70.08(30.44-103.74) | -52.67 | 3177.98(857.59-4672.56) | 1381.16(583.44-2030.69) | -56.54 |
| Saint Lucia | 114.9(36.49-172.34) | 48.42(22.63-73.36) | -57.86 | 2162.82(689.14-3141.89) | 950.41(432.32-1400.42) | -56.06 |
| Saint Vincent and the Grenadines | 137.10(39.58-200.26) | 85.74(38.43-122.66) | -37.46 | 2801.19(737.59-4048.81) | 1639.24(680.45-2330.53) | -41.48 |
| Samoa | 146.19(33.40-221.93) | 137.95(24.41-219.84) | -5.64 | 3469.59(698.91-5216.98) | 3320.87(394.85-5239.17) | -4.29 |
| San Marino | 40.17(7.10-64.04) | 13.11(3.25-22.89) | -67.36 | 735.28(122.42-1139.81) | 260.69(61.98-440.33) | -64.55 |
| Sao Tome and Principe | 63.36(12.02-101.24) | 62.05(5.78-106.14) | -2.07 | 1470.93(256.11-2296.15) | 1396.15(111.72-2372.21) | -5.08 |
| Saudi Arabia | 143.22(17.99-226.43) | 111.41(14.01-172.88) | -22.21 | 3204.35(200.62-5015.64) | 2655.89(174.89-4100.60) | -17.12 |
| Senegal | 96.30(16.07-155.76) | 81.59(17.96-137.85) | -15.28 | 2243.23(395.6-3577.82) | 1789.27(401.63-2932.57) | -20.24 |
| Serbia | 247.59(105.83-348.64) | 127.8(49.08-190.78) | -48.38 | 4378.19(1878.41-6016.78) | 2234.47(830.8-3318.83) | -48.96 |
| Seychelles | 149.52(72.17-205.73) | 83.27(34.37-121.89) | -44.31 | 3401.36(1408.11-4676.12) | 1760.51(611.18-2563.26) | -48.24 |
| Sierra Leone | 85.73(6.15-146.92) | 82.16(7.71-139.43) | -4.16 | 1982.45(132.93-3330.62) | 1880.73(151.23-3158.47) | -5.13 |
| Singapore | 84.15(23.29-127.57) | 22.41(5.97-34.46) | -73.37 | 1886.8(461.81-2833.29) | 494.51(132.07-743.07) | -73.79 |
| Slovakia | 219.37(67.37-309.53) | 111.9(37.8-166.67) | -48.99 | 4557.73(1242-6260.77) | 2029.09(683.29-2976.48) | -55.48 |
| Slovenia | 105.18(43.53-146.61) | 39.96(18.71-56.22) | -62.01 | 2110.73(858.89-2865.63) | 698.65(302.36-985.39) | -66.90 |
| Solomon Islands | 230.89(55.73-359.66) | 198.63(37.48-318.12) | -13.97 | 5582.88(1167.98-8807.46) | 4868.11(778.61-7788.82) | -12.80 |
| Somalia | 149.26(80.55-211.63) | 114.94(49.6-176.02) | -22.99 | 3419.37(1694.6-4872.52) | 2601.44(1067.94-4050.75) | -23.92 |
| South Africa | 65.04(23.92-91.83) | 69.13(30.99-94.84) | 6.29 | 1572.60(529.48-2140.7) | 1479.58(642.54-1992.32) | -5.92 |
| South Sudan | 106.76(54.29-159.85) | 84.67(36.57-135.33) | -20.69 | 2346.54(1083.83-3520.56) | 1882.41(683.94-3017.80) | -19.78 |
| Spain | 40.66(-1.87-67.29) | 15.60(2.46-24.86) | -61.63 | 816.17(-20.48-1256.4) | 319.23(37.21-481.94) | -60.89 |
| Sri Lanka | 126.93(44.27-187.49) | 65.56(12.79-114.22) | -48.35 | 2745.54(914.5-3983.02) | 1422.21(248.04-2445.37) | -48.20 |
| Sudan | 229.21(33.16-349.68) | 156.42(16.62-258.06) | -31.76 | 5437.57(627.88-8351.29) | 3510.74(294.74-5715.09) | -35.44 |
| Suriname | 101.46(28.48-155.11) | 57.03(26.25-88.18) | -43.79 | 2322.43(554.33-3499.41) | 1342.37(588.99-2044.61) | -42.20 |
| Sweden | 88.34(1.42-142.42) | 26.77(3.69-42.66) | -69.70 | 1693.37(43.26-2606.84) | 480.15(60.42-743.95) | -71.65 |
| Switzerland | 70.78(-1.16-114.27) | 23.33(4.85-36.01) | -67.04 | 1330.30(-63.16-2044.28) | 388.54(59.50-585.38) | -70.79 |
| Syrian Arab Republic | 187.14(0.42-306.72) | 173.91(10.49-290.26) | -7.07 | 4259.12(-158.67-6846.93) | 3566.43(105.29-5917.62) | -16.26 |
| Taiwan (Province of China) | 57.01(33.75-84.87) | 20.08(12.64-29.44) | -64.78 | 1076.74(660.99-1600.71) | 412.04(263.76-596.82) | -61.73 |
| Tajikistan | 231.74(62.18-334.00) | 170.95(33.45-256.54) | -26.23 | 4785.95(1128.81-6774.58) | 3253.04(556.44-4811.59) | -32.03 |
| Thailand | 61.38(14.82-97.91) | 26.93(4.17-47.49) | -56.13 | 1408.42(345.39-2182.55) | 664.86(108.04-1137.14) | -52.79 |
| Timor-Leste | 146.30(58.86-212.48) | 140.80(39.58-217.36) | -3.76 | 3377.13(1327.6-4898.24) | 3119.92(861.8-4788.13) | -7.62 |
| Togo | 105.45(23.69-168.11) | 106.59(27.52-174.52) | 1.08 | 2481.60(570.06-3896.26) | 2463.75(644.47-3925.75) | -0.72 |
| Tokelau | 173.98(50.28-262.35) | 121.39(23.84-197.61) | -30.23 | 4018.62(1028.28-6084.24) | 2864.59(440.17-4570.56) | -28.72 |
| Tonga | 88.01(15.1-136.09) | 77.85(9.81-128.59) | -11.54 | 2110.42(287.42-3211.96) | 1854.30(159.92-2990.58) | -12.14 |
| Trinidad and Tobago | 140.03(64.69-198.36) | 59.33(28.29-94.60) | -57.63 | 2999.73(1343.05-4210.92) | 1389.57(674.77-2183.05) | -53.68 |
| Tunisia | 122.27(26.34-187.13) | 79.87(13.41-138.81) | -34.68 | 2483.71(512.72-3764.48) | 1582.23(194.49-2746.90) | -36.30 |
| Turkey | 95.31(-5.38-159.35) | 49.38(-4.74-87.77) | -48.19 | 2144.02(-207.01-3498.83) | 979.46(-129.55-1729.66) | -54.32 |
| Turkmenistan | 260.91(37.76-372.82) | 206.33(18.13-326.28) | -20.92 | 5431.94(692.52-7486.53) | 4225.71(340.21-6700.78) | -22.21 |
| Tuvalu | 229.24(65.75-338.02) | 174.85(32.85-266.2) | -23.73 | 5610.17(1437.28-8284.83) | 4319.61(633.69-6570.47) | -23.00 |
| Uganda | 79.14(29.62-127.63) | 56.81(18.51-93.97) | -28.22 | 1738.51(562.98-2752.68) | 1215.01(322.01-1967.09) | -30.11 |
| Ukraine | 181.27(0.02-291.32) | 181.47(-7.46-314.65) | 0.11 | 3490.39(7.78-5380.27) | 3473.60(-123.4-5928.07) | -0.48 |
| United Arab Emirates | 98.66(9.19-161.73) | 70.02(11.07-114.74) | -29.03 | 2229.25(170.14-3578.96) | 1294.21(182.59-2079.55) | -41.94 |
| United Kingdom | 104.83(12.05-161.13) | 27.97(5.33-42.07) | -73.32 | 2198.59(252.8-3247.82) | 580.41(112.24-833.50) | -73.60 |
| United Republic of Tanzania | 97.17(49.63-139.29) | 80.80(28.03-127.04) | -16.85 | 2079.25(909.76-3015.93) | 1659.55(477.35-2663.16) | -20.19 |
| United States of America | 82.41(22.24-124.69) | 40.28(18.36-56.40) | -51.12 | 1731.53(499.94-2491.64) | 921.56(454.03-1230.33) | -46.78 |
| United States Virgin Islands | 100.57(28.26-157.90) | 42.14(17.08-66.23) | -58.10 | 2052.67(557.26-3133.59) | 876.45(371.48-1351.12) | -57.30 |
| Uruguay | 105.87(22.67-151.02) | 45.00(17.63-63.18) | -57.50 | 2198.13(503.78-2999.93) | 918.48(366.54-1242.81) | -58.22 |
| Uzbekistan | 211.71(34.77-300.81) | 202.12(12.50-305.99) | -4.53 | 4419.49(717.23-6077.39) | 3959.43(223.99-5943.50) | -10.41 |
| Vanuatu | 195.11(41.33-299.41) | 179.24(23.88-280.57) | -8.13 | 4775.51(715.50-7314.55) | 4491.12(471.10-6974.07) | -5.96 |
| Venezuela (Bolivarian Republic of) | 104.46(38.39-149.18) | 80.59(29.26-127.03) | -22.85 | 2329.53(870.01-3262.45) | 1773.26(643.72-2737.38) | -23.88 |
| Viet Nam | 104.60(45.02-159.30) | 67.61(25.78-109.77) | -35.36 | 2313.06(977.71-3503.43) | 1383.23(562.30-2216.00) | -40.20 |
| Yemen | 256.29(48.09-394.74) | 204.16(39.24-320.67) | -20.34 | 5955.15(951.16-9123.27) | 4450.62(683.78-7059.93) | -25.26 |
| Zambia | 138.95(75.16-195.24) | 127.55(59.11-191.53) | -8.20 | 2995.19(1476.64-4240.18) | 2673.92(1104.09-4101.01) | -10.73 |
| Zimbabwe | 85.86(31.11-129.10) | 117.23(42.29-179.79) | 36.54 | 1783.77(640.18-2638.76) | 2679.42(987.24-4095.81) | 50.21 |

Note: ASR: age-standardized rates; DALY, disability-adjusted life years; UI, uncertainty interval

# Table S3 Mortality and disability-adjusted life years attributable to dietary factors of Diabetes mellitus patients in 204 countries and regions from 1990 to 2021

| **Countries or territories** | **ASR of Deaths (per 100,000 population)** | | | **ASR of DALYs (per 100,000 population)** | | |
| --- | --- | --- | --- | --- | --- | --- |
|  | **1990(95%UI)** | **2021(95%UI)** | **Percentage changes(%)** | **1990(95%UI)** | **2021(95%UI)** | **Percentage changes(%)** |
| Global | 4.55(0.86-7.35) | 4.52(0.88-7.36) | -0.66 | 159.96(31.17-262.82) | 221.34(47.97-368.92) | 38.37 |
| Afghanistan | 9.01(1.08-15.90) | 14.13(2.02-24.72) | 56.83 | 366.82(43.89-635.44) | 726.60(101.83-1272.78) | 98.08 |
| Albania | 2.05(0.6-3.12) | 2.09(0.63-3.26) | 1.95 | 129.04(38.95-207.53) | 182.17(52.98-291.81) | 41.17 |
| Algeria | 3.09(0.82-5.14) | 4.55(1.23-7.41) | 47.25 | 139.28(37.78-230.11) | 284.39(85.10-484.60) | 104.19 |
| American Samoa | 20.35(3.36-34.50) | 25.18(4.86-43.39) | 23.73 | 655.48(116.36-1108.48) | 1008.06(212.94-1764.03) | 53.79 |
| Andorra | 4.86(1.21-7.88) | 3.31(0.78-5.58) | -31.89 | 150.33(39.39-240.40) | 188.06(47.86-306.95) | 25.10 |
| Angola | 14.67(1.3-25.99) | 8.93(1.52-15.57) | -39.13 | 430.07(39.09-752.37) | 304.62(59.65-530.87) | -29.17 |
| Antigua and Barbuda | 15.73(3.64-24.97) | 13.56(3.14-21.84) | -13.80 | 480.37(115.62-772.94) | 515.33(128.71-862.30) | 7.28 |
| Argentina | 9.55(2.05-14.66) | 6.57(1.58-10.11) | -31.20 | 293.44(69.94-456.96) | 316.94(82.85-515.79) | 8.01 |
| Armenia | 5.65(1.37-8.90) | 4.75(1.02-7.38) | -15.93 | 217.75(55.44-352.90) | 247.28(61.02-404.98) | 13.56 |
| Australia | 4.17(0.76-6.70) | 3.22(0.63-5.07) | -22.78 | 135.35(26.20-221.06) | 166.55(35.76-280.30) | 23.05 |
| Austria | 4.65(0.74-7.52) | 3.16(0.62-5.12) | -32.04 | 118.72(20.09-192.68) | 131.64(28.21-220.34) | 10.88 |
| Azerbaijan | 3.83(1.04-6.00) | 6.4(1.65-10.66) | 67.10 | 157.37(44.88-241.72) | 308.24(84.83-489.64) | 95.87 |
| Bahamas | 11.45(1.76-19) | 9.08(1.74-15.35) | -20.70 | 397.37(65.02-676.48) | 449.99(92.74-783.09) | 13.24 |
| Bahrain | 26.64(6.68-42.73) | 31.28(7.62-51.43) | 17.42 | 668.20(176.01-1061.64) | 876.10(237.98-1429.19) | 31.11 |
| Bangladesh | 8.65(0.53-15.08) | 8.93(1.33-15.51) | 3.24 | 239.45(15.77-416.97) | 278.65(43.52-484.63) | 16.37 |
| Barbados | 19.8(3.59-32.48) | 13.98(2.84-24.14) | -29.39 | 578.67(110.85-959.38) | 508.45(116.63-874.26) | -12.13 |
| Belarus | 1.73(0.35-2.58) | 1.70(0.33-2.66) | -1.73 | 101.35(23.21-162.46) | 132.91(29.30-216.32) | 31.14 |
| Belgium | 4.21(0.93-6.57) | 2.03(0.48-3.09) | -51.78 | 144.07(35.64-232.21) | 194.78(49.24-318.16) | 35.20 |
| Belize | 7.88(1.64-13.48) | 8.20(1.90-13.85) | 4.06 | 249.95(51.57-433.90) | 323.92(82.75-565.12) | 29.59 |
| Benin | 6.19(1.38-10.29) | 7.54(1.58-13.07) | 21.81 | 194.94(45.18-320.25) | 285.22(67.41-477.78) | 46.31 |
| Bermuda | 7.71(1.04-13.27) | 4.11(0.50-7.25) | -46.69 | 232.63(32.49-399.79) | 237.64(32.78-425.32) | 2.15 |
| Bhutan | 5.63(0.83-9.78) | 6.65(1.31-11.70) | 18.12 | 176.12(29.99-298.41) | 225.00(50.12-393.63) | 27.75 |
| Bolivia (Plurinational State of) | 8.53(1.26-15.06) | 8.58(1.21-15.67) | 0.59 | 255.59(39.12-438.65) | 303.56(44.96-562.02) | 18.77 |
| Bosnia and Herzegovina | 4.15(1.08-6.54) | 8.03(2.19-13.53) | 93.49 | 175.71(46.12-281.13) | 332.94(95.57-560.24) | 89.48 |
| Botswana | 14.33(1.11-26.22) | 13.42(1.6-24.73) | -6.35 | 359.99(30.24-651.42) | 379.68(49.62-667.09) | 5.47 |
| Brazil | 6.76(0.87-11.68) | 6.36(0.91-10.86) | -5.92 | 237.52(33.57-408.49) | 272.01(42.96-471.24) | 14.52 |
| Brunei Darussalam | 18.51(3.14-32.09) | 11.97(2.55-19.96) | -35.33 | 518.25(91.86-897.91) | 531.45(127.25-904.34) | 2.55 |
| Bulgaria | 6.72(1.58-10.16) | 6.29(1.43-9.85) | -6.40 | 263.22(67.96-418.49) | 339.8(84.28-555.66) | 29.09 |
| Burkina Faso | 11.05(1.49-18.54) | 10.85(1.49-18.38) | -1.81 | 292.18(42.20-491.51) | 362.97(50.60-607.18) | 24.23 |
| Burundi | 11.61(1.15-21.22) | 10.25(1.54-18.97) | -11.71 | 300.55(34.17-537.21) | 277.27(48.34-491.64) | -7.75 |
| Cabo Verde | 2.33(0.41-3.95) | 6.95(1.22-12.01) | 198.28 | 106.40(20.11-183.01) | 267.60(51.56-458.74) | 151.50 |
| Cambodia | 5.10(0.27-9.66) | 5.86(0.41-11.46) | 14.9 | 151.53(8.66-283.79) | 214.66(16.13-400.34) | 41.66 |
| Cameroon | 6.56(1.33-11.24) | 8.54(1.85-15.15) | 30.18 | 183.52(37.25-311.91) | 269.71(57.93-469.99) | 46.96 |
| Canada | 4.09(0.75-6.62) | 2.6(0.56-4.08) | -36.43 | 121.36(22.61-200.12) | 204.64(47.27-352.15) | 68.62 |
| Central African Republic | 19.13(0.24-32.88) | 20.26(0.4-37.17) | 5.91 | 562.62(10.5-983.35) | 688.51(18.87-1212.52) | 22.38 |
| Chad | 9.71(1.57-16.89) | 15.31(2.71-25.58) | 57.67 | 295.85(47.4-491.24) | 512.95(87.89-838.93) | 73.38 |
| Chile | 6.69(1.76-10.06) | 4.65(1.16-6.96) | -30.49 | 237.45(67.94-367.61) | 304.01(83.20-504.28) | 28.03 |
| China | 1.75(0.21-3.10) | 1.85(0.21-3.34) | 5.71 | 85.11(10.99-153.97) | 133.19(16.98-244.54) | 56.49 |
| Colombia | 3.35(0.48-5.92) | 2.52(0.51-4.37) | -24.78 | 146.74(23.74-260.94) | 183.09(42.86-330.23) | 24.77 |
| Comoros | 10.98(0.09-19.69) | 11.38(0.42-21) | 3.64 | 301.05(5.72-538.73) | 353.66(18.72-640.85) | 17.48 |
| Congo | 17.21(0.54-30.11) | 16.41(0.87-29.66) | -4.65 | 467.70(19.29-812.3) | 502.79(33.93-904.17) | 7.50 |
| Cook Islands | 33.17(6.06-56.9) | 25.18(5.23-43.89) | -24.09 | 952.76(184.9-1615.75) | 959.17(215.18-1657.26) | 0.67 |
| Costa Rica | 3.62(0.63-6.10) | 4.19(1.03-7.01) | 15.75 | 185.79(35.11-326.36) | 262.43(72.76-448.83) | 41.25 |
| Cote d'Ivoire | 7.35(1.49-12.22) | 9.60(2.03-16.73) | 30.61 | 216.98(44.72-356.71) | 324.14(73.26-539.58) | 49.39 |
| Croatia | 2.93(0.73-4.75) | 3.69(0.93-5.89) | 25.94 | 129.63(34.76-218.97) | 185.64(50.68-319.44) | 43.21 |
| Cuba | 4.46(1.02-7.22) | 1.96(0.55-3.23) | -56.05 | 179.92(43.45-304.88) | 177.25(51.21-315.37) | -1.48 |
| Cyprus | 28.97(7.59-45.47) | 10.35(2.44-16.71) | -64.27 | 558.36(144.98-878.49) | 309.34(79.68-492.12) | -44.6 |
| Czechia | 4.25(0.82-6.89) | 5.83(1.23-9.78) | 37.18 | 181.23(37.39-300.32) | 270.39(60.29-462.81) | 49.20 |
| Democratic People's Republic of Korea | 1.40(0.22-2.54) | 1.61(0.26-2.94) | 15.00 | 55.93(9.37-101.70) | 89.72(16.71-157.68) | 60.41 |
| Democratic Republic of the Congo | 12.23(0.16-22.89) | 13.58(0.18-25.11) | 11.04 | 329.28(9.88-600.36) | 417.07(11.98-754.02) | 26.66 |
| Denmark | 3.46(0.60-5.55) | 3.95(0.83-6.11) | 14.16 | 108.32(20.24-174.11) | 162.04(38.86-267.74) | 49.59 |
| Djibouti | 10.31(2.01-17.76) | 13.01(2.68-22.42) | 26.19 | 262.14(52.49-443.89) | 355.62(74.97-601.27) | 35.66 |
| Dominica | 19.73(5.09-31.85) | 21.14(5.61-34.52) | 7.15 | 583.39(163.11-947.71) | 752.52(216.90-1207.46) | 28.99 |
| Dominican Republic | 3.26(0.72-5.60) | 4.06(0.80-7.26) | 24.54 | 131.62(34.10-222.49) | 236.41(56.94-419.81) | 79.62 |
| Ecuador | 5.44(1.09-9.22) | 6.85(1.13-11.74) | 25.92 | 199.05(45.16-337.86) | 321.53(64.32-549.48) | 61.53 |
| Egypt | 4.32(1.02-7.23) | 8.54(1.88-15.04) | 97.69 | 121.28(28.86-202.75) | 297.17(74.79-508.48) | 145.03 |
| El Salvador | 3.80(0.67-6.35) | 6.88(1.84-11.81) | 81.05 | 146.34(28.73-250.73) | 281.32(77.91-474.77) | 92.24 |
| Equatorial Guinea | 15.32(0.64-27.96) | 13.42(2.04-25.35) | -12.40 | 433.49(20.36-781.48) | 428.09(71.71-797.03) | -1.25 |
| Eritrea | 12.62(0.71-23.49) | 12.87(1.26-23.77) | 1.98 | 347.49(24.73-631.63) | 370.29(42.18-663.91) | 6.56 |
| Estonia | 1.68(0.36-2.53) | 3.81(0.85-6.09) | 126.79 | 111.79(28.34-180.64) | 220.02(52.86-355.86) | 96.82 |
| Eswatini | 19.16(1.06-33.75) | 27.15(1.30-50.99) | 41.70 | 483.28(29.02-858.48) | 749.12(37.38-1416.84) | 55.01 |
| Ethiopia | 22.89(2.91-37.82) | 11.24(1.7-18.78) | -50.90 | 615.88(80.91-1010.38) | 321.81(54.31-538.67) | -47.75 |
| Fiji | 56.55(6.73-96.87) | 69.84(10.94-121.19) | 23.5 | 1564.95(192.91-2655.79) | 1978.51(322.59-3413.58) | 26.43 |
| Finland | 2.33(0.42-3.71) | 1.42(0.31-2.23) | -39.06 | 121.67(24.62-200.70) | 187.21(45.49-314.46) | 53.87 |
| France | 3.85(0.74-5.97) | 3.07(0.66-4.70) | -20.26 | 112.05(23.95-175.82) | 147.81(37.40-238.48) | 31.91 |
| Gabon | 13.96(-0.13-27.18) | 15.25(0.86-29.15) | 9.24 | 384.98(-1.44-724.07) | 475.32(33.10-881.53) | 23.47 |
| Gambia | 8.15(1.00-14.30) | 12.6(1.83-22.11) | 54.60 | 237.70(30.35-403.42) | 397.75(56.90-701.59) | 67.33 |
| Georgia | 3.73(0.94-5.73) | 6.36(1.69-9.86) | 70.51 | 162.18(44.16-259.18) | 329.90(92.96-530.54) | 103.42 |
| Germany | 5.36(1.04-8.58) | 3.46(0.74-5.42) | -35.45 | 137.24(28.86-220.73) | 178.19(43.88-289.33) | 29.84 |
| Ghana | 6.01(1.38-10.1) | 9.76(2.47-16.38) | 62.40 | 181.95(45.19-296.15) | 316.03(81.67-520.83) | 73.69 |
| Greece | 3.22(0.76-4.97) | 2.56(0.59-3.89) | -20.50 | 151.45(39.80-245.85) | 218.95(55.54-356.66) | 44.57 |
| Greenland | 5.65(1.32-8.81) | 3.56(0.90-5.57) | -36.99 | 147.92(35.79-227.53) | 187.23(46.86-298.50) | 26.58 |
| Grenada | 21.76(4.17-35.97) | 19.03(4.12-31.53) | -12.55 | 633.98(125.07-1034.04) | 653.54(165.29-1096.64) | 3.09 |
| Guam | 9.69(2.02-16.06) | 4.70(1.19-7.81) | -51.50 | 305.68(65.58-513.96) | 313.28(82.51-536.44) | 2.49 |
| Guatemala | 2.76(0.62-4.59) | 9.92(2.65-16.85) | 259.42 | 111.54(28.95-189.61) | 398.98(106.67-674.62) | 257.70 |
| Guinea | 4.35(1.01-7.45) | 6.92(1.5-12.44) | 59.08 | 126.62(29.68-218.17) | 220.94(49.44-390.78) | 74.49 |
| Guinea-Bissau | 11.89(0.34-21.11) | 15.06(0.8-27.08) | 26.66 | 354.25(12.58-612.42) | 481.74(30.08-853.59) | 35.99 |
| Guyana | 15.05(2.34-26.8) | 14.07(3.74-24.40) | -6.51 | 527.29(84.18-938.37) | 610.27(178.42-1029.46) | 15.74 |
| Haiti | 17.93(2.23-31.8) | 18.18(1.15-35.67) | 1.39 | 541.86(73.21-936.85) | 656.55(46.17-1222.67) | 21.17 |
| Honduras | 2.35(0.36-4.17) | 4.51(1.07-8.01) | 91.91 | 122.67(19.93-215.88) | 236.35(60.62-412.29) | 92.67 |
| Hungary | 4.17(0.90-6.65) | 4.83(1.24-7.41) | 15.83 | 197.76(45.48-327.98) | 279.42(73.17-457.81) | 41.29 |
| Iceland | 2.44(0.52-3.72) | 1.60(0.30-2.52) | -34.43 | 107.85(26.52-169.18) | 156.15(36.97-264.39) | 44.78 |
| India | 4.03(0.83-6.98) | 5.53(1.26-9.14) | 37.22 | 129.67(27.66-223.58) | 194.55(47.19-325.89) | 50.03 |
| Indonesia | 2.93(0.24-5.42) | 2.54(0.47-4.57) | -13.31 | 99.95(8.66-184.02) | 93.01(17.09-162.24) | -6.94 |
| Iran (Islamic Republic of) | 3.44(0.92-5.68) | 5.35(1.42-8.45) | 55.52 | 128.89(33.22-210.74) | 258.12(75.46-419.29) | 100.26 |
| Iraq | 8.81(2.42-14.24) | 10.5(2.63-17.57) | 19.18 | 324.21(89.38-526.88) | 481(129.42-811.46) | 48.36 |
| Ireland | 4.02(0.72-6.37) | 1.78(0.35-2.80) | -55.72 | 133.09(26.75-216.35) | 131.26(30.69-221.59) | -1.38 |
| Israel | 7.37(2.13-11.24) | 6.83(1.87-10.56) | -7.33 | 232.49(71.30-361.78) | 272.84(83.75-439.24) | 17.36 |
| Italy | 8.3(1.91-12.57) | 4.41(1.02-6.71) | -46.87 | 236.56(56.68-360.77) | 204.05(53.94-324.28) | -13.74 |
| Jamaica | 12.94(3.5-21.17) | 13.52(3.53-22.82) | 4.48 | 378.08(106.18-613.33) | 444.96(122.44-740.99) | 17.69 |
| Japan | 1.9(0.44-3.04) | 0.62(0.15-0.99) | -67.37 | 117.50(30.01-195.74) | 163.21(42.27-277.70) | 38.90 |
| Jordan | 14.16(3.95-22.37) | 10.21(2.6-16.70) | -27.90 | 435.52(122.66-699.58) | 482.92(138.56-795.88) | 10.88 |
| Kazakhstan | 3.00(0.65-4.53) | 3.74(0.93-5.91) | 24.67 | 184.15(44.29-300.21) | 321.08(83.25-520.92) | 74.36 |
| Kenya | 5.05(0.79-9.15) | 6.8(1.40-12.06) | 34.65 | 130.05(20.11-230.23) | 183.97(40.11-312.40) | 41.46 |
| Kiribati | 30.76(6.11-53.1) | 38.63(8.29-67.75) | 25.59 | 891.29(184.23-1507.87) | 1179.74(256.19-2033.74) | 32.36 |
| Kuwait | 4.84(0.82-8.26) | 6.04(1.33-10.50) | 24.79 | 235.75(42.96-414.16) | 422.94(101.68-740.95) | 79.40 |
| Kyrgyzstan | 2.21(0.47-3.35) | 3.14(0.69-4.81) | 42.08 | 125.52(29.11-198.53) | 222.25(56.54-359.40) | 77.06 |
| Lao People's Democratic Republic | 7.58(-0.20-14.33) | 3.47(0.42-6.48) | -54.22 | 234.54(-4.90-447.33) | 135.88(17.92-257.58) | -42.07 |
| Latvia | 2.47(0.56-3.64) | 5.39(1.15-8.40) | 118.22 | 133.30(33.04-205.83) | 271.10(67.02-434.51) | 103.38 |
| Lebanon | 9.84(2.39-15.76) | 7.94(1.96-13.28) | -19.31 | 329.98(88.52-532.49) | 426.61(112.01-719.26) | 29.28 |
| Lesotho | 10.62(1.09-19.35) | 24.72(2.74-44.34) | 132.77 | 265.29(29.67-472.39) | 672.26(78.44-1219.60) | 153.41 |
| Liberia | 7.07(0.85-12.50) | 11.04(1.56-20.00) | 56.15 | 203.75(29.18-357.32) | 368.91(56.39-644.18) | 81.06 |
| Libya | 3.53(0.83-5.85) | 5.89(1.48-9.94) | 66.86 | 163.30(40.89-266.53) | 337.47(89.04-565.15) | 106.66 |
| Lithuania | 1.52(0.35-2.27) | 3.66(0.76-5.77) | 140.79 | 102.16(25.74-163.94) | 207.07(51.22-333.70) | 102.69 |
| Luxembourg | 4.25(0.82-6.55) | 2.27(0.46-3.57) | -46.59 | 141.93(31.52-224.17) | 166.01(39.54-274.48) | 16.97 |
| Madagascar | 8.21(0.38-14.82) | 8.65(0.29-16.35) | 5.36 | 218.36(11.73-390.97) | 251.82(12.41-473.55) | 15.32 |
| Malawi | 8.37(0.79-14.77) | 9.02(1.13-16.21) | 7.77 | 206.08(23.24-357.29) | 237.27(32.50-430.15) | 15.13 |
| Malaysia | 4.96(0.80-8.49) | 3.14(0.76-5.30) | -36.69 | 184.68(28.69-323.66) | 181.48(48.47-321.68) | -1.73 |
| Maldives | 6.81(1.80-11.31) | 3.02(0.76-5.11) | -55.65 | 211.13(55.26-354.51) | 143.63(37.73-248.08) | -31.97 |
| Mali | 10.61(1.79-17.46) | 11.55(1.79-19.93) | 8.86 | 355.98(62.18-583.17) | 466.03(80.78-794.17) | 30.91 |
| Malta | 11.18(2.99-16.67) | 6.03(1.65-8.99) | -46.06 | 276.35(80.88-406.60) | 319.36(99.03-498.35) | 15.56 |
| Marshall Islands | 28.18(2.85-50.01) | 41.06(5.17-77.56) | 45.71 | 891.36(99.58-1585.17) | 1451.00(196.04-2622.38) | 62.78 |
| Mauritania | 12.34(1.49-19.83) | 14.19(2.34-24.41) | 14.99 | 338.32(43.02-545.35) | 398.75(72.2-673.95) | 17.86 |
| Mauritius | 10.32(2.01-17.01) | 21.07(5.02-34.29) | 104.17 | 363.91(72.59-609.09) | 711.04(175.17-1203.10) | 95.39 |
| Mexico | 14.5(2.97-24.27) | 14.32(3.15-24.74) | -1.24 | 497.96(107.16-836.9) | 540.72(122.8-918.06) | 8.59 |
| Micronesia (Federated States of) | 23.12(2.60-40.52) | 29.51(4.21-54.27) | 27.64 | 698.39(84.12-1234.65) | 1001.18(144.23-1742.5) | 43.36 |
| Monaco | 1.57(0.43-2.45) | 1.62(0.43-2.51) | 3.18 | 84.41(25.75-133.27) | 156.27(45.34-256.52) | 85.13 |
| Mongolia | 2.80(0.41-4.62) | 4.02(0.73-6.40) | 43.57 | 138.11(22.04-222.49) | 253.02(51.13-402.90) | 83.20 |
| Montenegro | 4.32(0.91-6.81) | 6.48(1.39-10.73) | 50.00 | 214.72(46.69-352.12) | 318.89(76.30-524.01) | 48.51 |
| Morocco | 2.94(0.78-4.76) | 6.07(1.54-10.04) | 106.46 | 160.09(43.96-264.50) | 413.72(115.41-708.27) | 158.43 |
| Mozambique | 12.73(0.99-22.03) | 13.56(1.84-23.61) | 6.52 | 328.92(26.6-556.56) | 390.32(56.80-667.02) | 18.67 |
| Myanmar | 9.36(1.06-17.09) | 7.4(1.20-13.51) | -20.94 | 276.75(33.86-494.94) | 265.92(45.23-474.02) | -3.91 |
| Namibia | 20.85(1.81-36.18) | 16.27(1.76-30.18) | -21.97 | 526.39(45.61-906.33) | 421.60(44.94-773.68) | -19.91 |
| Nauru | 26.59(4.82-46.2) | 33.42(6.59-58.26) | 25.69 | 825.65(157.77-1406.95) | 1148.14(225.23-1977.21) | 39.06 |
| Nepal | 5.15(0.74-9.03) | 6.84(1.25-12.08) | 32.82 | 180.58(26.96-314.85) | 271.81(54.66-470.46) | 50.52 |
| Netherlands | 6.34(1.28-9.98) | 2.98(0.67-4.64) | -53.00 | 175.70(38.24-280.94) | 163.25(40.14-269.51) | -7.09 |
| New Zealand | 3.59(0.57-5.86) | 2.69(0.50-4.27) | -25.07 | 153.92(25.4-251.80) | 183.57(35.76-305.08) | 19.26 |
| Nicaragua | 6.39(0.22-11.27) | 7.8(0.87-14.37) | 22.07 | 263.66(11.43-469.86) | 387.30(44.95-690.71) | 46.89 |
| Niger | 6.07(1.09-10.56) | 7.42(1.52-13.23) | 22.24 | 196.07(36.93-326.71) | 276.87(56.79-475.97) | 41.21 |
| Nigeria | 7.44(1.82-12.44) | 8.09(2.03-13.03) | 8.74 | 200.47(49.71-329.50) | 238.87(61.61-386.01) | 19.15 |
| Niue | 19.79(2.80-35.09) | 27.22(5.15-49.12) | 37.54 | 620.62(94.31-1096.73) | 968.74(192.59-1711.31) | 56.09 |
| North Macedonia | 6.99(1.89-11.02) | 11.15(3.04-17.71) | 59.51 | 269.03(76.19-430.12) | 433.02(126.67-691.35) | 60.96 |
| Northern Mariana Islands | 14.86(3.12-25.20) | 14.03(2.99-23.55) | -5.59 | 449.14(104.29-758.74) | 519.65(122.53-863.67) | 15.70 |
| Norway | 2.75(0.63-4.17) | 2.14(0.49-3.22) | -22.18 | 141.78(35.85-224.99) | 158.40(39.25-254.67) | 11.72 |
| Oman | 7.83(1.57-13.99) | 8.59(1.40-15.39) | 9.71 | 239.71(45.87-417.21) | 303.71(57.59-551.16) | 26.70 |
| Pakistan | 10.33(2.24-16.74) | 16.26(3.67-26.91) | 57.41 | 319.36(70.33-518.77) | 560.56(134.41-882.98) | 75.53 |
| Palau | 19.37(3.02-33.79) | 23.44(4.70-41.06) | 21.01 | 618.19(95.38-1052.10) | 862.56(187.02-1500.91) | 39.53 |
| Palestine | 15.44(2.76-26.55) | 12.47(3.31-20.35) | -19.24 | 393.35(70.61-655.01) | 409.52(114.16-674.20) | 4.11 |
| Panama | 3.87(0.32-7.06) | 4.93(0.60-9.12) | 27.39 | 154.74(14.45-284.88) | 240.15(29.78-436.15) | 55.20 |
| Papua New Guinea | 20.45(4.06-36.50) | 20.13(3.64-35.67) | -1.56 | 598.94(120.45-1047.05) | 696.12(137.43-1189.21) | 16.23 |
| Paraguay | 5.49(0.17-9.93) | 11.65(0.68-21.96) | 112.20 | 192.90(9.14-352.45) | 392.45(25.81-736.28) | 103.45 |
| Peru | 2.09(0.44-3.64) | 2.39(0.63-4.44) | 14.35 | 73.51(16.74-127.85) | 100.09(28.98-178.18) | 36.16 |
| Philippines | 3.90(0.74-6.87) | 6.40(1.25-10.98) | 64.10 | 132.99(25.77-233.81) | 226.06(45.97-389.20) | 69.98 |
| Poland | 4.83(0.99-7.59) | 4.61(1.00-7.29) | -4.55 | 239.40(52.62-381.42) | 275.25(64.99-443.54) | 14.97 |
| Portugal | 6.59(1.26-10.77) | 4.34(0.79-7.07) | -34.14 | 203.62(44.22-336.48) | 262.89(55.06-446.94) | 29.11 |
| Puerto Rico | 14.05(1.53-23.71) | 9.13(1.34-15.89) | -35.02 | 446.49(59.75-770.98) | 438.78(84.98-765.10) | -1.73 |
| Qatar | 22.88(6.27-37.28) | 21.28(5.60-35.50) | -6.99 | 591.29(166.08-960.38) | 734.68(215.49-1192.10) | 24.25 |
| Republic of Korea | 3.89(0.68-6.69) | 2.55(0.42-4.27) | -34.45 | 153.50(29.47-264.88) | 247.09(46.38-442.42) | 60.97 |
| Republic of Moldova | 2.08(0.45-3.30) | 2.65(0.59-4.23) | 27.40 | 120.97(28.44-201.80) | 191.57(44.84-329.56) | 58.36 |
| Romania | 2.33(0.54-3.69) | 2.55(0.55-4.06) | 9.44 | 126.22(30.42-212.92) | 181.84(48.13-309.28) | 44.07 |
| Russian Federation | 1.67(0.35-2.50) | 5.91(1.34-9.12) | 253.89 | 112.25(25.63-176.48) | 241.05(58.29-381.13) | 114.74 |
| Rwanda | 14.81(0.30-27.74) | 7.65(1.28-15.31) | -48.35 | 381.63(11.29-715.20) | 192.51(39.12-364.43) | -49.56 |
| Saint Kitts and Nevis | 19.17(4.15-31.14) | 12.85(2.65-21.38) | -32.97 | 581.89(131.61-977.40) | 512.05(116.00-880.77) | -12.00 |
| Saint Lucia | 27.87(4.44-46.52) | 14.73(2.62-25.33) | -47.15 | 816.62(137.80-1372.14) | 609.39(120.42-1061.54) | -25.38 |
| Saint Vincent and the Grenadines | 27.43(4.54-45.50) | 14.89(3.66-25.15) | -45.72 | 777.97(140.54-1279.85) | 583.45(159.46-1004.03) | -25.00 |
| Samoa | 26.37(1.78-44.56) | 31.78(4.25-54.07) | 20.52 | 825.06(58.20-1400.68) | 1180.33(162.55-1990.12) | 43.06 |
| San Marino | 3.18(0.69-4.92) | 1.53(0.40-2.56) | -51.89 | 114.17(28.42-177.14) | 156.07(41.14-260.41) | 36.70 |
| Sao Tome and Principe | 3.68(0.64-6.30) | 4.52(0.88-7.46) | 22.83 | 148.61(25.32-252.26) | 225.76(53.11-390.17) | 51.91 |
| Saudi Arabia | 4.91(1.41-8.06) | 7.36(2.43-11.89) | 49.90 | 189.98(58.3-307.64) | 375.56(130.53-599.29) | 97.68 |
| Senegal | 8.48(1.86-13.92) | 10.97(2.41-18.62) | 29.36 | 272.31(61.72-446.17) | 386.30(84.45-644.42) | 41.86 |
| Serbia | 6.51(1.72-10.25) | 6.38(1.69-10.38) | -2.00 | 246.24(68.26-391.09) | 309.13(83.56-511.23) | 25.54 |
| Seychelles | 2.08(0.19-3.73) | 2.50(0.37-4.54) | 20.19 | 87.12(9.31-162.49) | 159.18(26.15-305.59) | 82.71 |
| Sierra Leone | 4.88(0.83-8.49) | 6.56(1.21-11.25) | 34.43 | 146.52(26.84-253.41) | 231.97(46.47-405.37) | 58.32 |
| Singapore | 3.06(0.42-5.49) | 0.43(0.07-0.74) | -85.95 | 151.72(21.79-283.32) | 166.93(31.42-308.72) | 10.03 |
| Slovakia | 3.85(0.79-6.21) | 3.12(0.69-5.03) | -18.96 | 172.18(37.51-284.16) | 205.23(46.54-333.26) | 19.20 |
| Slovenia | 3.75(0.70-5.99) | 2.97(0.64-4.66) | -20.80 | 178.15(35.15-296.81) | 194.86(47.3-324.74) | 9.38 |
| Solomon Islands | 25.31(1.23-47.36) | 30.85(0.58-56.10) | 21.89 | 722.60(37.31-1331.42) | 967.48(27.26-1734.45) | 33.89 |
| Somalia | 17.37(0.63-31.28) | 18.61(1.28-33.44) | 7.14 | 458.58(18.22-799.37) | 523.90(39.03-912.88) | 14.24 |
| South Africa | 8.99(1.30-15.23) | 17.96(2.83-30.31) | 99.78 | 267.17(37.74-452.06) | 524.35(86.16-886.94) | 96.26 |
| South Sudan | 9.33(1.39-17.35) | 12.02(2.14-21.92) | 28.83 | 239.02(38.33-436.43) | 327.12(62.48-596.70) | 36.86 |
| Spain | 6.92(1.56-10.64) | 2.62(0.63-4.14) | -62.14 | 244.82(59.95-394.85) | 242.16(61.49-411.12) | -1.09 |
| Sri Lanka | 6.45(1.02-11.39) | 6.43(1.33-11.46) | -0.31 | 195.74(31.53-351.66) | 270.62(67.01-474.84) | 38.25 |
| Sudan | 3.02(0.75-5.09) | 4.43(1.09-7.70) | 46.69 | 128.70(33.87-210.35) | 237.53(65.73-400.99) | 84.56 |
| Suriname | 5.75(1.53-9.40) | 6.37(1.80-11.11) | 10.78 | 221.61(62.42-367.48) | 346.61(105.36-590.48) | 56.41 |
| Sweden | 3.48(0.74-5.33) | 2.97(0.70-4.60) | -14.66 | 135.54(30.61-217.43) | 175.93(44.93-282.29) | 29.80 |
| Switzerland | 5.38(1.00-8.34) | 2.12(0.44-3.30) | -60.59 | 191.04(40.84-309.90) | 234.58(55.15-388.94) | 22.79 |
| Syrian Arab Republic | 4.29(1.06-7.05) | 4.82(1.27-8.11) | 12.35 | 168.29(44.9-278.03) | 266.08(75.52-455.23) | 58.11 |
| Taiwan (Province of China) | 6.7(0.71-11.85) | 5.65(0.71-9.71) | -15.67 | 205.09(22.55-368.26) | 252.30(35.08-448.75) | 23.02 |
| Tajikistan | 4.27(1.06-6.67) | 4.83(1.20-7.97) | 13.11 | 164.23(42.91-254.58) | 244.92(65.89-399.38) | 49.13 |
| Thailand | 2.56(0.38-4.66) | 2.57(0.65-4.57) | 0.39 | 84.42(12.91-151.12) | 122.90(33.74-218.79) | 45.58 |
| Timor-Leste | 3.98(0.08-7.62) | 4.32(0.23-8.33) | 8.54 | 124.75(2.86-231.78) | 197.02(11.81-373.50) | 57.93 |
| Togo | 7.05(1.09-12.30) | 10.49(1.63-18.36) | 48.79 | 198.07(33.36-330.72) | 318.03(49.95-543.18) | 60.56 |
| Tokelau | 19.73(2.49-35.55) | 19.66(3.11-34.8) | -0.35 | 650.86(88.63-1134.23) | 805.96(142.47-1434.09) | 23.83 |
| Tonga | 23.10(3.24-39.46) | 26.28(3.64-47.31) | 13.77 | 688.94(102.58-1189.92) | 875.69(125.84-1557.16) | 27.11 |
| Trinidad and Tobago | 33.37(8.51-53.37) | 25.72(6.48-44.54) | -22.92 | 1000.11(272.52-1597.98) | 947.99(271.66-1595.52) | -5.21 |
| Tunisia | 2.58(0.70-4.28) | 4.17(1.09-7.10) | 61.63 | 125.69(34.88-208.27) | 288.25(90.19-493.13) | 129.33 |
| Turkey | 9.63(2.89-15.42) | 7.49(2.30-11.80) | -22.22 | 257.67(75.25-408.97) | 312.95(103.98-504.31) | 21.45 |
| Turkmenistan | 3.97(0.83-6.15) | 9.11(2.20-15.11) | 129.47 | 157.05(35.62-247.60) | 386.19(96.58-615.99) | 145.90 |
| Tuvalu | 24.04(2.67-41.74) | 25.09(3.40-44.09) | 4.37 | 701.86(78.21-1206.27) | 822.87(122.27-1438.13) | 17.24 |
| Uganda | 8.56(-0.06-17.89) | 8.03(0.49-15.85) | -6.19 | 213.57(1.69-431.90) | 212.65(19.42-403.12) | -0.43 |
| Ukraine | 1.26(0.24-1.99) | 0.99(0.22-1.69) | -21.43 | 93.30(19.1-152.27) | 131.34(30.11-216.95) | 40.77 |
| United Arab Emirates | 14.00(3.51-22.73) | 11.23(2.90-18.41) | -19.79 | 442.19(110.64-700.96) | 401.16(111.13-654.42) | -9.28 |
| United Kingdom | 3.46(0.82-5.33) | 1.64(0.40-2.52) | -52.60 | 137.52(34.67-215.78) | 222.60(59.56-369.70) | 61.87 |
| United Republic of Tanzania | 6.90(0.87-12.12) | 5.53(0.9-10.02) | -19.86 | 172.37(22.27-303.66) | 153.85(28.09-273.73) | -10.74 |
| United States of America | 5.42(1.17-8.41) | 4.78(1.22-7.32) | -11.81 | 217.72(49.39-345.99) | 371.91(101.84-602.74) | 70.82 |
| United States Virgin Islands | 8.54(1.92-14.2) | 5.97(1.46-10.47) | -30.09 | 321.33(78.84-541.74) | 428.24(119.23-753.04) | 33.27 |
| Uruguay | 7.16(1.48-10.88) | 7.15(1.87-10.59) | -0.14 | 219.98(49.62-339.38) | 335.60(92.28-535.05) | 52.56 |
| Uzbekistan | 3.70(0.87-5.58) | 9.60(2.14-15.52) | 159.46 | 165.41(41.90-262.24) | 435.52(107.17-701.02) | 163.3 |
| Vanuatu | 16.06(1.7-30.62) | 20.36(2.71-35.85) | 26.77 | 474.84(51.46-886.51) | 704.25(90.30-1220.44) | 48.31 |
| Venezuela (Bolivarian Republic of) | 5.74(0.92-9.99) | 7.49(1.29-13.63) | 30.49 | 203.35(35.71-355.02) | 294.74(56.26-517.36) | 44.94 |
| Viet Nam | 3.57(0.34-6.47) | 4.28(0.14-8.32) | 19.89 | 96.95(9.30-173.46) | 139.41(3.83-268.64) | 43.80 |
| Yemen | 3.4(0.89-5.99) | 4.11(0.97-7.49) | 20.88 | 135.15(36.7-232.29) | 217.16(53.01-372.67) | 60.68 |
| Zambia | 11.08(1.09-19.15) | 11.26(1.12-20.47) | 1.62 | 299.08(32.13-517.81) | 351.59(36.91-623.98) | 17.56 |
| Zimbabwe | 10.62(0.11-19.21) | 18.83(0.36-34.33) | 77.31 | 275.90(4.3-486.39) | 532.34(13.26-949.58) | 92.95 |

Note: ASR: age-standardized rates; DALY, disability-adjusted life years; UI, uncertainty interval


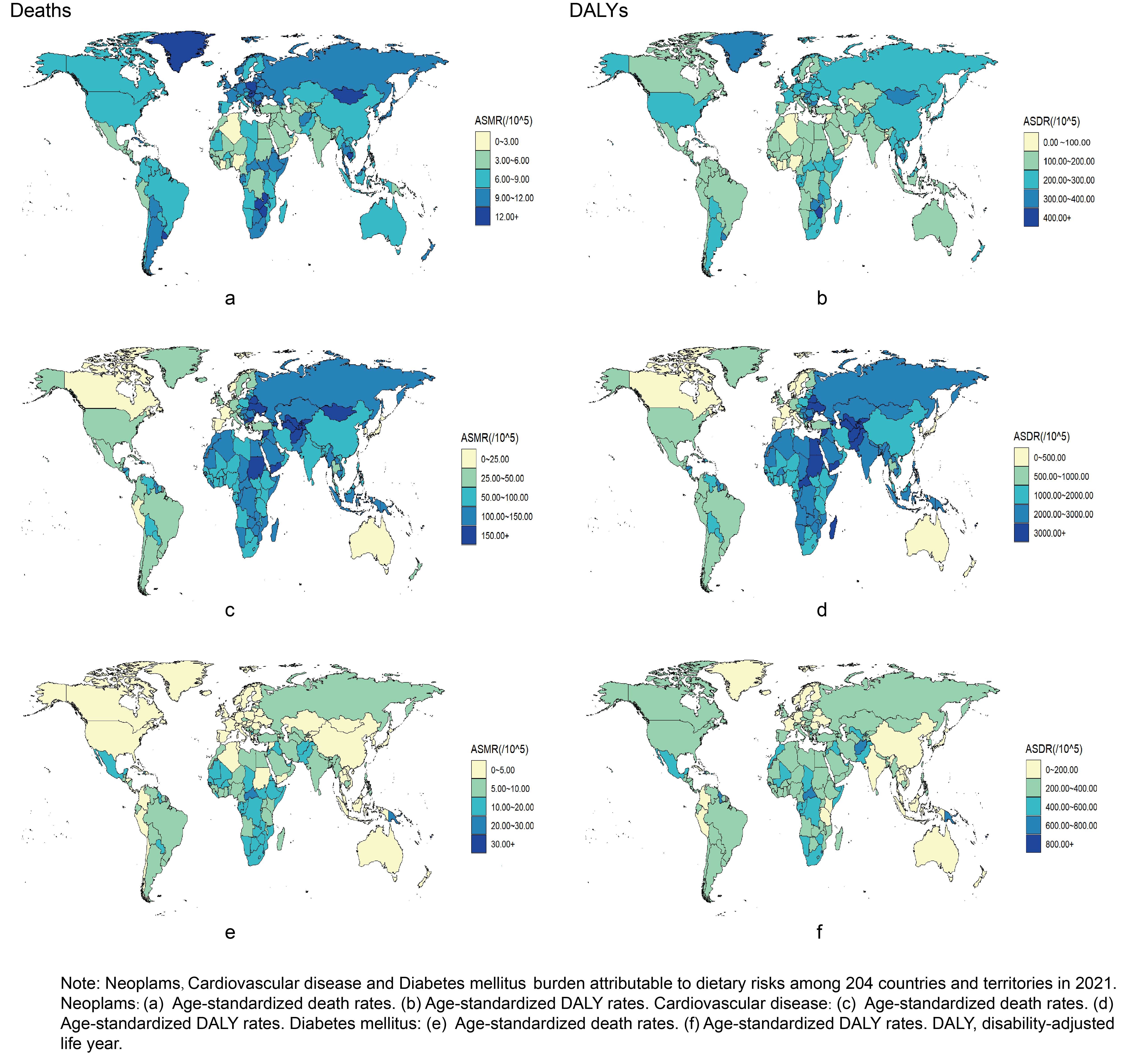


# Figure S1 Burden of chronic diseases attributed to dietary risks in 204 countries and regions in 2021

# Note: Neoplasms, Cardiovascular disease and Diabetes mellitus burdens attributable to dietary risks among 204 countries and territories in 2021. Neoplasms: (a) Age-standardized death rates. (b) Age-standardized DALY rates. Cardiovascular disease: (c) Age-standardized death rates. (d) Age-standardized DALY rates. Diabetes mellitus: (e) Age-standardized death rates. (f) Age-standardized DALY rates. DALY, disability-adjusted life year.

# Table S4 Countries and regions with an average annual decline in mortality and disability-adjusted life years attributed to dietary factors for three chronic diseases from 1990 to 2021

| **Countries or territories** | **Neoplasms** | **Cardiovascular disease** | **Diabetes mellitus** |
| --- | --- | --- | --- |
|  | **Percentage changes(%)** | **Percentage changes(%)** | **Percentage changes(%)** |
| **Death** |  |  |  |
| Andorra | -36.07 | -52.10 | -31.89 |
| Angola | -18.97 | -31.95 | -39.13 |
| Argentina | -20.93 | -60.21 | -31.20 |
| Armenia | -26.04 | -39.88 | -15.93 |
| Australia | -39.34 | -75.14 | -22.78 |
| Austria | -51.09 | -58.59 | -32.04 |
| Belarus | -19.26 | -10.28 | -1.73 |
| Belgium | -45.33 | -72.96 | -51.78 |
| Bermuda | -38.41 | -65.68 | -46.69 |
| Botswana | -13.66 | -35.16 | -6.35 |
| Brazil | -9.25 | -60.17 | -5.92 |
| Brunei Darussalam | -28.31 | -43.40 | -35.33 |
| Burundi | -34.49 | -36.72 | -11.71 |
| Canada | -33.92 | -69.3 | -36.43 |
| Chile | -26.88 | -65.22 | -30.49 |
| Colombia | -22.82 | -56.5 | -24.78 |
| Congo | -21.58 | -24.19 | -4.65 |
| Cook Islands | -16.80 | -49.70 | -24.09 |
| Cyprus | -32.27 | -65.51 | -64.27 |
| Equatorial Guinea | -17.30 | -50.82 | -12.40 |
| Ethiopia | -36.88 | -49.93 | -50.90 |
| Finland | -31.81 | -62.87 | -39.06 |
| France | -37.47 | -62.44 | -20.26 |
| Germany | -40.09 | -63.91 | -35.45 |
| Greece | -16.14 | -47.17 | -20.50 |
| Greenland | -44.87 | -62.16 | -36.99 |
| Guam | -35.29 | -50.80 | -51.50 |
| Iceland | -35.98 | -68.29 | -34.43 |
| Ireland | -48.54 | -80.63 | -55.72 |
| Israel | -39.30 | -80.87 | -7.33 |
| Italy | -34.20 | -52.26 | -46.87 |
| Japan | -26.51 | -66.91 | -67.37 |
| Jordan | -20.10 | -50.42 | -27.90 |
| Lao People's Democratic Republic | -27.28 | -52.88 | -54.22 |
| Lebanon | -15.08 | -61.72 | -19.31 |
| Luxembourg | -46.67 | -71.60 | -46.59 |
| Malaysia | -5.14 | -38.19 | -36.69 |
| Maldives | -55.04 | -65.46 | -55.65 |
| Malta | -36.72 | -67.87 | -46.06 |
| Myanmar | -27.26 | -51.43 | -20.94 |
| Netherlands | -21.82 | -71.27 | -53.00 |
| New Zealand | -39.60 | -68.04 | -25.07 |
| Norway | -28.41 | -78.07 | -22.18 |
| Palestine | -18.89 | -44.70 | -19.24 |
| Papua New Guinea | -15.89 | -7.87 | -1.56 |
| Poland | -7.79 | -61.56 | -4.55 |
| Portugal | -33.43 | -66.97 | -34.14 |
| Puerto Rico | -25.81 | -65.45 | -35.02 |
| Qatar | -29.17 | -73.66 | -6.99 |
| Republic of Korea | -38.52 | -71.62 | -34.45 |
| Rwanda | -32.09 | -56.47 | -48.35 |
| Saint Kitts and Nevis | -23.97 | -52.67 | -32.97 |
| Saint Lucia | -27.43 | -57.86 | -47.15 |
| Saint Vincent and the Grenadines | -16.80 | -37.46 | -45.72 |
| San Marino | -54.12 | -67.36 | -51.89 |
| Serbia | -17.93 | -48.38 | -2.00 |
| Singapore | -49.50 | -73.37 | -85.95 |
| Slovakia | -16.57 | -48.99 | -18.96 |
| Slovenia | -32.21 | -62.01 | -20.80 |
| Spain | -25.75 | -61.63 | -62.14 |
| Sri Lanka | -29.55 | -48.35 | -0.31 |
| Sweden | -27.87 | -69.70 | -14.66 |
| Switzerland | -36.14 | -67.04 | -60.59 |
| Tokelau | -15.54 | -30.23 | -0.35 |
| Trinidad and Tobago | -16.48 | -57.63 | -22.92 |
| Turkey | -23.13 | -48.19 | -22.22 |
| United Arab Emirates | -18.39 | -29.03 | -19.79 |
| United Kingdom | -38.97 | -73.32 | -52.60 |
| United Republic of Tanzania | -12.27 | -16.85 | -19.86 |
| United States of America | -37.39 | -51.12 | -11.81 |
| United States Virgin Islands | -36.20 | -58.10 | -30.09 |
| Uruguay | -11.22 | -57.50 | -0.14 |
| **DALY** |  |  |  |
| Angola | -21.20 | -38.49 | -29.17 |
| Barbados | -2.87 | -44.16 | -12.13 |
| Burundi | -38.48 | -39.77 | -7.75 |
| Cuba | -1.49 | -49.82 | -1.48 |
| Cyprus | -29.71 | -63.85 | -44.60 |
| Equatorial Guinea | -21.32 | -55.91 | -1.25 |
| Ethiopia | -43.10 | -54.16 | -47.75 |
| Indonesia | -5.66 | -18.49 | -6.94 |
| Ireland | -49.84 | -81.92 | -1.38 |
| Italy | -36.63 | -57.76 | -13.74 |
| Lao People's Democratic Republic | -30.46 | -58.45 | -42.07 |
| Malaysia | -7.27 | -39.81 | -1.73 |
| Maldives | -59.27 | -70.84 | -31.97 |
| Myanmar | -30.50 | -56.41 | -3.91 |
| Netherlands | -23.89 | -74.79 | -7.09 |
| Puerto Rico | -21.60 | -61.76 | -1.73 |
| Rwanda | -38.30 | -61.86 | -49.56 |
| Saint Kitts and Nevis | -29.25 | -56.54 | -12.00 |
| Saint Lucia | -24.39 | -56.06 | -25.38 |
| Saint Vincent and the Grenadines | -10.63 | -41.48 | -25.00 |
| Spain | -28.19 | -60.89 | -1.09 |
| Trinidad and Tobago | -6.87 | -53.68 | -5.21 |
| United Arab Emirates | -30.60 | -41.94 | -9.28 |
| United Republic of Tanzania | -15.91 | -20.19 | -10.74 |

Note: DALY, disability-adjusted life years

# Table S5 Countries and regions with an average annual increasing in mortality and disability-adjusted life years attributed to dietary factors for three chronic diseases from 1990 to 2021

| **Countries or territories** | **Neoplasms** | **Cardiovascular disease** | **Diabetes mellitus** |
| --- | --- | --- | --- |
|  | **Percentage changes(%)** | **Percentage changes(%)** | **Percentage changes(%)** |
| **Death** |  |  |  |
| American Samoa | 9.57 | 0.66 | 23.73 |
| Cabo Verde | 32.07 | 4.10 | 198.28 |
| Chad | 40.84 | 10.41 | 57.67 |
| Democratic Republic of the Congo | 0.55 | 6.04 | 11.04 |
| Gambia | 9.54 | 14.51 | 54.60 |
| Guinea | 22.57 | 10.93 | 59.08 |
| Honduras | 38.24 | 26.51 | 91.91 |
| Kenya | 37.06 | 9.92 | 34.65 |
| Lesotho | 83.61 | 45.28 | 132.77 |
| Liberia | 13.76 | 9.66 | 56.15 |
| Libya | 8.49 | 21.27 | 66.86 |
| Montenegro | 16.51 | 12.90 | 50.00 |
| Pakistan | 17.41 | 17.84 | 57.41 |
| South Africa | 16.75 | 6.29 | 99.78 |
| Togo | 28.95 | 1.08 | 48.79 |
| Zimbabwe | 28.30 | 36.54 | 77.31 |
| **DALY** |  |  |  |
| American Samoa | 13.41 | 1.46 | 53.79 |
| Burkina Faso | 10.87 | 0.09 | 24.23 |
| Chad | 40.37 | 10.05 | 73.38 |
| Gambia | 9.97 | 10.66 | 67.33 |
| Guinea | 22.26 | 9.74 | 74.49 |
| Honduras | 27.76 | 13.29 | 92.67 |
| Kenya | 31.20 | 5.12 | 41.46 |
| Lesotho | 92.49 | 55.88 | 153.41 |
| Liberia | 17.62 | 8.92 | 81.06 |
| Libya | 10.06 | 19.90 | 106.66 |
| Nauru | 2.40 | 0.69 | 39.06 |
| Northern Mariana Islands | 0.91 | 0.06 | 15.70 |
| Pakistan | 17.75 | 15.73 | 75.53 |
| Zimbabwe | 38.05 | 50.21 | 92.95 |

Note: DALY, disability-adjusted life years

# Table S6 Mortality and disability-adjusted life years attributable to dietary factors among patients with chronic diseases in 5 SDI and 21 GBD regions from 1990 to 2021

| **location** | **ASR of Deaths (per 100,000 population)** | | | **ASR of DALYs (per 100,000 population)** | | |
| --- | --- | --- | --- | --- | --- | --- |
|  | **1990(95%UI)** | **2021(95%UI)** | **Percentage changes(%)** | **1990(95%UI)** | **2021(95%UI)** | **Percentage changes(%)** |
| **Neoplasms** |  |  |  |  |  |  |
| **SDI regions** |  |  |  |  |  |  |
| Low SDI | 6.61 (2.53 - 11.54) | 5.81 (2.25 - 9.60) | -12.19 | 177.45 (60.07 - 310.14) | 150.04 (49.71 - 253.66) | -15.45 |
| Low-middle SDI | 5.30 (2.06 - 9.75) | 5.23 (1.94 – 9.00) | -1.36 | 143.02 (51.42 - 265.59) | 139.67 (46.84 - 242.91) | -2.34 |
| Middle SDI | 11.93 (3.11 - 23.4) | 7.22 (2.58 - 13.46) | -39.44 | 309.73 (77.77 - 611.05) | 180.58 (60.55 - 336.14) | -41.70 |
| High-middle SDI | 14.79 (3.70 - 28.86) | 9.16 (2.87 - 16.89) | -38.04 | 378.29 (91.34 - 748.07) | 219.57 (67.29 - 406.47) | -41.96 |
| High SDI | 13.81 (3.55 - 24.16) | 8.89 (2.23 - 15.14) | -35.61 | 328.52 (81.48 - 586.04) | 207.54 (47.33 - 352.53) | -36.83 |
| **GBD regions** |  |  |  |  |  |  |
| Andean Latin America | 7.48 (2.63 - 19.49) | 6.53 (2.18 - 15.09) | -12.72 | 182.82 (57.88 - 473.70) | 156.76 (47.39 - 363.24) | -14.26 |
| Australasia | 14.15 (3.38 - 23.93) | 8.56 (1.89 - 14.34) | -39.55 | 341.18 (76.24 - 579.20) | 199.41 (41.75 - 342.84) | -41.55 |
| Caribbean | 8.54 (3.02 - 15.24) | 7.94 (2.89 - 13.28) | -7.01 | 209.38 (68.23 - 382.84) | 198.15 (65.72 - 336.61) | -5.36 |
| Central Asia | 11.73 (2.44 - 23.43) | 5.76 (1.44 - 11.34) | -50.94 | 322.26 (67.66 - 645.06) | 149.44 (36.27 - 296.26) | -53.63 |
| Central Europe | 13.40 (3.81 - 24.39) | 12.13 (3.28 - 20.29) | -9.52 | 330.41 (91.63 - 603.33) | 282.90 (73.39 - 477.4) | -14.38 |
| Central Latin America | 6.02 (1.59 - 14.33) | 5.70 (1.66 - 11.5) | -5.38 | 145.00 (35.33 - 343.55) | 147.76 (40.6 - 294.02) | 1.90 |
| Central Sub-Saharan Africa | 6.77 (2.68 - 11.30) | 6.26 (2.81 - 10.06) | -7.59 | 185.94 (58.73 - 323.17) | 167.75 (61.53 - 276.15) | -9.78 |
| East Asia | 18.13 (3.86 - 37.29) | 8.74 (3.01 - 17.81) | -51.8 | 462.14 (96.32 - 959.07) | 212.63 (71.30 - 431.73) | -53.99 |
| Eastern Europe | 14.10 (3.79 - 28.06) | 10.84 (2.84 - 19.01) | -23.13 | 379.00 (98.71 - 762.46) | 267.79 (67.71 - 474.80) | -29.34 |
| Eastern Sub-Saharan Africa | 11.33 (3.56 - 18.23) | 9.88 (2.93 - 15.69) | -12.83 | 295.73 (86.46 - 483.91) | 239.89 (61.55 - 387.71) | -18.88 |
| High-income Asia Pacific | 12.35 (3.44 - 28.08) | 8.53 (2.55 - 15.72) | -30.91 | 297.45 (78.42 - 679.23) | 194.47 (56.39 - 356.77) | -34.62 |
| High-income North America | 12.78 (2.96 - 21.53) | 8.06 (1.61 - 13.60) | -36.95 | 313.09 (67.98 - 535.47) | 200.96 (37.31 - 345.12) | -35.81 |
| North Africa and Middle East | 5.31 (1.79 - 10.49) | 4.84 (1.57 - 8.62) | -8.86 | 136.60 (42.78 - 274.45) | 120.95 (36.64 - 218.53) | -11.46 |
| Oceania | 6.13 (2.44 - 13.16) | 5.19 (1.92 - 10.77) | -15.43 | 165.43 (57.77 - 354.16) | 145.69 (51.89 - 302.24) | -11.93 |
| South Asia | 4.42 (1.52 - 8.02) | 4.21 (1.48 - 7.20) | -4.85 | 120.31 (39.49 - 219.67) | 112.31 (36.66 - 193.32) | -6.64 |
| Southeast Asia | 8.41 (4.62 - 13.52) | 8.16 (4.23 - 12.43) | -3.00 | 224.58 (113.04 - 363.86) | 211.69 (100.22 - 334.64) | -5.74 |
| Southern Latin America | 14.12 (3.64 - 25.87) | 10.84 (2.91 - 18.68) | -23.22 | 330.80 (82.46 - 611.70) | 251.76 (64.38 - 434.85) | -23.89 |
| Southern Sub-Saharan Africa | 9.97 (2.96 - 16.15) | 11.74 (3.52 - 19.17) | 17.79 | 260.60 (69.39 - 432.43) | 297.88 (83.75 - 492.82) | 14.30 |
| Tropical Latin America | 8.34 (1.73 - 17.34) | 7.64 (1.70 - 13.73) | -8.46 | 208.25 (39.37 - 433.88) | 197.23 (42.15 - 356.50) | -5.29 |
| Western Europe | 14.28 (3.31 - 25.02) | 9.07 (1.84 - 15.5) | -36.50 | 331.01 (72.52 - 583.72) | 206.89 (39.2 - 357.56) | -37.50 |
| Western Sub-Saharan Africa | 3.00 (0.39 - 5.58) | 3.53 (0.69 - 6.27) | 17.86 | 87.98 (29.19 - 156.23) | 103.80 (34.1 - 183.58) | 17.98 |
| **Cardiovascular disease** |  |  |  |  |  |  |
| **SDI regions** |  |  |  |  |  |  |
| Low SDI | 116.45 (35.01 - 168.73) | 96.81 (30.01 - 139.76) | -16.87 | 2744.59 (753.19 - 3910.52) | 2180.08 (606.6 - 3116.26) | -20.57 |
| Low-middle SDI | 116.10 (26.92 - 168.71) | 95.95 (18.16 - 143.76) | -17.35 | 2816.47 (583.35 - 4024.54) | 2260.27 (363.47 - 3324.90) | -19.75 |
| Middle SDI | 120.94 (52.04 - 168.98) | 78.74 (25.80 - 118.50) | -34.89 | 2654.84 (1123.96 - 3616.56) | 1695.16 (471.77 - 2454.79) | -36.15 |
| High-middle SDI | 136.06 (32.98 - 198.86) | 75.95 (18.69 - 114) | -44.18 | 2809.86 (753.73 - 3930.95) | 1516.27 (385.06 - 2215.68) | -46.04 |
| High SDI | 83.61 (15.48 - 126.75) | 32.12 (9.77 - 47.84) | -61.58 | 1700.34 (345.50 - 2473.78) | 704.13 (226.17 - 999.13) | -58.59 |
| **GBD regions** |  |  |  |  |  |  |
| Andean Latin America | 64.66 (20.46 - 93.89) | 34.53 (9.87 - 53.69) | -46.6 | 1382.32 (415.26 - 1974.71) | 722.84 (183.02 - 1101.45) | -47.71 |
| Australasia | 78.42 (-12.48 - 132.54) | 20.38 (-1.41 - 33.69) | -74.01 | 1552.72 (-257.84 - 2496.76) | 398.70 (-28.15 - 622.16) | -74.32 |
| Caribbean | 101.55 (20.77 - 155.88) | 60.04 (19.36 - 91.02) | -40.87 | 2203.25 (440.35 - 3297.62) | 1397.93 (454.29 - 2085.38) | -36.55 |
| Central Asia | 216.78 (40.58 - 311.31) | 163.72 (17.45 - 244.87) | -24.48 | 4537.62 (760.82 - 6289.97) | 3199.10 (308.38 - 4689.70) | -29.50 |
| Central Europe | 203.90 (73.89 - 284.63) | 99.86 (38.93 - 143.91) | -51.02 | 4089.97 (1325.36 - 5565.83) | 1854.04 (676.31 - 2625.75) | -54.67 |
| Central Latin America | 74.48 (19.10 - 110.20) | 50.81 (9.47 - 78.13) | -31.77 | 1539.96 (389.66 - 2228.04) | 1065.52 (194.34 - 1595.40) | -30.81 |
| Central Sub-Saharan Africa | 119.67 (28.53 - 184.66) | 111.39 (31.22 - 169.16) | -6.92 | 2677.62 (558.81 - 4150.02) | 2355.75 (577.50 - 3600.92) | -12.02 |
| East Asia | 126.79 (69.05 - 178.77) | 76.89 (30.43 - 119.06) | -39.36 | 2599.69 (1454.63 - 3578.4) | 1493.76 (635.02 - 2227.26) | -42.54 |
| Eastern Europe | 195.25 (16.59 - 298.33) | 132.36 (2.67 - 212.34) | -32.21 | 4039.58 (500.35 - 5814.53) | 2718.47 (108.49 - 4168.11) | -32.7 |
| Eastern Sub-Saharan Africa | 118.93 (61.05 - 164.22) | 84.49 (34.83 - 122.20) | -28.96 | 2706.28 (1263.86 - 3721.45) | 1830.02 (675.86 - 2644.59) | -32.38 |
| High-income Asia Pacific | 50.55 (20.02 - 76.97) | 15.99 (6.29 - 24.80) | -68.36 | 992.91 (422.23 - 1471.13) | 352.13 (140.11 - 527.71) | -64.54 |
| High-income North America | 81.03 (19.69 - 123.56) | 38.13 (16.35 - 54.07) | -52.94 | 1695.66 (442.94 - 2461.16) | 869.57 (404.99 - 1171.73) | -48.72 |
| North Africa and Middle East | 164.94 (19.52 - 250.90) | 101.67 (8.74 - 164.31) | -38.36 | 3696.62 (345.76 - 5453.95) | 2195.02 (123.74 - 3491.65) | -40.62 |
| Oceania | 142.78 (46.31 - 212.28) | 119.76 (27.5 - 183.01) | -16.12 | 3455.38 (1097.59 - 5126.43) | 2936.67 (635.87 - 4436.32) | -15.01 |
| South Asia | 107.07 (17.58 - 160.26) | 98.54 (15.13 - 148.72) | -7.97 | 2699.21 (374.49 - 3942.77) | 2371.53 (303.34 - 3499.9) | -12.14 |
| Southeast Asia | 119.35 (42.1 - 176.63) | 80.65 (19.08 - 127.37) | -32.43 | 2881.85 (951.70 - 4178.82) | 1883.77 (388.18 - 2934.24) | -34.63 |
| Southern Latin America | 102.45 (29.6 - 143.26) | 38.92 (14.72 - 54.81) | -62.01 | 2049.20 (633.57 - 2752.43) | 777.91 (261.46 - 1064.71) | -62.04 |
| Southern Sub-Saharan Africa | 71.41 (26.94 – 100.00) | 77.07 (33.73 - 105.95) | 7.92 | 1669.55 (588.29 - 2274.88) | 1679.48 (685.10 - 2282.81) | 0.59 |
| Tropical Latin America | 86.79 (43.40 - 123.49) | 35.08 (18.35 - 49.87) | -59.59 | 1971.90 (1055.93 - 2702.63) | 812.66 (434.75 - 1115.37) | -58.79 |
| Western Europe | 74.98 (6.08 - 116.98) | 25.67 (6.06 - 38.99) | -65.77 | 1484.82 (123.55 - 2216.76) | 487.56 (103.94 - 711.25) | -67.16 |
| Western Sub-Saharan Africa | 87.10 (20.96 - 136.91) | 70.03 (13.4 - 109.94) | -19.60 | 1935.18 (448.56 - 3004.54) | 1519.47 (290.77 - 2356.01) | -21.48 |
| **Diabetes mellitus** |  |  |  |  |  |  |
| **SDI regions** |  |  |  |  |  |  |
| Low SDI | 8.91 (1.2 - 14.89) | 8.68 (1.33 - 14.53) | -2.54 | 260.26 (37.77 - 436.8) | 292.19 (50.31 - 492.72) | 12.27 |
| Low-middle SDI | 5.47 (0.91 - 9.28) | 7.01 (1.34 - 11.53) | 28.23 | 169.33 (30.76 - 288.55) | 254.14 (55.93 - 422.80) | 50.09 |
| Middle SDI | 3.81 (0.65 - 6.49) | 4.43 (0.78 - 7.49) | 16.18 | 138.06 (23.96 - 237.56) | 197.22 (39.52 - 337.88) | 42.85 |
| High-middle SDI | 3.92 (0.85 - 6.15) | 3.57 (0.74 - 5.74) | -8.89 | 147.92 (32.67 - 240.25) | 192.42 (41.84 - 324.99) | 30.08 |
| High SDI | 4.34 (0.88 - 6.82) | 3.16 (0.72 - 4.94) | -27.05 | 162.69 (35.89 - 261.87) | 247.38 (62.69 - 408.23) | 52.06 |
| **GBD regions** |  |  |  |  |  |  |
| Andean Latin America | 3.93 (0.75 - 6.63) | 4.51 (0.82 - 7.91) | 14.66 | 134.57 (28.30 - 232.81) | 192.80 (41.00 - 334.98) | 43.26 |
| Australasia | 4.07 (0.72 - 6.56) | 3.15 (0.61 - 4.94) | -22.66 | 138.34 (25.94 - 226.59) | 169.44 (35.79 - 286.11) | 22.48 |
| Caribbean | 9.63 (1.80 - 15.88) | 7.60 (1.32 - 13.04) | -21.09 | 320.18 (64.30 - 535.07) | 358.72 (72.30 - 623.26) | 12.04 |
| Central Asia | 3.56 (0.82 - 5.39) | 6.54 (1.52 - 10.24) | 83.53 | 168.73 (41.77 - 267.09) | 343.63 (89.24 - 548.12) | 103.65 |
| Central Europe | 4.27 (0.94 - 6.65) | 4.64 (1.05 - 7.24) | 8.58 | 198.21 (47.1 - 321.85) | 260.14 (64.84 - 428.55) | 31.25 |
| Central Latin America | 9.31 (1.77 - 15.64) | 9.68 (2.09 - 16.74) | 3.98 | 333.86 (67.12 - 569.16) | 399.29 (90.88 - 692.8) | 19.60 |
| Central Sub-Saharan Africa | 13.30 (0.54 - 23.93) | 13.03 (0.69 - 23.34) | -2.06 | 367.31 (17.59 - 655.54) | 409.39 (26.58 - 716.04) | 11.46 |
| East Asia | 1.84 (0.22 - 3.24) | 1.93 (0.22 - 3.50) | 5.29 | 86.82 (11.17 - 156.93) | 134.85 (17.33 - 247.24) | 55.33 |
| Eastern Europe | 1.58 (0.33 - 2.40) | 4.47 (1.01 - 6.90) | 183.36 | 107.01 (24.14 - 169.18) | 209.93 (50.23 - 333.66) | 96.17 |
| Eastern Sub-Saharan Africa | 12.47 (1.37 - 21.35) | 9.52 (1.22 - 16.22) | -23.64 | 334.84 (38.10 - 567.24) | 268.20 (38.50 - 457.43) | -19.90 |
| High-income Asia Pacific | 2.21 (0.48 - 3.60) | 0.99 (0.20 - 1.61) | -55.37 | 124.23 (30.05 - 209.07) | 186.59 (43.21 - 321.83) | 50.19 |
| High-income North America | 5.29 (1.13 - 8.22) | 4.54 (1.15 - 6.96) | -14.09 | 208.20 (46.78 - 333.25) | 354.01 (96.56 - 574.67) | 70.04 |
| North Africa and Middle East | 5.69 (1.53 - 9.08) | 6.92 (1.78 - 11.04) | 21.48 | 189.71 (52.62 - 303.57) | 329.22 (95.87 - 548.70) | 73.54 |
| Oceania | 25.90 (4.11 - 44.44) | 26.27 (4.25 - 44.14) | 1.44 | 742.10 (117.64 - 1266.63) | 862.05 (154.31 - 1465.63) | 16.16 |
| South Asia | 5.26 (0.98 - 8.90) | 6.71 (1.42 - 11.08) | 27.52 | 160.21 (31.82 - 272.67) | 234.58 (55.00 - 390.49) | 46.42 |
| Southeast Asia | 4.01 (0.47 - 7.17) | 3.96 (0.64 - 6.91) | -1.20 | 127.61 (15.92 - 226.31) | 149.62 (26.93 - 267.51) | 17.25 |
| Southern Latin America | 8.74 (1.94 - 13.37) | 6.04 (1.47 - 9.22) | -30.95 | 275.29 (67.83 - 426.95) | 314.16 (83.59 - 514.34) | 14.12 |
| Southern Sub-Saharan Africa | 9.73 (1.33 - 16.59) | 18.17 (2.82 - 30.34) | 86.73 | 278.27 (37.91 - 469.53) | 523.89 (83.02 - 886.11) | 88.27 |
| Tropical Latin America | 6.73 (0.85 - 11.64) | 6.47 (0.90 - 11.09) | -3.90 | 236.47 (32.91 - 407.14) | 274.73 (42.57 - 474.23) | 16.18 |
| Western Europe | 5.27 (1.12 - 8.17) | 3.12 (0.70 - 4.83) | -40.85 | 164.08 (38.25 - 259.06) | 195.78 (50.12 - 317.27) | 19.32 |
| Western Sub-Saharan Africa | 7.51 (1.65 - 12.29) | 9.05 (1.98 - 14.73) | 20.49 | 214.05 (46.75 - 354.45) | 290.63 (65.71 - 476.82) | 35.78 |

# Table S7 SDI and GBD regions with an average annual change in mortality and disability-adjusted life years attributed to dietary factors for three chronic diseases from 1990 to 2021

| **location** | **Neoplasms** | **Cardiovascular disease** | **Diabetes mellitus** |
| --- | --- | --- | --- |
|  | **Percentage changes(%)** | **Percentage changes(%)** | **Percentage changes(%)** |
| **Decrease** |  |  |  |
| **Death** |  |  |  |
| Low SDI | -12.19 | -16.87 | -2.54 |
| High-middle SDI | -38.04 | -44.18 | -8.89 |
| High SDI | -35.61 | -61.58 | -27.05 |
| Australasia | -39.55 | -74.01 | -22.66 |
| Caribbean | -7.01 | -40.87 | -21.09 |
| Central Sub-Saharan Africa | -7.59 | -6.92 | -2.06 |
| Eastern Sub-Saharan Africa | -12.83 | -28.96 | -23.64 |
| High-income Asia Pacific | -30.91 | -68.36 | -55.37 |
| High-income North America | -36.95 | -52.94 | -14.09 |
| Southeast Asia | -3.00 | -32.43 | -1.20 |
| Southern Latin America | -23.22 | -62.01 | -30.95 |
| Tropical Latin America | -8.46 | -59.59 | -3.90 |
| Western Europe | -36.50 | -65.77 | -40.85 |
| **DALY** |  |  |  |
| Eastern Sub-Saharan Africa | -18.88 | -32.38 | -19.90 |
| **Increase** |  |  |  |
| **Death** |  |  |  |
| Southern Sub-Saharan Africa | 17.79 | 7.92 | 86.73 |
| **DALY** |  |  |  |
| Southern Sub-Saharan Africa | 14.30 | 0.59 | 88.27 |


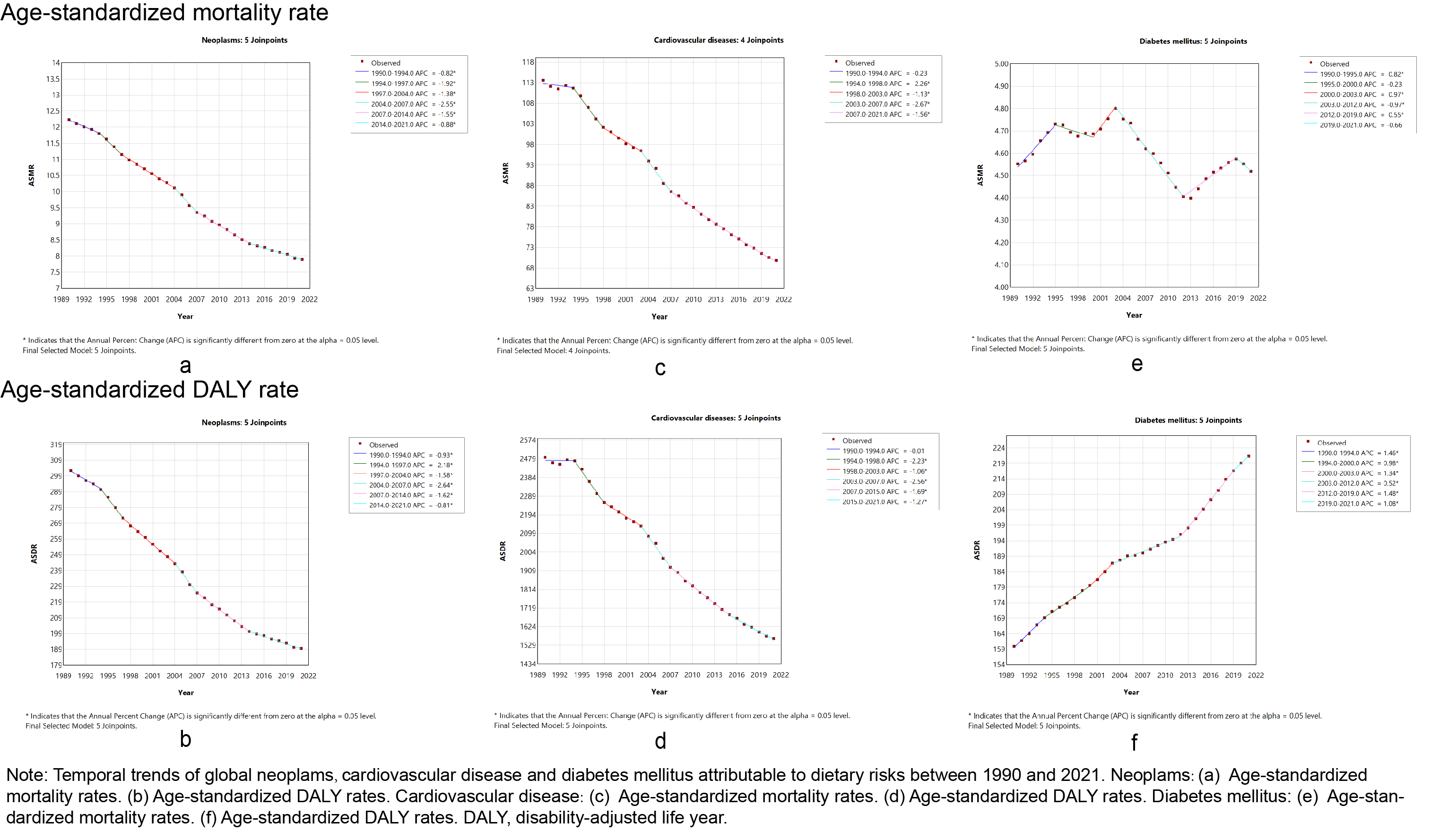


Figure S2 Temporal trends in the global burden of chronic diseases attributable to dietary factors from 1990 to 2021

Note: Temporal trends of global neoplasms, cardiovascular disease and diabetes mellitus attributable to dietary risks between 1990 and 2021. Neoplasms: (a) Age-standardized mortality rates. (b) Age-standardized DALY rates. Cardiovascular disease: (c) Age-standardized mortality rates. (d) Age-standardized DALY rates. Diabetes mellitus: (e) Age-standardized mortality rates. (f) Age-standardized DALY rates. DALY, disability-adjusted life year.


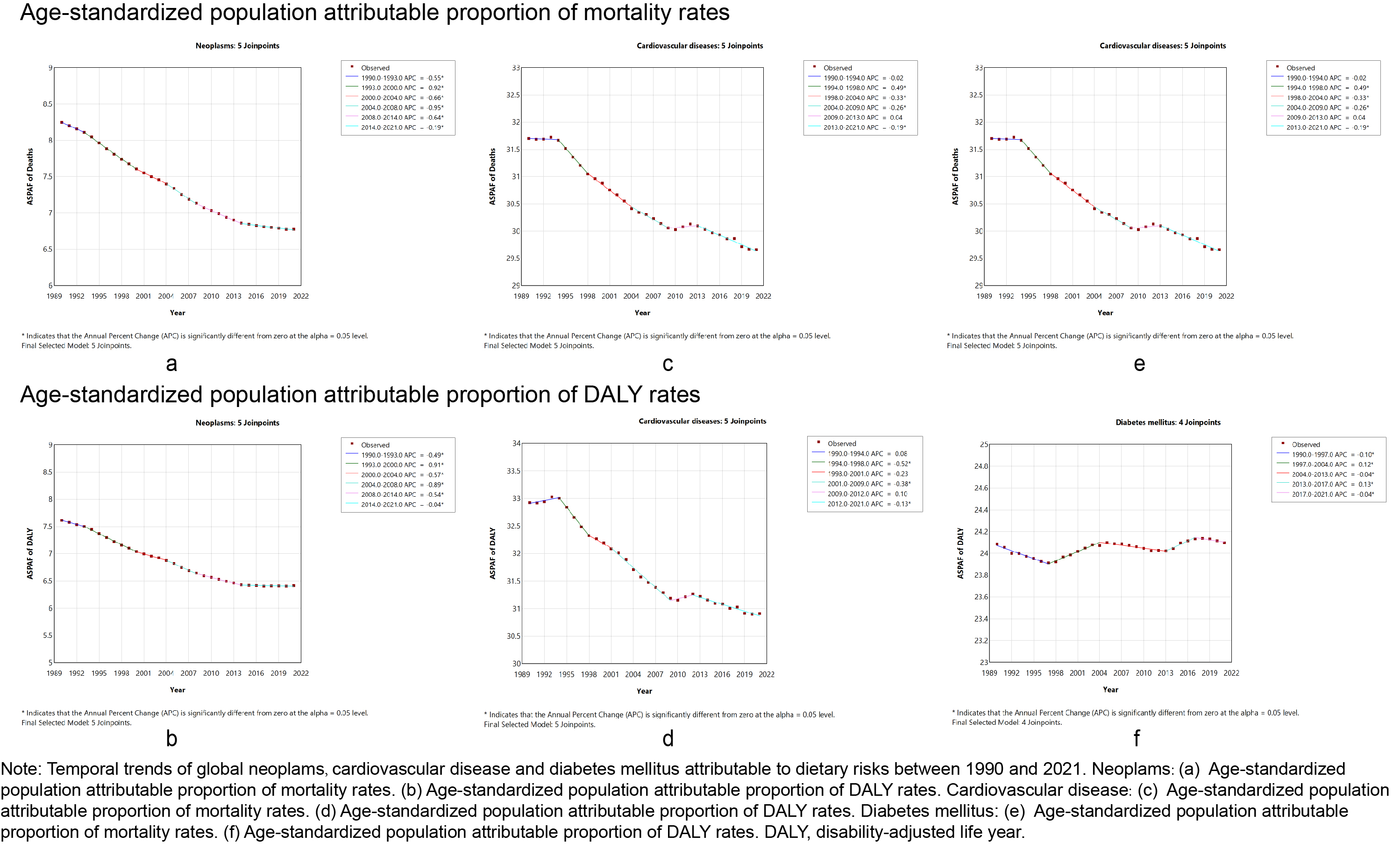


# Figure S3 Temporal trends in the global burden of chronic diseases age-standard population attributable proportion rate to dietary factors from 1990 to 2021

Note: Temporal trends of global neoplasms, cardiovascular disease and diabetes mellitus attributable to dietary risks between 1990 and 2021. Neoplasms: (a) Age-standardized population attributable proportion of mortality rates. (b) Age-standardized population attributable proportion of DALY rates. Cardiovascular disease: (c) Age-standardized population attributable proportion of mortality rates. (d) Age-standardized population attributable proportion of DALY rates. Diabetes mellitus: (e) Age-standardized population attributable proportion of mortality rates. (f) Age-standardized population attributable proportion of DALY rates. DALY, disability-adjusted life year.

# Table S8 Predict the standardized mortality rates of different chronic diseases from 2021 to 2030

| **Age groups** | **Neoplasms** | | | **Cardiovascular disease** | | | **Diabetes mellitus** | | |
| --- | --- | --- | --- | --- | --- | --- | --- | --- | --- |
|  | **2021** | **2030** | **Percentage changes (%)** | **2021** | **2030** | **Percentage changes (%)** | **2021** | **2030** | **Percentage changes (%)** |
| **Total Population** | |  |  |  |  |  |  |  |  |
| 25-29 years | 0.38 | 0.31 | -19.85 | 3.92 | 3.10 | -20.92 | 0.16 | 0.16 | -3.35 |
| 30-34 years | 1.01 | 0.79 | -22.02 | 8.66 | 6.71 | -22.57 | 0.37 | 0.34 | -6.71 |
| 35-39 years | 1.97 | 1.74 | -11.68 | 15.62 | 13.46 | -13.78 | 0.60 | 0.57 | -6.19 |
| 40-44 years | 3.41 | 3.68 | 7.94 | 28.31 | 28.41 | 0.37 | 1.21 | 1.23 | 2.36 |
| 45-49 years | 3.88 | 3.12 | -19.59 | 29.90 | 24.51 | -18.03 | 1.75 | 1.53 | -12.80 |
| 50-54 years | 9.72 | 8.76 | -9.86 | 69.53 | 66.37 | -4.55 | 4.59 | 4.81 | 4.72 |
| 55-59 years | 15.21 | 14.34 | -5.74 | 110.28 | 102.86 | -6.73 | 7.51 | 7.85 | 4.58 |
| 60-64 years | 22.12 | 22.11 | -0.08 | 167.80 | 159.57 | -4.90 | 12.85 | 13.52 | 5.19 |
| 65-69 years | 31.90 | 31.33 | -1.76 | 247.80 | 236.27 | -4.65 | 18.44 | 20.38 | 10.54 |
| 70-74 years | 45.42 | 42.62 | -6.15 | 358.10 | 331.37 | -7.47 | 26.39 | 28.45 | 7.83 |
| 75-79 years | 61.22 | 59.45 | -2.89 | 527.46 | 474.27 | -10.08 | 38.98 | 39.70 | 1.85 |
| 80-84 years | 86.07 | 77.56 | -9.89 | 853.33 | 680.00 | -20.31 | 53.51 | 51.78 | -3.23 |
| 85-89 years | 121.45 | 106.98 | -11.92 | 1414.56 | 1124.20 | -20.53 | 77.57 | 77.41 | -0.21 |
| 90-94 years | 169.25 | 156.54 | -7.51 | 2196.96 | 1865.22 | -15.10 | 110.88 | 114.27 | 3.05 |
| 95+ years | 207.91 | 192.55 | -7.39 | 2894.24 | 2548.40 | -11.95 | 139.26 | 151.40 | 8.71 |
| Total | 10.52 | 9.81 | -6.73 | 95.24 | 83.37 | -12.46 | 6.10 | 6.26 | 2.71 |
| **Male** |  |  |  |  |  |  |  |  |  |
| 25-29 years | 0.35 | 0.27 | -22.75 | 5.12 | 4.15 | -18.87 | 0.15 | 0.15 | 3.16 |
| 30-34 years | 0.92 | 0.69 | -24.97 | 12.01 | 9.62 | -19.83 | 0.43 | 0.44 | 2.40 |
| 35-39 years | 1.73 | 1.53 | -11.60 | 21.83 | 19.70 | -9.75 | 0.66 | 0.68 | 2.93 |
| 40-44 years | 3.05 | 3.43 | 12.42 | 39.97 | 41.57 | 4.00 | 1.36 | 1.47 | 7.76 |
| 45-49 years | 3.58 | 2.93 | -18.16 | 40.10 | 34.34 | -14.37 | 1.92 | 1.76 | -8.37 |
| 50-54 years | 9.93 | 8.70 | -12.44 | 97.14 | 93.29 | -3.97 | 5.12 | 5.36 | 4.81 |
| 55-59 years | 16.45 | 15.25 | -7.31 | 148.26 | 139.39 | -5.98 | 8.26 | 8.59 | 4.03 |
| 60-64 years | 25.17 | 24.85 | -1.30 | 224.57 | 211.05 | -6.02 | 13.59 | 14.06 | 3.51 |
| 65-69 years | 37.44 | 35.55 | -5.05 | 320.13 | 303.56 | -5.18 | 19.32 | 21.22 | 9.84 |
| 70-74 years | 53.19 | 48.60 | -8.63 | 448.74 | 414.22 | -7.69 | 28.07 | 30.19 | 7.56 |
| 75-79 years | 70.21 | 68.39 | -2.60 | 636.82 | 573.51 | -9.94 | 41.08 | 42.28 | 2.92 |
| 80-84 years | 97.46 | 87.56 | -10.16 | 986.53 | 789.76 | -19.95 | 55.09 | 55.52 | 0.79 |
| 85-89 years | 140.73 | 119.81 | -14.87 | 1739.9 | 1332.78 | -23.4 | 87.34 | 86.49 | -0.98 |
| 90-94 years | 176.65 | 161.91 | -8.34 | 2620.8 | 2183.88 | -16.67 | 125.11 | 124.64 | -0.37 |
| 95+ years | 173.79 | 159.92 | -7.98 | 2863.9 | 2509.03 | -12.39 | 142.24 | 147.55 | 3.73 |
| Total | 11.63 | 10.70 | -8.01 | 118.46 | 103.51 | -12.62 | 6.54 | 6.73 | 2.82 |
| **Female** |  |  |  |  |  |  |  |  |  |
| 25-29 years | 0.43 | 0.36 | -15.78 | 2.69 | 2.04 | -23.99 | 0.18 | 0.17 | -5.82 |
| 30-34 years | 1.10 | 0.90 | -17.98 | 5.25 | 3.88 | -26.08 | 0.30 | 0.27 | -9.52 |
| 35-39 years | 2.22 | 1.97 | -10.99 | 9.31 | 7.42 | -20.21 | 0.54 | 0.48 | -10.7 |
| 40-44 years | 3.77 | 3.95 | 4.77 | 16.54 | 15.54 | -6.05 | 1.05 | 1.03 | -1.62 |
| 45-49 years | 4.19 | 3.33 | -20.59 | 19.01 | 14.39 | -24.31 | 1.56 | 1.31 | -15.92 |
| 50-54 years | 9.52 | 8.84 | -7.15 | 42.05 | 39.85 | -5.22 | 4.07 | 4.27 | 4.78 |
| 55-59 years | 14.02 | 13.47 | -3.90 | 73.50 | 67.48 | -8.19 | 6.80 | 7.17 | 5.48 |
| 60-64 years | 19.24 | 19.58 | 1.78 | 114.14 | 110.98 | -2.77 | 12.16 | 12.96 | 6.60 |
| 65-69 years | 26.83 | 27.55 | 2.69 | 181.59 | 175.07 | -3.59 | 17.64 | 19.40 | 9.96 |
| 70-74 years | 38.58 | 37.97 | -1.56 | 278.27 | 258.07 | -7.26 | 24.90 | 26.57 | 6.70 |
| 75-79 years | 53.75 | 53.39 | -0.66 | 436.77 | 393.55 | -9.89 | 37.23 | 37.09 | -0.38 |
| 80-84 years | 77.88 | 71.83 | -7.78 | 757.46 | 604.33 | -20.22 | 52.38 | 48.74 | -6.94 |
| 85-89 years | 109.76 | 100.43 | -8.50 | 1217.4 | 1014.37 | -16.68 | 71.70 | 72.04 | 0.48 |
| 90-94 years | 165.66 | 152.31 | -8.06 | 1992.11 | 1730.69 | -13.12 | 104.13 | 109.61 | 5.26 |
| 95+ years | 220.66 | 199.52 | -9.58 | 2905.90 | 2571.55 | -11.51 | 138.92 | 153.02 | 10.15 |
| Total | 9.61 | 9.15 | -4.71 | 75.79 | 66.45 | -12.33 | 5.75 | 5.86 | 1.93 |


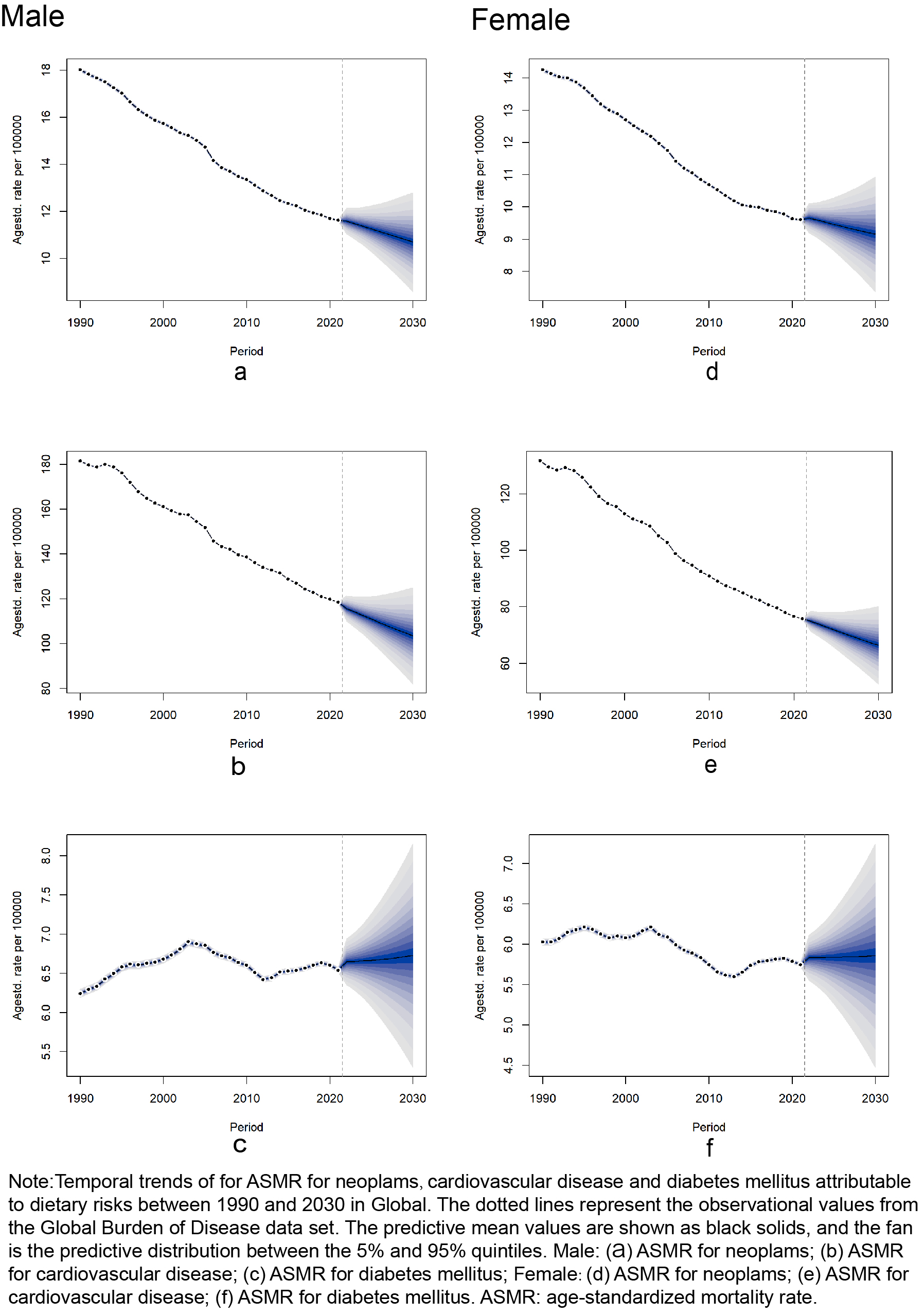


# Figure S4 Temporal trends of for ASMR for neoplasms, cardiovascular disease and diabetes mellitus attributable to dietary risks among different gender between 1990 and 2030 in Global

Note: Temporal trends of for ASMR for neoplasms, cardiovascular disease and diabetes mellitus attributable to dietary risks between 1990 and 2030 in Global. The dotted lines represent the observational values from the Global Burden of Disease data set. The predictive mean values are shown as black solids, and the fan is the predictive distribution between the 5% and 95% quintiles. Male: (a) ASMR for neoplasms; (b) ASMR for cardiovascular disease; (c) ASMR for diabetes mellitus; Female: (d) ASMR for neoplasms; (e) ASMR for cardiovascular disease; (f) ASMR for diabetes mellitus. ASMR: age-standardized mortality rate.
